# Electron-Donating *para*-Substituent (X) Enhances the Water Oxidation Activity of the Catalyst Ru(4′-X-terpyridine)(phenanthroline-SO_3_)^+^

**DOI:** 10.1021/acs.inorgchem.4c04124

**Published:** 2025-02-07

**Authors:** Miguel A. Ibañez, Colton J. Breyer, Milan Gembicky, Zinnun F. Malikov, Djamaladdin G. Musaev, Douglas B. Grotjahn

**Affiliations:** † Department of Biochemistry and Chemistry, San Diego State University, San Diego, California 92182-1030, United States; ‡ Emerson Center for Scientific Computation and Department of Chemistry, Emory University, Atlanta, Georgia 30322, United States; § Department of Biochemistry and Chemistry, University of California-San Diego, San Diego, California 92093, United States; ∥ Innovation Academy, 125 Milton Ave, Alpharetta, Georgia 30009, United States

## Abstract

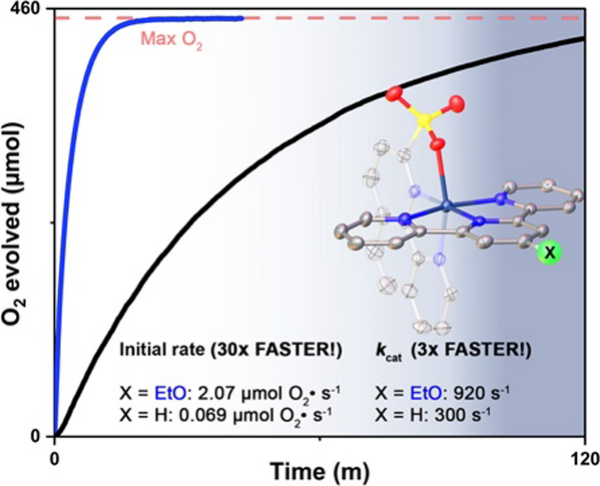

Recently, our group has developed a Ru-based water oxidation catalyst (WOC) with pendant sulfonate (**1,** Ru(4′-X-terpyridine)(phenanthroline-SO_3_)OTf (X = H, **1a**) that shows high activity under both sacrificial oxidant (CAN, Ce(NH_4_)_2_(NO_3_)_6_, Ce^IV^) and electrocatalytic conditions, in both acidic and neutral media. Here, we demonstrate that the functionalization of the 4′-X-terpyridine ligand with an electron-donating substituent X = OEt **(1b)** makes potentials of Ru^II^/Ru^III^ redox catalysis more negative, whereas when X = NO_2_**(1c)** and CF_3_**(1d)**, potentials are more positive. For **1b,** full conversion of the sacrificial oxidant Ce^IV^ occurred in 0.4 h (7 h for **1a**), with an initial rate of 2.07 μmol O_2_ s^–1^ and a turnover frequency of 7.6 s^–1^, which is 30-fold faster than that for **1a** at [cat]_0_ = 20 μM. Under electrocatalytic conditions, water oxidation by **1b** is three times faster than that by the parent catalyst **1a** at close to the same potential. Extensive computations have identified differences in the initial PCET steps of the water oxidation by catalysts **1a, 1b,** and **1d**, and demonstrated the increased probability of the O_2_ formation via the oxide relay pathway in the order **1b**< **1a** < **1d**.

## Abstract 2

The developed Ru-based WOC with pendant sulfonate Ru(4′-X-terpyridine)(phenanthroline-SO_3_)OTf shows high activity under both sacrificial oxidant and electrocatalytic conditions in both acidic and neutral media. The functionalization of 4′-X-terpyridine ligand with an electron-donating substituent X = OEt makes the water oxidation by this system several times faster than that by the parent catalyst. Computations show that two sequential ETPT’s, in the presence of CAN, transform Ru^II^-aqua complexes into the associated Ru^III^-oxyl and Ru^V^-peroxo complexes.

## Introduction

Over the last 20 years, there has been a 12% increase in greenhouse gases in the earth’s atmosphere^1^; therefore, the search for an alternative source of energy with low greenhouse emissions to replace current carbon-based fuel is a global priority. One of the promising alternatives for carbon-based fuel sources is hydrogen-based fuel because hydrogen has an energy density about two times greater than that of gasoline. The current predominant method for hydrogen production is steam reforming technology. This energy-demanding process uses hydrocarbon feedstocks to generate the H_2_ molecule. Unfortunately, it also generates greenhouse gas byproducts.^2^ Under these circumstances, the search for green, environmentally benign, and more efficient alternative methods for H_2_ production is becoming a vital research direction. Today, such technology is water splitting, i.e., the splitting of water molecules into oxygen and protons. This process is key in photosynthesis^3^ and has inspired the development of numerous molecular water oxidation catalysts (WOCs), including, but not limited to, the blue dimer,^4−6^ as well as multiple mono-Ru-complexes^7−23^ and polyoxometalate^24−26^-based catalysts. For example, Sun and co-workers have reported a Ru(2,2′-bypyridine-6,6′-dicarboxylic acid), **2**, catalyst with different axial ligands.^9^ It was shown that the catalyst functionalized with two axial isoquinoline ligands, i.e., catalyst **2_(isoq)**_**2**_, performs better than the analog containing two axial picoline ligands under the sacrificial oxidant testing conditions,^9^ with a TON of 8360 and TOF of 1120 s^–1^ at [cat]_0_ = 15 μM. The increase in performance of **2_(isoq)**_**2**_ relative to its picoline analog, was attributed to the existence of the stabilizing pi-pi stacking between the isoquinoline rings at the rate-limiting O–O formation transition state. Llobet’s group in 2015 reported^21^ the [Ru(tda)(pic)_2_], **3**, catalyst, which performed extremely well under electrochemical conditions, with a TOF of 8000 s^–1^ in pH = 7. Its high performance is hypothesized to arise from a pendant carboxylate anion assisting water nucleophilic attack at metal oxo, while the role of the oxide relay concept should not be underestimated.^27,28^ Similarly, in 2017, Concepcion^11^ demonstrated that a labile pendant base could help mediate intramolecular proton-coupled electron transfer (PCET). In 2019, Yagi and co-workers^12^ unraveled the critical role of electron donor and withdrawing groups (X) of the 4′-X-terpyridine ligand on the water oxidation activity of the parent compound, the [Ru(X-terpy)(bpy)OH_2_]^2+^ complex, where (X-terpy and bpy are 2,2′;6′,2″-terpyridine and 2,2′-bipyridine derivatives). Inspired by these findings, we hypothesize that modifying the electrostatics of the terpyridine ligand while retaining the external sulfonate base on our existing ruthenium catalyst system will lower the oxidation potential needed for the PCET step during the water oxidation process.

In 2021, our group published a catalyst^22^ [Ru(terpyridine)(phenanthroline-SO_3_)OTf], **1a,** that maintains high electrocatalytic turnover frequency at pH values as low as 1.1 and 0.43 (*k*_cat_ = 1501 ± 608 and 831 ± 254 s^–1^, respectively). This catalyst also exhibits excellent durability when a chemical oxidant is used at [cat]_0_ = 5 μM (Ce^IV^, TON = 7400, and TOF = 0.88 s^–1^). Being able to perform water oxidation in acidic conditions is a particularly vital feature of this catalyst because proton reduction is a facile process under acidic conditions, so catalyst stability in acidic media is necessary. The functionalization of the bidentate ligand with a pendant sulfonate increased the water oxidation activity of the catalyst by 40 to 100-fold.^22^

In the current study, which is motivated by previous data suggesting that functionalization of the auxiliary ligand by electron-donating substituents improves catalysis,^9,11,12,20^ we study the impacts of electron donor and withdrawing groups (X) of the 4′-X-terpyridine ligand on the water oxidation activity of the parent compound **1a**.

For this reason, (a) we have synthesized and characterized **1b** (X = OEt), **1c** (X = NO_2_), and **1d** (X = CF_3_) derivatives of the previously reported^22^ catalyst [Ru(4′-X-terpyridine)(phenanthroline-SO_3_)OTf], **1a,** with X = H, (b) studied their water oxidation activities under the sacrificial oxidant Ce^IV^ and electrocatalytic conditions, in both acidic and neutral media, and (c) elaborated impact of the 4′-X-substitution on the structure of these catalysts, the nature of the initial PCET steps, and the catalytic active Ru^V^-oxo and Ru^III^-peroxo intermediates generation using DFT calculations.

**Figure 1:**
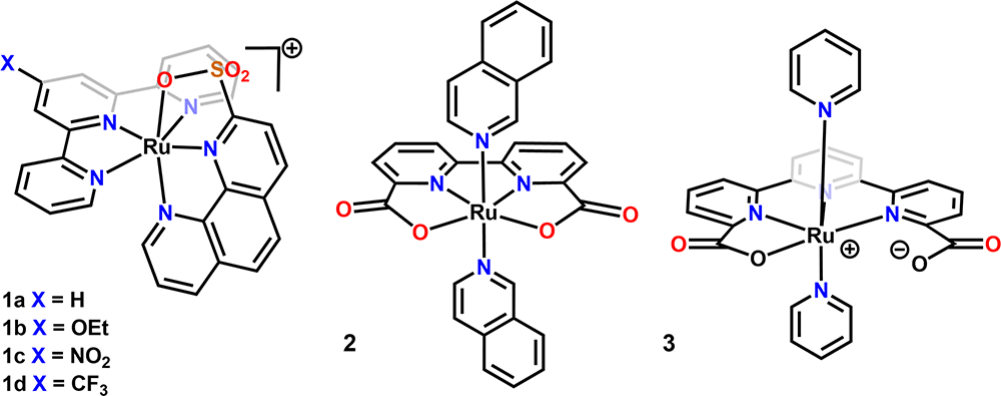
Mono-Ru-based water oxidation catalysts (WOC) **1b**–**1d** with pendant sulfonate reported in this paper and other recently reported analogs.

Briefly, we have extended a mono-Ru-based WOC family with pendant sulfonate (**1,** Ru(4′-X-terpy)(phenanthroline-SO_3_)OTf (X = H, **1a**) and prepared and characterized its derivatives **1b** (with X = OEt), **1c** (with X = NO_2_), and **1d** (with X = CF_3_) (Figure 1). We have established that catalyst **1b** with the electron-donating X = OEt substituent shows even higher activity under both sacrificial oxidant Ce(IV) and electrocatalytic conditions in both acidic (pH = 1.1) and neutral (pH = 7) media. Extensive computations have identified differences in the initial PCET steps of the water oxidation by catalysts **1a, 1b,** and **1d**, and demonstrated the increased probability of the formation of the O_2_ via the oxide relay pathway (initiated from the Ru^III^-peroxo intermediate) in the order **1b**< **1a** < **1d.**

### Catalyst Synthesis and Structure

Synthesis of **1b**–**d** was performed via similar procedures (similar to the synthesis of the previously reported^22^ parent compound **1a**). We use Ru(Cl_2_)(DMSO)_4_, **C1**, as a ruthenium source and the X-terpyridine scaffold (where X = OEt, NO_2_, and CF_3_, for **1b**–**d**, respectively). The materials were suspended in absolute ethanol under a N_2_ flow. The suspension was heated to 90 °C for 24 h and cooled to room temperature. The resulting red suspension was filtered through a fine frit and washed sequentially with cold ethanol, deionized water, and diethyl ether. The washed solid was then subjected to a vacuum and yielded a fine red powder. Elemental analysis confirmed the anticipated molecular composition. NMR experiments also confirmed the successful coordination of the ligand scaffold to the ruthenium metal to afford the complex [Ru(Cl_2_)(DMSO)(EtO-terpy)], **C2**, in a 79% yield. In the glovebox, the complex **C2** and silver triflate were suspended in absolute ethanol and stirred at room temperature for ∼1 h. The red suspension was filtered by vacuum filtration using a fine frit in the glovebox, leaving a purple residue behind on the frit. The filtrate retained was added to a pressure vessel, to which NaOH and phenanthroline-2-sulfonic acid were added. The mixture was brought from the glovebox and heated to 100 °C for 24 h. The red suspension was cooled to RT and then to 0 °C. The mixture was filtered through a fine frit and washed with cold ethanol, followed by cold diethyl ether. The filtrate was disposed of, and the retained solids were dissolved using an excess of acetone in portions to wash all products through the frit. The filtrate was concentrated by roto evaporation and dried using P_4_O_10_ under vacuum overnight in 49% yield. The fine red powder was characterized by using elemental analysis and NMR.

**Figure 2:**
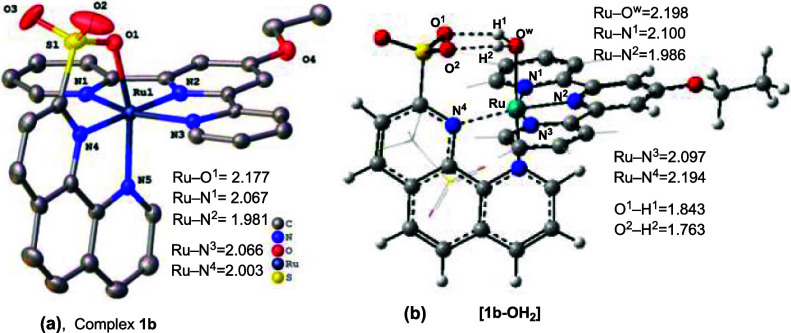
(a) X-ray structure of **1b**, and (b) the calculated molecular structure of **[1b–OH**_**2**_**]**. All given geometry parameters are in Å. For simplicity, some Hydrogen atoms are either removed or “dimmed”.

We crystallized **1b** and **1c** from MeOH and analyzed these structures by X-ray diffraction (see Figure 2a for the structure of **1b** and Supporting Information for the structure of **1a** and **1c** for more details). As seen in Figure 2a, ligand sulfonate is coordinated to the Ru(II)-center with a Ru–O bond distance of 2.177 (2) Å. However, calculations (see Figure 2b) suggest the coordination of a water molecule to the same Ru(II) center (the addition of a water molecule to complexes **1a, 1b,** and **1d** is exergonic by Δ*G*° = −5.4, −5.5, and −4.6 kcal/mol, respectively; see below for more details). In the aqua complex [**1b–OH**_**2**_], the (a) calculated Ru–OH_2_ bond distance is 2.198 Å, and the (b) Ru(II)-coordinated water molecule forms two O_2_SO---H bonds with the SO_3_-unit of the pendant ligand.

These H-bonding interactions weaken the O–H bonds of the water molecule, which is manifested in the change of the calculated (unscaled) O–H bond stretches, which are 3455 and 3550 cm^–1^ for [**1b–OH**_**2**_]. The calculated (unscaled) O–H bond stretches are 3800 and 3902 cm^–1^ for free water molecules. Previously we reported^22^ for [**1a–OH**_**2**_], IR(ATR) showed two sharp stretches at 3463.3 and 3547.4 cm^–1^. Thus, computations enabled us to formulate the catalyst as an aqua complex [**1b–OH**_**2**_] in homogeneous catalytic conditions.

### Catalyst Performance under Sacrificial Oxidant Conditions

**Figure 3:**
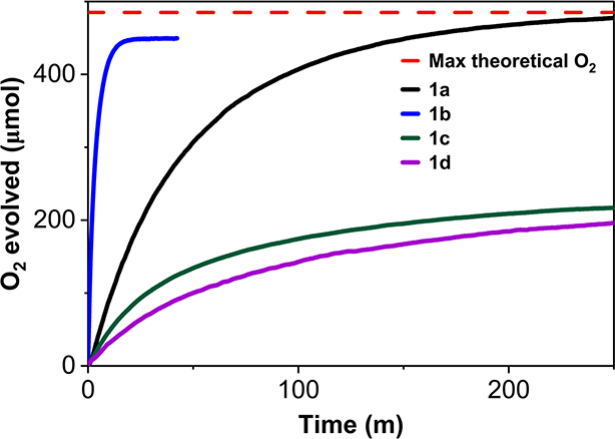
Oxygen evolution of catalyst **1a**–**d** at 20 μM [cat]_0_. The catalyst solution was prepared in acetonitrile and injected into a 0.2 μM solution of Ce^IV^ in 0.1 M HNO_3_. Temperature and pressure were allowed to stabilize prior to the injection of the catalyst. Catalyst solutions were prepared by weighing solids using a microbalance and dissolving them in reagent grade acetonitrile to create a stock solution. From the stock solution, the appropriate volume was obtained using a micro syringe and injected into the testing vessel containing the Ce^IV^ solution once equilibrium was reached. The temperature of the reaction vessel was held constant (*T* = 30 °C) using a Lauda bath system. The full sacrificial oxidant setup can be found in Figure S2 of the Supporting Information.

Experiments were performed using ceric ammonium nitrate ([CAN]_0_ = 0.20 M) dissolved in 0.1 M HNO_3_ at varying catalyst concentrations. Rates of oxygen evolution for catalysts **1a**–**d** can be seen in Figure 3.

As seen in Figure 3 and Table 1, for [**1b**]_0_ = 20 μM, 94 ± 2.4% of theoretical O_2_ generation was achieved in 0.4 h, and TON achieved was 2010, with an initial rate of 2.07 μmol O_2_ s^–1^, i.e., the complex **1b** has an initial rate 30x faster than the unsubstituted complex **1a**. At 40 μM of **1b**, 97 ± 2.4% consumption of sacrificial oxidant was achieved in 0.26 h with a TON of 1180 and an initial rate of 3.40 μmol O_2_ s^–1^. At the highest concentration of **1b** of 80 μM, full consumption of sacrificial oxidant was accomplished in 0.2 h with a TON of 600 and an initial rate of 5.74 μmol O_2_ s^–1^. At the lowest concentration of **1b,** [cat]_0_ = 5 μM, the TON achieved was 9200 with an initial rate of 0.46 μmol O_2_ s^–1^ compared to **1a,** which at the same concentration had a TON of 7400 and an initial rate of 0.041 μmol O_2_ s^–1^ (see Table S1). It is important to note that **1a** appeared to require additional time in solution prior to turning over indicated by a lag time between pressure measurements. This may be explained by the significantly slower rate of oxygen generation, causing oxygen to saturate the solution prior to entering the headspace.

**Table 1:** Catalytic Values from Sacrificial Oxidant Testing^a^

[cat]_0_		theoret. O_2_ achieved (%)	avg SD (σ)	initial rate (μmol O_2_·s^–1^) ± 0.02	TON (h)	TOF (s^–1^)	*R*^2^
**1a**^b^	(20 μM)	98	±5	0.07	2400 (7)	0.88	0.992
**1b**	(20 μM)	94	±2.4	2.08	2010 (0.4)	7.69	0.997
	(40 μM)	97		3.40	1180 (0.3)		0.998
	(80 μM)	100		5.74	600 (0.2)		0.995
**1c**	(20 μM)	47	±1.4	0.10	1130 (12)	0.54	0.998
	(40 μM)	72		0.18	820 (12)		0.984
	(80 μM)	100		0.39	540 (12)		0.992
**1d**	(20 μM)	47	±2.2	0.10	1170 (12)	0.33	0.998
	(40 μM)	72		0.21	880 (12)		0.999
	(80 μM)	94		0.50	530 (12)		0.999

a The turnover frequency (TOF) of catalysts was determined by taking the slope of the initial rate of μmol O_2_ s^–1^ w.r.t to catalyst concentration (see the data graphed in Figure S6). Sacrificial oxidant testing was done using a 0.2 μM solution of ceric ammonium nitrate in 0.1 M nitric acid. The reaction temperature was held at 30 °C. Experiments were performed in triplicate to ensure reproducibility. Error reported for the theoretical O_2_ achieved for **1a**–**1d** was determined by the average standard deviation (S.D.).

b See ref (22) for **1a** data.

For **1c**, at [cat]_0_ = 20 μM, 47 ± 1.4% consumption of sacrificial oxidant was achieved in 12 h with a TON of 1130 and an initial rate of 0.095 μmol of O^2^ s^–1^. Similarly, **1d** achieved 47 ± 2.2% consumption of sacrificial oxidant in 12 h with a TON of 1170 and an initial rate of 0.096 μmol O_2_ s^–1^ when [cat]_0_ = 20 μM. These results indicate that the addition of an electron-donating ethoxy group to the terpyridine scaffold of **1a** greatly enhances the reaction rate. These findings led us to explore the effects of the electron donor groups more on the catalysts’ water oxidation capability and their performance under electrochemical conditions. The rate of reaction was determined to be first-order both with respect to the catalyst when varying [cat]_0_ and at a constant concentration of 0.2 M CeIV (see Figure S6) and with respect to the sacrificial oxidant at varying Ce^IV^ concentrations with a constant 20 μM concentration of **1b** (see Figure S7).

### Catalyst Performance under Electrochemistry Conditions

**Figure 4:**
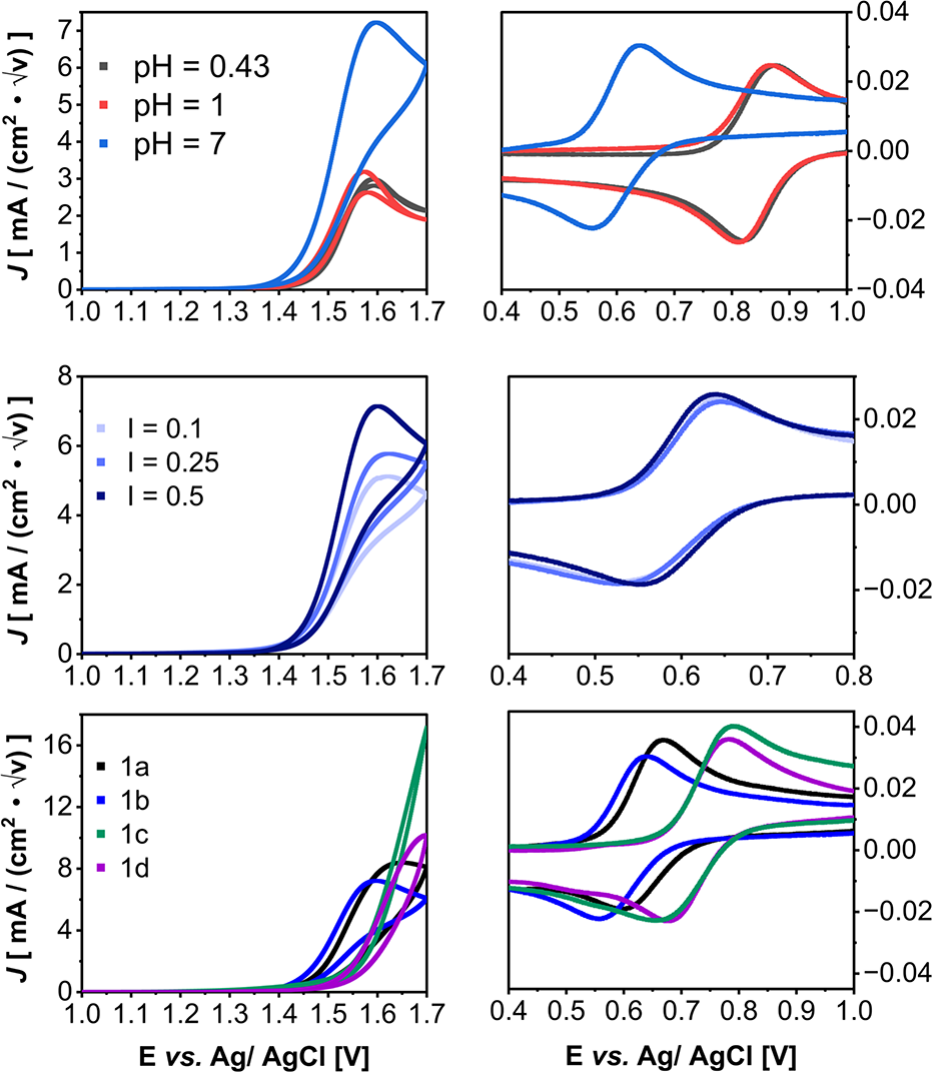
(a) Cyclic voltammetry experiments of **1b** demonstrating catalytic activity and reversible Ru(II/III) couple at varying pH conditions and *I* = 0.5. (b) CV of **1b** at scan rate = 0.1 V s^–1^ at pH = 7 and varying phosphate buffer concentration represented in ionic strength. (c) Cyclic voltammetry experiment overlay of **1a**–**1d** at pH = 7 and *I* = 0.5, and scan rate = 0.1 V s^–1^_._ Experiments were performed in triplicate to ensure reproducibility. The reported data above was normalized by dividing the current by the square root of the scan rate used. CV experiments were done using a single compartment cell, at room temperature, starting point 0 V, scan rate = 0.1 V s^–1^, scanning positive, using a boron-doped diamond working electrode (WE), Ag/AgCl reference electrode (RE), and platinum counter electrode (CE). Complete electrochemical conditions can be found in Figure S2 of the Supporting Information.

Cyclic voltammetry experiments (Figure 4) were conducted using a boron-doped diamond working electrode, an Ag/AgCl reference electrode, and a Pt wire counter electrode. Scan rates ranging from 20 to 0.01 V s^–1^ were performed to determine the scan rate dependency. Buffer solutions at pH = 7 were made using monobasic and dibasic phosphate, and ionic strength was adjusted using potassium nitrate. Experiments done at pH = 1 were acidified using 0.1 M nitric acid. Cyclic voltammetry experiments were all background subtracted. A less positive redox potential for **1b** was observed in neutral pH and ionic strength *I* = 0.5, compared to **1a** and **1c**–**d**.

The lower potential of the Ru (II/III) couple for **1b** is consistent with the ethoxy group donating electron density to the terpyridine, coordinating with the ruthenium center. As for catalysis, one sees nearly equal currents at pH = 1 and 0.43 (Figure 4a) but greatly enhanced currents at pH = 7, all at an ionic strength of *I* = 0.5. Meyer and co-workers^16^ and Wang and Groves^29^ both described the base enhancement effect, and here, we see similar results for both **1a** and **1b**.

The catalytic values from electrochemical experiments, *k*_cat_, are determined by a method illustrated in the Supporting Information (Figures S15–S20), but a summary is provided in Table 2. Five scan rates from 0.025 to 0.25 V/s and three phosphate buffer concentrations from 0.047 to 0.223 M were used to determine the pseudo-limiting current, *E*_cat*,*_ potentials. Originally, four phosphate buffer concentrations were used, including 0.017 M. However, at low scan rates, the buffer capacity was exceeded due to the generation of protons near the electrode surface, causing large deviations in current, and therefore this buffer concentration was omitted from the overall method, see Figure S19. The rate constant for catalysis was split into a two-term rate law discussed in 2010 and 2015 papers from the Meyer group^16,30^ using the two-term rate law:
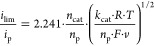
1
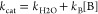
2where *i*_lim_ is the limiting current of catalysis in amps, *n*_cat_ is the number of electrons transferred in catalysts, *F* is Faraday’s constant in C/mol, *v* is the scan rate in V/s, *R* is the gas constant in J/Kmol, *T* is the temperature in K, *k*_cat_ is the forward rate constant of the rate-limiting step of catalysis, *k*_H2O_ is the rate constant of water nucleophilic attack that is unassisted by buffer base B, and *k*_B_ is the rate constant of the phosphate base-assisted reaction.

**Table 2:** Catalytic Values from Electrochemical Experiments^a^

cat	pH	*k*_B_ (M^–1^ s^–1^)	*k*_H2O_ (S^–1^)	*k*_cat_ (S^–1^)	*E*_cat_ (V)	*E*_Ru(II)/(III)_ (V)	onset (V)
**1a**	7	2040 ± 330	170 ± 50	300	1.53	0.64	1.33
**1b**	1		420 ± 20	n/a	1.52	0.85	1.38
**lb**	7	4300 ± 610	520 ± 90	920	1.52	0.61	1.27

a Comparison of **1a** and **1b** at varying pH and corresponding electrochemical values. *E*_cat_ is the optimized potential picked for pseudo-limiting current analysis. *E*_1/2_ is the half-wave potential of the ruthenium(II/III) reversible couple. *E*_onset_ is the potential at which catalysis begins.

To determine *k*_cat_ as a function of the base, Meyer and co-workers used limiting current methods. They determined the value of *E*_cat_ by looking for the CV that was the best approximation to a limiting current. Other researchers, such as the Llobet group,^31^ for example, used foot-of-the-wave analysis (FOWA) to determine catalytic rate constants. Still, the value obtained by either method depends on the particular parameters used (Figure S17). Here we use the value of *E*_cat_ that gives the best global linear fit in a series of plots of both *i*_cat_/*i*_p_ vs *v*^–1/2^ and *k*_cat_ vs [phosphate] (Figures S18–S20). Additional details on the determination of *k*_cat_ may be found in the Supporting Information (Figures S15–S20).

We should note that for **1a**, the value of *E*_cat_, at which *k*_cat_ was determined in our previous paper, has deviated by 220 mV.^21^ Initially, this potential was determined at the maximum current observed, 1.75 V, whereas here, its value is optimized to be 1.53 V. Regardless of ionic strength, values for the potentials of both the Ru^II^/Ru^III^ couple and *E*_cat_ were lower for the ethoxy analog compared to the values for the parent compound. The lower potentials are likely a result of a more electron-rich metal center. Retention of catalytic activity in a broad range of pH is significant because of how proton reduction is facilitated in acid.^22^

To summarize, here, we synthesized and characterized various derivatives of the previously reported Ru-based WOC, **1,** Ru(4′-X-terpyridine)(phenanthroline-SO_3_)OTf with pendant sulfonate. We demonstrated that the functionalization of the 4′-X-terpyridine ligand of **1** with an electron-donating substituent X = OEt **(1b)** makes potentials of Ru^II^/Ru^III^ redox catalysis more negative, whereas when X = NO_2_**(1c)** and CF_3_**(1d)**, potentials are more positive. For **1b,** full conversion of the sacrificial oxidant Ce^IV^ occurred in 0.4 h (7 h for X = H, **1a**), with an initial rate of 2.07 μmol O_2_ s^–1^, a turnover frequency of 7.6 s^–1^, and a TON of 2010. Under electrocatalytic conditions, at pH = 7, **1b** had an onset potential of 1.27 V, *E*_1/2_ = 0.608 V, *k*_cat_ = 920 s^–1^ when [cat]_0_ = 50 μM, and is 3x faster than that for **1a** at [cat]_0_ = 20 μM. We attribute the observed significant rate improvement in both the sacrificial oxidant and the electrochemical conditions to the (a) combination of an electron-rich terpyridine scaffold that lowers the oxidation potential of the metal center and the decrease in the Lewis basicity of the ligand due to the presence of the sulfonate pendant base on phenanthroline and (b) partly, as previously demonstrated,^32^ participation of the in situ generated Ce-radical species in the sacrificial oxidant conditions in heterometallic O–O bond formation”

### Computational Analyses

**Figure 5:**
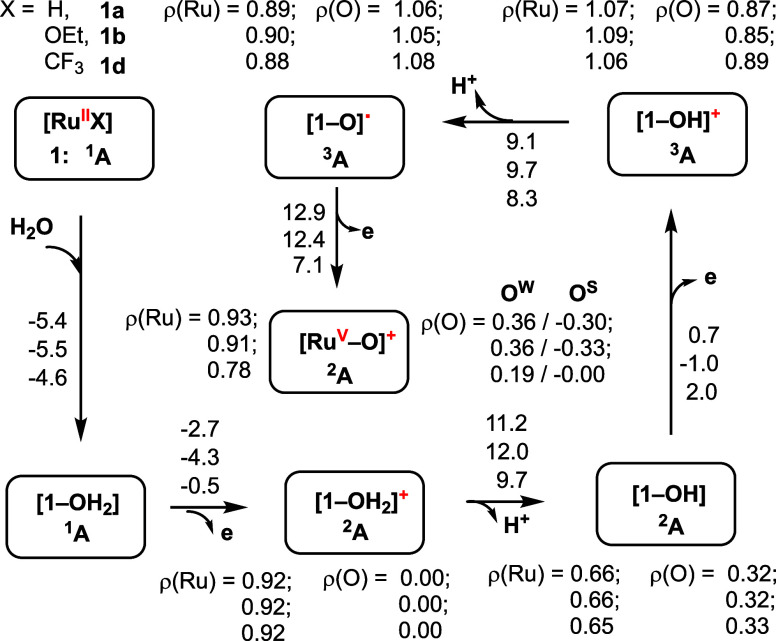
Energies of the first five steps of the water oxidation by **1a** (X = H), **1b** (X = OEt), and **1d** (X = CF_3_) catalysts driven by CAN (ceric ammonium nitrate). [Ru^II^] is the water-free structure seen in the X-ray analyses (see Figure 2a). Δ*G*° values from [**1-OH**_**2**_] to [**1-O·**] or to **[Ru**^**V**^**–O]**^**+**^ for X = H, OEt, CF_3_ are +7.5, + 5.4, + 10.3, and 20.4, 17.8, 17.4 kcal/mol, respectively.The relative energies (free energies given in kcal/mol) are calculated relative to the associated previous steps. Spin densities (ρ) of each intermediate are given in |e|. The calculated structures of all reported species are given in Figures S31 and S32 of the Supporting Information.

Previously, we have proposed^22^ that the initial steps of water oxidation by diamagnetic Ru^II^(4′-X-terpy)(phen-SO_3_)(OTf), with X = H (**1a**) in the presence of CAN, are the water coordination to the Ru(II) center, and the following two consequent PCET events which may occur either stepwise, i.e., electron then proton transfer (ETPT), or concerted manners. Here, we use the DFT approach (for more details, see Supporting Information) to elucidate the impact of substituent X (X = H, OEt, and CF_3_) on the calculated energy, geometry, and electronic structures of each intermediate involved in the above-mentioned initial steps of the reaction. These calculations show that the addition of a water molecule to complex **1** is exergonic by Δ*G*° = −5.4, −5.5, and −4.6 kcal/mol for complex **1a** (X = H) and its **1b** and **1d** analogs with electron-donating OEt and electron-withdrawing CF_3_ substituents, respectively. In the resulting [**1a–OH**_**2**_], [**1b–OH**_**2**_], and [**1d–OH**_**2**_] aqua complexes (see Figures S31 and S32 of the Supporting Information), the (a) calculated Ru–OH_2_ bond distances are 2.195, 2.198, and 2.193 Å, respectively, and (b) the Ru(II)-coordinated water molecule forms two O_2_SO---H bonds with the SO_3_-unit of the pendant ligand. As could be expected, these H-bonding interactions weaken the O–H bonds of the water molecule, which is manifested in the change of the calculated (unscaled) O–H bond stretches, which are 3450 and 3546 cm^–1^ for [**1a–OH**_**2**_], 3455 and 3550 cm^–1^ for [**1b–OH**_**2**_], and 3444 and 3537 cm^–1^ for [**1d–OH**_**2**_]. The calculated (unscaled) O–H bond stretches are 3800 and 3902 cm^–1^ for free water molecules. For [**1a–OH**_**2**_], IR(ATR) showed two sharp stretches at 3463.3 and 3547.4 cm^–1^, in good agreement (Figure 5).

The first ETPT starts with one-electron oxidation of the Ru(II)-aqua complexes, [**1a–OH**_**2**_], [**1b–OH**_**2**_], and [**1d–OH**_**2**_] by Ce^IV^-species [the calculated Ce(IV/III) reduction energy is 128.8 kcal/mol], which is exergonic by Δ*G*° = −2.7, −4.7, and −0.5 kcal/mol and leads to the Ru(III)-aqua cations (with *S* = 1/2 spin) [**1a–OH**_**2**_]**^+^**, [**1b–OH**_**2**_]**^+^**, and [**1d–OH**_**2**_]**^+^**, respectively. In the next step, these Ru(III)-aqua cations release one proton (to water solvent: the calculated proton hydration energy is 265.0 kcal/mol), which needs Δ*G*° = 11.2, 12.0, and 9.7 kcal/mol free energy, respectively. As expected, the results of the first ETPT processes are Ru(III)-hydroxyl complexes [**1a–OH**], **[1b–OH**], and **[1d–OH**], respectively. In these complexes, the calculated Ru–O^w^ bonds are significantly (by 0.25–0.26 Å) contracted, and the Ru–N bonds are elongated by ca. 0.03–0.04 compared to the corresponding aqua complexes. We also found that in [**1a–OH**], [**1b–OH**], and [**1d–OH**], the OH-ligand remains H-bonded to the SO_3_-unit, and their unpaired alpha-spin is delocalized over the Ru center and OH-ligand as 0.66–0.65 and 0.32–0.33 |e|, respectively.

Similarly, the second stepwise ETPT requires Δ*G*° = 0.7, – 1.0, and 2.0 kcal/mol free energy for the ET in [**1a–OH**], [**1b–OH**], and [**1d–OH**], respectively, and Δ*G*° = 9.1, 9.7, and 8.3 kcal/mol free energy for the PT from the previously generated [**1a–OH**]**^+^**, **[1b–OH**]**^+^**, and **[1d–OH**]**^+^** cation complexes, respectively. As can be anticipated, the products of the two consequent ETPT processes are the complexes [**1a-O**], [**1b-O**], and [**1d-O**], respectively. The ground electronic states of these product complexes are triplet states with 0.90–0.88 |e| and 1.05–1.08 |e| unpaired spins on their Ru and O-centers, respectively. Thus, these product complexes at their triplet ground electron states are the Ru(III)-oxyl species. In contrast, in their excited diamagnetic singlet states, the complexes [**1a-O·**], **[1b-O·**], and **[1d-O·**] are the Ru(IV)-oxo species with the Ru=O double bond. The presented calculations show that the diamagnetic states of [**1a=O**], [**1b=O**], and [**1d=O**] lie at higher Δ*G*° = 23.3, 23.4, and 21.1 kcal/mol than their triplet Ru(III)–oxyl states. Surprisingly, the calculated Ru–O bond distances in Ru(IV)-oxo complexes are only slightly (by 0.013–0.017 Å) shorter than those in their triplet Ru(III)-Oxyl states. In the ground state Ru(III)-Oxyl complexes [**1a-O·**], [**1b-O·**], and [**1d-O·**], the calculated Ru–O bond distances are 1.789, 1.788, and 1.790 Å, and the Ru–O vibrational frequencies are 818.0, 823.6, and 813.6 cm^–1^, respectively.

**Figure 6:**
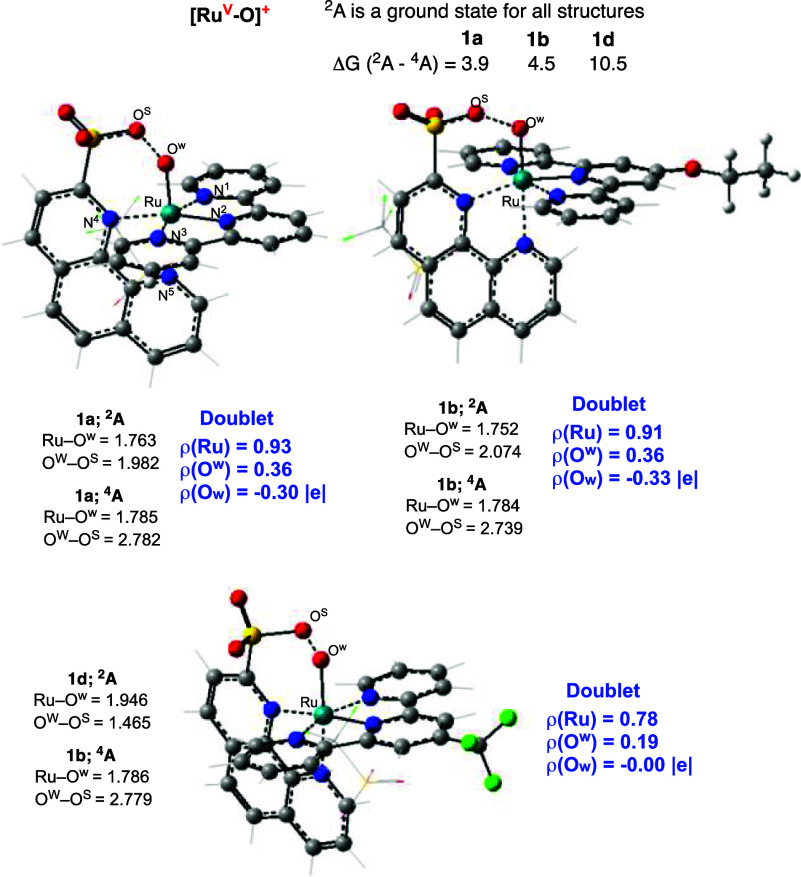
Calculated important geometry and electronic structure parameters of the **[Ru(V)-O]**^**+**^ intermediates for species **1a**, **1b,** and **1d** (see also the available Supporting Information).

Since the one-electron oxidized form of the triplet Ru(III)–oxyl or/and Ru(IV)-oxo species, i.e., the **[Ru(V)-O]**^**+**^ intermediate (see Figure 6 and Supporting Information), is proposed^12,23,27,28,34^ to be one of the possible prereaction complexes for the following O–O coupling, herein we also have analyzed the geometry and electronic properties of **[Ru(V)-O]**^**+**^ intermediates for species **1a**, **1b,** and **1d**. Briefly, the presented DFT calculations show that the **[Ru(V)-O]**^**+**^ intermediates of **1a**, **1b,** and **1d** have doublet ground states. Their quartet high-spin states are 3.9, 4.5, and 10.5 kcal/mol higher in free energy. In doublet state **[Ru(V)-O]**^**+**^ intermediates, Ru-center has 0.93, 0.91, and 0.78 |e| unpaired spins, respectively, while O^W^ (oxo) atom and one of three oxo groups of SO_3_^–^ pendant ligand, labeled as O^S^ in Figure 6, are bearing of 0.36 and 0.30, and 0.36 and 0.33|e| alpha and beta unpaired spins for **1a** and **1b**, respectively. In the doublet **[Ru(V)-O]**^**+**^ intermediate of **1d**, the O^W^ (oxo)-center has 0.19 |e| unpaired spin, while the O^S^-center has no unpaired spin. The presented spin densities of **[Ru(V)-O]**^**+**^ intermediates of **1a**, **1b,** and **1d** are consistent with the calculated critical geometry parameters of these species. In doublet **[Ru(V)-O]**^**+**^ intermediate of **1a** and **1b**, the calculated Ru–O and O^W^–O^S^ bond distances are 1.763 and 1.752, and 1.982 and 2.074 Å, respectively. In contrast, in the doublet **[Ru(V)-O]**^**+**^ intermediate of **1d**, the calculated Ru–O distance is elongated to 1.946 Å, while the O^W^–O^S^ bond distance is shortened to 1.465 Å. Thus, the formal **[Ru(V)-O]**^**+**^ intermediate of **1d** is a **[Ru(III)-peroxo]**^**+**^ complex. The presented geometry parameters of the doublet **[Ru(V)-O]**^**+**^ intermediates of **1a**, **1b**, and **1d,** in agreement with previous findings^27,28^ clearly indicate the importance of the SO_3_^–^ pendant ligand in the O–O coupling process.

To summarize, the presented calculations show that two initial sequential ETPTs, in the presence of CAN, transform the Ru(II)-aqua complexes [**1a–OH**_**2**_], [**1b–OH**_**2**_], and [**1d–OH**_**2**_] into the associated Ru(III)-oxyl complexes [**1a-O·**], [**1b-O·**], and [**1d-O·**]. The overall Δ*G*° values for the [**1-OH**_**2**_] → [**1-O·**] transformations are +7.5, + 5.4, and +10.3 kcal/mol for R = H, OEt, and CF_3_, respectively. The one-electron oxidation of triplet Ru(III)-oxyl [**1-O·**] complex to form the formal **[Ru**^**V**^**–O]**^**+**^ intermediate, that previously was proposed^12,23,27,28,33^ to be species that promote the oxidation of water, i.e., the [**1-O·**] → **[Ru**^**V**^**–O]**^**+**^ transformation, requires 12.9, 12.4, and 7.1 kcal/mol free energy. The presented geometry analyses of **1a**, **1b**, and **1d,** in agreement with previous findings,^27,28,^ clearly indicate the importance of the SO_3_^–^ pendant ligand in the following O–O formation. The role of the SO_3_^–^ pendant ligand is more pronounced in **1d** with *para*-CF_3_-*terpy*, than in **1a** and **1b** with the unsubstituted- and *para*-OEt-*terpy* ligands. Noteworthy, the formal **[Ru(V)-O]**^**+**^ intermediate of **1d** is a **[Ru(III)-peroxo]**^**+**^ complex.

However, the O–O formation step of the studied water oxidation reactions could be very complex^12,23,27,28,33,34^ may proceed very different pathways under the electrochemical and sacrificial oxidant conditions, by involving multiple pathways such as (a) direct bimetallic, i.e. [Ru]O–O[Ru], and (b) heterometallic,^32,34^ i.e. [Ru]O–O[Ce], O–O coupling pathway, (c) water nucleophilic attack (WNA) directly to the oxo-center of Ru^V^–O, and (d) oxide relay mechanism that starts by formation of the Ru(III)-peroxo intermediate via the coupling of oxygen of pendant group with the oxo-center of the Ru^V^–O core, and proceeds by attack of the second water molecule to a heteroatom of the pendant group. While some of these mechanistic scenarios were studied in detail by Mandal and co-workers,^23,28,34^ Ahlquist and co-workers,^27^ and Concepcion and co-workers,^33^ the elucidation of roles of various pendant groups, *para*-substitutions of the *terpy* ligand, pH, as well as the used electrochemical and sacrificial oxidant conditions still need more comprehensive both experimental and computational studies.

## Conclusions

Here, we extended our library of previously reported Ru(4′-X-terpyridine)(phenanthroline-SO_3_)(OTf)(X = H, **1a**) WOC with pendant sulfonate–that showed high water oxidation activity under both sacrificial oxidant Ce(IV) and electrocatalytic conditions, and in both acidic and neutral media by the synthesis of its **1b**–**1d** derivatives (with X = OEt, NO_2_, and CF_3_ substituents, respectively). It is shown that the functionalization of the terpyridine ligand with an electron-donating group (i.e., in **1b**) shifted the Ru (II/III) redox potentials to less positive. For this catalyst, full conversion of the sacrificial oxidant Ce^IV^ was recorded at 0.4 h with a TON of 2010, a TOF of 7.6 s^–1^, and an initial rate of 2.07 μmol O_2_ s^–1^, which is 30-fold faster than that for **1a** at [cat]_0_ = 20 μM. We attribute part of the 30× improvement observed in sacrificial oxidant conditions to the combination of an electron-rich terpyridine scaffold that lowers the oxidation potential of the metal center and the decrease in the Lewis basicity of the ligand due to the interaction with the sulfonate group. Electrocatalysis by **1b** was three times faster than that for **1a** at close to the same potential. Extensive computations have identified differences in the initial PCET steps of the water oxidation by catalysts **1a**, **1b**, and **1d**, and demonstrated the increased probability of the O_2_ formation via the oxide relay mechanism in the order **1b** < **1a** < **1d**.

## Supporting Information Available

Experimental and computational details and X-ray crystal data for **1b** and **1c** (PDF)

## Dedication

This paper is dedicated to the memory of our friend, mentor and coauthor, Prof. Douglas B. Grotjahn, who suddenly passed away when this manuscript was almost ready for submission.
