# Supplementary material for: Electron-Donating para-Substituent (X) Enhances the Water Oxidation Activity of the Catalyst Ru(4′-X-terpyridine)(phenanthroline-SO3)+
Source: Inorg Chem. 2025 Feb 7;64(7):3188–95. doi: 10.1021/acs.inorgchem.4c04124 (PMC11863372; doi:10.1021/acs.inorgchem.4c04124)
Supplement: Supplementary file 1 — ic4c04124_si_001.pdf [file ic4c04124_si_001.pdf]

Supplementary Information

# Electron Donating *para*-Substituent (X) Enhances the Water Oxidation Activity of the Catalyst Ru(4'-X-terpyridine)(phenanthroline-$SO_3$)$^+$

Miguel A. Ibañez,[a] Colton J. Breyer,[a] Milan Gembicky,[c] Zinnun F. Malikov,[d] Djamaladdin G. Musaev,*[,b] and Douglas B. Grotjahn*[,a]

[a] Department of Biochemistry and Chemistry, San Diego State University, San Diego, California 92182-1030, United States

[b] Emerson Center for Scientific Computation and Department of Chemistry, Emory University, Atlanta, GA 30322, United States

[c] Department of Biochemistry and Chemistry, University of California- San Diego, San Diego, California 92093, United States

[d] Innovation Academy, 125 Milton Ave, Alpharetta, GA 30009, United States

dmusaev@emory.edu
Prof. Douglas B. Grotjahn (deceased)

Table of Contents

**I. General Information** ........ S2
*a. General experimental* ........ S2
*b. Electrochemistry* ........ S2
*c. Sacrificial oxidant testing* ........ S2
**II. Synthesis** ........ S2
*a. Ligand synthesis* ........ S2
*b. Catalyst synthesis* ........ S4
**III. Sacrificial oxidant testing** ........ S6
**IV. Electrochemical measurements** ........ S12
**V. Analyzing water oxidation electrocatalysts in the absence of limiting current.** S16
**VI. Spectral data for starting materials and catalysts** ........ S20
*a.* $^{1}$HNMR experimental data ........ S20
**VII. Crystal data and structure refinement for 1b and 1c** ........ S25
**VIII. Computational details** ........ S26
IX. **Cartesian coordinates of the calculated structures** ........ S29

## I. General Information

### *a. General experimental*

Reactions were done under $N_2$ atmosphere using Schlenk lines unless otherwise noted. Solvents used for synthesis and experimental work up were purchased from Fisher Chemicals or Sigma-Aldrich. NMR experiments were done at 'room temperature' using commercial deuterated solvents purchased from Cambridge Isotope Labs.

### *b. Electrochemistry*

CHI 730E electrochemical analyzer potentiostat was used to analyze and perform cyclic voltammetry experiments. The single-compartment cell was equipped with a 3mm boron-doped diamond (BDD) working electrode, Ag/AgCl was used as a counter electrode, and Platinum wire was used as the reference electrode. The working electrode was polished before experiments using 0.3 micron and 0.05 micron polishing compound. Applied voltage and IR compensation were unnecessary since experiments were done in aqueous media. The experiments were done at 'room temperature' using 18 MΩ ultrapure water degassed with argon before experiments and headspace blanketed with argon gas during experiments. pH of solutions was adjusted using phosphate buffer ($H_2PO_4^{2-}/HPO_4^{2-}$), ionic strength was adjusted using $KNO_3$, and the solution was made acidic using $HNO_3$ for acidic pH experiments. Each catalyst was tested in triplicate to ensure reproducibility at scan rates ranging from 0.01 V/s to 20 V/s, scanning positive from 0 (V), 1.7 (V), -1 (V), to 0 (V).

### c. *Sacrificial Oxidant Testing*

A custom-built 10 mL pressure cell was used to measure the water oxidation capability of the catalysts in this study. The pressure cell was calibrated by liquid volumetric measurements to ensure the accuracy and reliability of pressure measurements. The cell is equipped with a pressure transducer, thermal couple, and injection port to incorporate sacrificial oxidant and catalyst solution. The temperature of the cell was maintained using a Lauda bath. All process controls were monitored using LabView software. The oven-dried cell was cooled to room temperature, to which 10 mL of a 0.2 M cerium (IV) ammonium nitrate (CAN) solution was added. CAN solution was made by dissolving the appropriate amount of cerium in 0.1 M nitric acid. Once the cell pressure and temperature stabilized, the catalyst solution was injected through the injection port of the pressure cell. A catalyst solution was made by dissolving the appropriate amount of $[catalyst]_0$ in reagent-grade acetonitrile. The average injection volume for 20 μM catalyst concentration was 50 μL Experiments were monitored until pressure increase was no longer detected. Complete catalyst consumption was determined by calculating and observing the theoretical amount of pressure change due to the formation of $O_2$ and by adding excess catalyst solution at the end of the experiment. Experiments were performed in triplicate to ensure reliability.

## II. Synthesis

### *a. Ligand Synthesis*

**Synthesis of L0**

Synthesis of 4'-chloro-2,2':6,2"-terpyridine: Procedure was followed using the steps outlined by Constable[9]. 2,6-bis(pyridyl-4-pyridone (2.92 g, 0.01171 mol) and $PCl_5$ (6 g, excess) solids were weighed and added to an oven-dried 250mL round bottom flask. To the dry solids, $POCl_3$ (80 mL) was measured using a graduated cylinder and added all at once to yield a gray mixture that was heated to reflux for 24 h. The mixture was allowed to cool to room temperature, and volatiles were removed under vacuum, with a receiving flask cooled in liquid nitrogen. The resulting solid was then dissolved by adding DI water (50mL), and the resulting mixture was made basic (pH = 7) by adding 1M NaOH. The product was extracted using DCM (3 x 20 mL). The organic fractions were combined, dried over $MgSO_4$, filtered using a coarse frit, and the filtrate was concentrated by rotary evaporation. The obtained solid was washed with diethyl ether in a frit and recrystallized from hot ethanol. The product was obtained as off-white crystals (1.90 g, 61% yield). NMR characterization was done using $CDCl_3$ and matched literature values.

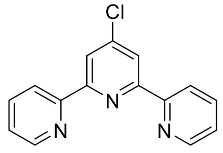

L0

**Synthesis of L1**

The procedure was followed using the steps outlined by Yagi[5]: In an N2-filled glovebox, a 25% w/w solution of sodium ethoxide was made by dissolving sodium ethoxide (12.5 g) in ethanol and adding ethanol until a mass of 50 g was reached. In the glovebox, a 250 mL oven-dried round bottom flask was charged with 4'-chloro-2,2':6,2"-terpyridine (350 mg, 1.307 mmol), and ethanol (20 mL) was added to make a suspension. To the mixture, a portion of the prepared sodium ethoxide solution (30 mL) was added to the suspension all at once. The flask was capped with a septum, and the reaction mixture, warm to the touch, was brought out of the glovebox and heated to reflux under $N_2$ flow. After 1 d, the mixture was cooled to room temperature and filtered through a coarse frit. The tan-brown filtrate was added to an Erlenmeyer flask with DI water (~ 200 mL), giving an off-white precipitate. The resulting white precipitate was separated from the solution using a medium frit, collected, and dissolved in DCM. The DCM fraction was then dried over $MgSO_4$, and the liquid was collected by vacuum filtration. The organic fraction was concentrated by rotary evaporation. The product was obtained as an off-white solid (219 mg, 60% yield). NMR characterization was done using $CDCl_3$ and was consistent with literature values.

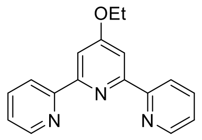

L1

**Synthesis of L2**

The procedure was followed using the steps outlined in patent[11]. In an $N_2$-filled glovebox, a pressure vessel (250mL) was charged with 2,6, -dibromo-4-nitro pyridine (1.5 g, 0.5321 mmol), tributyl (pyridine-2-yl) stannane (3.917 g, 1.0642 mmol), and palladium tetrakis (122.9 mg, 0.01064 mmol). Dry toluene (150 mL) was added to the mixture of dry solids and removed from the glove box. The mixture was heated to 120 °C for 2 d to yield a transparent dark brown solution. The crude mixture was allowed to cool to room temperature, quenched with DI water (~10 mL), and extracted into toluene (3 X 20mL). The organic fraction was retained and dried over $MgSO_4$. The filtrate retained was concentrated to a brown solid by rotary evaporation. The crude solid material was purified by column chromatography (20% EtOAc/ 80% Hexanes). The obtained solid was recrystallized from hot dichloromethane, yielding a light brown crystalline solid. (728 mg, 51% yield). NMR experiments were done in $CDCl_3$ and matched literature values.

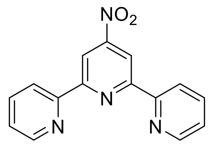

L2

**Synthesis of L3**

The procedure was followed using the steps outlined by Pangu[10]. In an $N_2$-filled glovebox, 2-pyridyl tributyl stannane (0.62 mL, 1.1938 mmol) and 2,6-dichloro-4-(trifluoromethyl) pyridine (0.137 mL, 0.9376 mmol) were measured out using a syringe, added to a 10 mL vile, and dissolved in dry toluene. In the glove box, an oven-dried round bottom flask (250mL) was charged with palladium tetrakis triphenylphosphine (11 mg, 9.576 µmol) and dissolved in dry toluene (100 mL). To the palladium catalyst mixture, the starting material mixture containing 2,6-dichloro-4-(trifluoromethyl) pyridine and 2-pyridyl tributyl stannane was added using a glass Pasteur pipette. The reaction vessel was brought out of the glove box and heated to reflux under $N_2$. After 1 d, NMR suggested the single substituted product, so another equivalence of palladium catalysts was added under an inert atmosphere to complete the reaction. After 2 d, the bipyridine concentration decreased tremendously by NMR, and the brown-colored reaction mixture was allowed to cool to room temperature. Ethyl acetate (15 mL) was added to the mixture, quenched with saturated ammonium chloride (5 mL), and made basic using 1M NaOH until pH = 7 was achieved. The crude product was extracted using ethyl acetate (2 x 20 mL) and collected. The organic layer was dried using $MgSO_4$ and recollected by vacuum filtration using a coarse frit. The organic phase was concentrated by rotary evaporation and then purified by chromatography (20% EtoAc/ 80% hexanes). An off-white solid was recovered after concentrating fractions by rotary evaporation (103 mg, 36% yield). NMR characterization was done using $CDCl_3$ and was consistent with literature values.

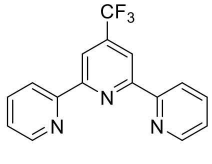

L3

b. *Catalyst Synthesis*

**A) Synthesis of C2**

In an $N_2$-filled glovebox, $RuCl_2(DMSO)_4$ (174.4mg, 0.36mmol) and 4'-ethoxy-2,2':6,2"-terpyridine (100mg, 0.36mmol) were added to a pressure vessel and dissolved in 200 proof dry ethanol (50 mL). The mixture was removed from the glovebox and heated to 90 °C for 24 h. The mixture evolved from a yellow-colored suspension to a red-colored solution. The mixture was cooled to room temperature and filtered through a fine frit. The solid retained was washed using a fine frit with cold ethanol (5 mL) and cold DI water (5 mL), followed by multiple washes

of cold diethyl ether (3 x 10 mL). The dark red solid was collected by vacuum frit filtration and dried using $P_4O_{10}$ under an oil pump vacuum overnight. (173mg, 91% yield).

**B) Synthesis of 1b**

In an $N_2$-filled glovebox, an oven-dried round bottom flask was charged with [(4'-ethoxyterpy)Ru(DMSO)($Cl_2$)] (0.308 g, 0.5840 mmol), AgOTf (0.3g, 1.1679 mmol), $H_2O$ (2 mL), and suspended in 200 proof ethanol (50 mL). The suspension was stirred for 1 h, then filtered by vacuum filtration using a fine frit. The bright red-orange filtrate was transferred to an oven-dried pressure vessel to which 1,10-phenanthrolinium-2-sulfonate dihydrate (0.1514 g, 0.5840 mmol), NaOH (0.58 mL, 0.5840 mmol), and 200 proof ethanol (50 mL) was added in the glovebox. The mixture was brought from the glove box and heated in an oil bath at 90 °C. After 1 d, the reaction mixture was cooled to room temperature, then chilled for 4 h at 32 °C. The cooled mixture was filtered by vacuum filtration using a fine frit. The deep red solid was washed with cold ethanol (20 mL) and diethyl ether (20 mL). Using a new filter Erlenmeyer flask, excess acetone was added (~400 mL) to the solid in the frit until most of the material dissolved. The filtrate was transferred to a round bottom flask and dried by rotary evaporation. The solid was dried using $P_4O_{10}$ under an oil pump vacuum for 2 d to yield a red solid. (0.2260 mg, 49% yield). Elem. Anal. Calculated for $C_{30}H_{22}F_3N_5O_7RuS_2$ (786.72): C, 45.80; H, 2.82; N, 8.90; S, 8.15. Found: C, 45.00; H, 2.91; N, 8.87; S, 7.94. Anal. Calculated for $C_{30}H_{22}F_3N_5O_7RuS_2$ + $H_2O$ (804.73): C, 44.78; H, 3.01; N, 8.70; S, 7.97.

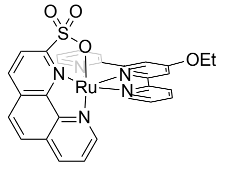


1b

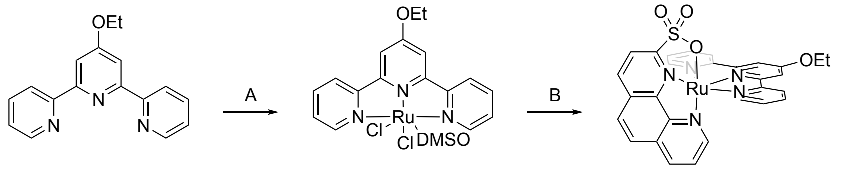


L1 C2 **1b**

**C) Synthesis of C3**

In an $N_2$-filled glovebox, $RuCl_2(DMSO)_4$ (100mg, 0.2064mmol) and 4'-nitro-2,2':6,2"-terpyridine (57.4mg, 0.2064mmol) were added to a pressure vessel and dissolved in 200 proof dry ethanol (50 mL). The mixture was removed from the glove box and heated to 90 °C for 24 h. The mixture evolved from a yellow-colored mixture to a dark purple solution. The solution was cooled to room temperature and filtered through a fine frit. The solid retained was washed in a fine frit with cold ethanol (5 mL) and cold DI water (5 mL), followed by multiple washes of cold diethyl ether (3 x 10 mL). The dark purple solid was collected by vacuum frit filtration and dried using $P_4O_{10}$ under an oil pump vacuum overnight. (66.1mg, 66% yield). Elem. Anal. Calculated for $C_{17}H_{16}Cl_2N_4O_3RuS$ (528.37): C, 38.64; H, 3.05; N, 10.60; O, 9.08; S, 6.07. Found: C, 38.26; H, 3.06; N, 10.53; S, 6.18.

D) **Synthesis of 1c**

In an $N_2$-filled glovebox, an oven-dried round bottom flask was charged with [(4'-nitroterpy)Ru(DMSO)($Cl_2$)] (0.5 g, 0.9463 mmol), AgOTf (0.4863g, 1.8926 mmol), $H_2O$ (2 mL), and suspended in 200 proof ethanol (50 mL). The suspension was stirred for 1 h, then filtered by vacuum filtration using a fine frit. The red-purple colored filtrate was transferred to an oven-dried pressure vessel to which 1,10-phenanthrolinium-2-sulfonate dihydrate (0.2453 g, 0.9463 mmol), NaOH (0.94 mL, 0.9463 mmol), and 200 proof ethanol (50 mL) was added. The mixture was brought from the glove box and heated in an oil bath at 90 °C. After 1 d, the reaction mixture was cooled to room temperature, then chilled for 4 h at 32 °C. The cooled mixture was filtered by vacuum filtration using a fine frit. The deep red-purple solid was washed with ethanol (20 mL) and diethyl ether (20 mL). Using a new filter Erlenmeyer flask, acetone was added to the solid in the frit until most of the material dissolved (300 mL). The filtrate was transferred to a round bottom flask and dried by rotary evaporation. The solid was dried using $P_4O_{10}$ under an oil pump vacuum for 2 d to yield a deep purple solid. (0.5350 mg, 72% yield). Elem. Anal. Calculated for $C_{28}H_{17}F_3N_6O_8RuS_2$ (787.66): C, 42.70; H, 2.18; N, 10.67; S, 8.14. Found: C, 43.12; H, 2.57; N, 10.16; S, 7.98. Anal. Calculated for $C_{28}H_{17}F_3N_6O_8RuS_2$ + $CH_3CH_2OH$ (833.73): C, 43.22; H, 2.78; N, 10.08; S, 7.69.

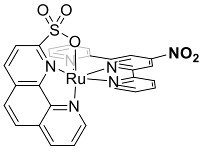


1c

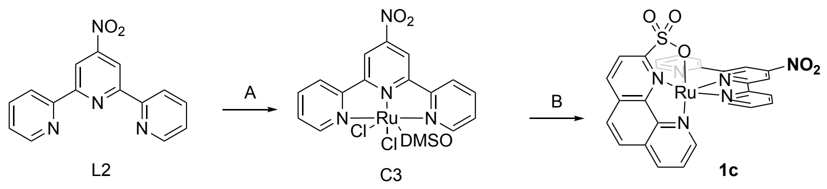


**E) Synthesis of C4**

In an $N_2$-filled glovebox, $RuCl_2(DMSO)_4$ (100mg, 0.2064mmol) and 4'-trifluoromethyl-2,2':6,2"-terpyridine (62.1mg, 0.2064mmol) were added to a pressure vessel and dissolved in 200 proof dry ethanol (50 mL). The mixture was removed from the glove box and heated to 90 °C for 24 h. The mixture evolved from a yellow-colored solution to a dark red/purple mixture. The mixture was cooled to room temperature and filtered through a fine frit. The solid retained was washed using a fine frit with cold ethanol (5 mL) and cold DI water (5 mL), followed by multiple washes of cold diethyl ether (3 x 10 mL). The red solid was collected by vacuum frit filtration and dried using $P_4O_{10}$ under an oil pump vacuum overnight. (93.6mg, 82% yield).

**F) Synthesis of 1d**

In an $N_2$-filled glovebox, an oven-dried round bottom flask was charged with [(4'-$CF_3$terpy) Ru(DMSO)($Cl_2$)] (0.025 g, 0.04534 mmol), AgOTf (0.02320g, 0.09068 mmol), H2O (0.5 mL), and suspended in 200 proof ethanol (25 mL). The suspension was stirred for 1 h, then filtered by vacuum filtration using a fine frit. The red-colored filtrate was transferred to an oven-dried pressure vessel to which 1,10-phenanthrolinium-2-sulfonate dihydrate (0.01175 g, 0.04534 mmol), NaOH (0.45 µL), and 200 proof ethanol (25 mL) were added. The mixture was brought from the glove box and heated in an oil bath at 90 °C. After 1 d, the reaction mixture was allowed to cool to room temperature, then chilled for 4 h at 32 °C. The cooled mixture was filtered by vacuum filtration using a fine frit. The deep red-purple solid was washed with ethanol (10 mL) and diethyl ether (10 mL). Using a new filter Erlenmeyer flask, acetone was added to the solid in the frit until most of the material dissolved (120mL). The filtrate was transferred to a round bottom flask and dried by rotary evaporation. The solid was dried using $P_4O_{10}$ under an oil pump vacuum for 2 d to yield a red solid. (0.03676 g, 69% yield). Elem. Anal. Calculated for $C_{29}H_{17}F_6N_5O_6RuS_2$ (810.66): C, 42.97; H, 2.11; N, 8.64; S, 7.91. Found: C, 43.48; H, 2.41; N, 8.19; S, 7.68. Anal. Calculated for $C_{29}H_{17}F_6N_5O_6RuS_2$ + $CH_3CH_2OH$ (856.73): C, 43.46; H, 2.71; N, 8.17; S, 7.48.

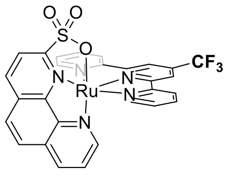

**1d**

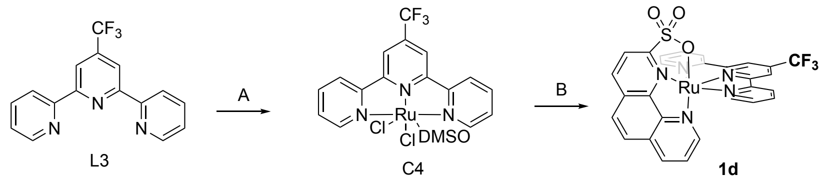

### III. Sacrificial Oxidant Testing

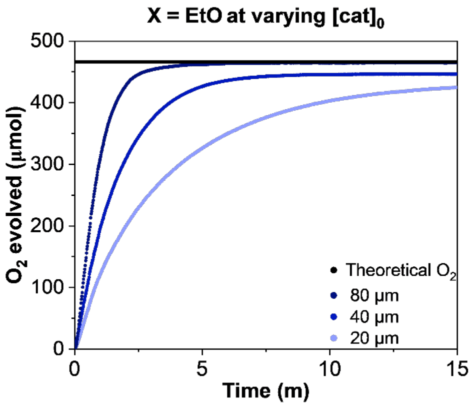


**Figure S1**: Sacrificial oxidant testing of **1b** [Ru(EtO-tda)(phen-$SO_3$)OTf], in [$Ce^{IV}$] = 0.2 M solution and varying $[cat]_0$. The graph shows the rate of oxygen evolution with respect to time. Reaction volume = 10 mL, T = 30 °C.

| Catalyst (µM) | % yield ± 2.4 | TON | Initial rate (µmol $O_2$ /sec) |
|---|---|---|---|
| 5 | 89 | 9200 ( 4 h ) | 0.46 |
| 10 | 97 | 4490 (1.5 h ) | 1.02 |
| 20 | 94 | 2010 ( 0.4 h ) | 2.07 |
| 40 | 97 | 1180 ( 0.3 h ) | 3.40 |
| 80 | 100 | 600 ( 0.2 h ) | 5.74 |

**Table S1**: Production of $O_2$ for **1b** was determined using a sacrificial oxidant. Maximum theoretical moles of $O_2$ produced were calculated by the total moles of cerium (IV) ammonium nitrate used in the run. Average standard deviation of sacrificial oxidant consumption was σ = 2.4.

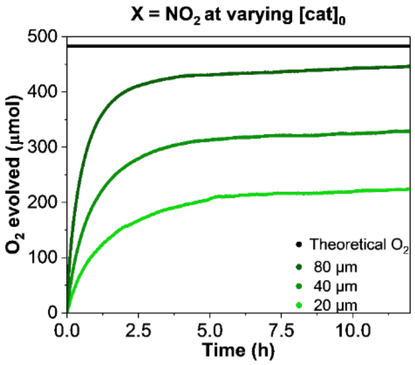


**Figure S2**: Sacrificial oxidant testing of **1c** [Ru($NO_2$-tda)(phen-$SO_3$)OTf] in [$Ce^{IV}$] = 0.2 M solution and varying $[cat]_0$. The graph shows the rate of pressure development in the cell with respect to time. The rate of oxygen evolution stabilizes after ~4-6 h at all $[cat]_0$.

| Catalyst (μM) | % yield ± 1.4 | TON | Initial rate (μmol $O_2$/sec) |
|---|---|---|---|
| 20 | 47 | 1130 ( 12 h ) | 0.095 |
| 40 | 72 | 820 ( 12 h ) | 0.18 |
| 80 | 100 | 540 ( 12 h ) | 0.39 |

**Table S2**: Sacrificial oxidant testing values for **1c**. Average standard deviation of sacrificial oxidant consumption was σ = 1.4.

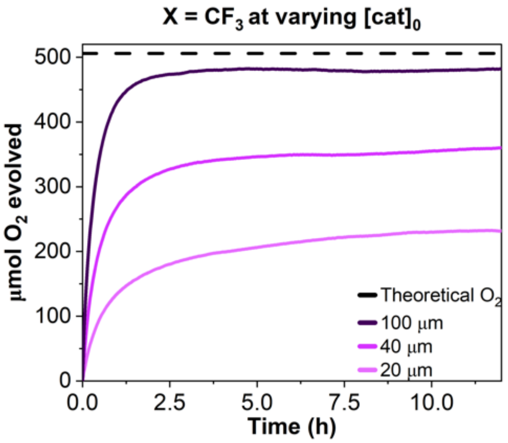


**Figure S3**: Sacrificial oxidant testing of **1d** [Ru($CF_3$-tda)(phen-$SO_3$)OTf] in [$Ce^{IV}$] = 0.2 M solution and varying $[cat]_0$. The graph shows the rate of oxygen evolution in the cell with respect to time.

| Concentration (μM) | % yield ± 2.2 | TON | Initial rate (μmol $O_2$/sec) |
|---|---|---|---|
| 20 | 47 | 1170 (12 h) | 0.096 |
| 40 | 72 | 880 (12 h) | 0.21 |
| 100 | 94 | 530 (12 h) | 0.50 |

**Table S3**: Sacrificial oxidant testing values of **1d**. Average standard deviation of sacrificial oxidant consumption was σ = 2.2.

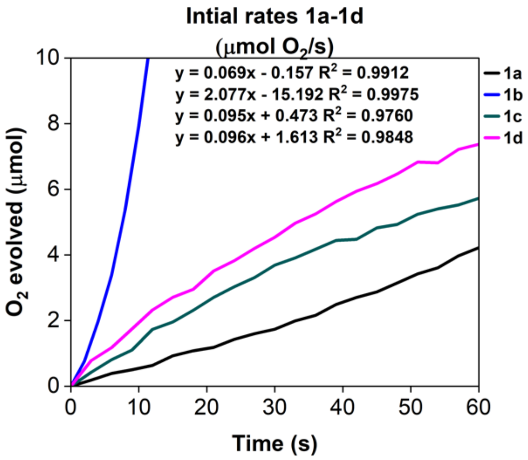


**Figure S4**: Oxygen evolution during the first 60 seconds of the reaction was evaluated for new catalysts functionalized with electron-donor and -withdrawing groups (**1a-1d**). Slopes from equations on graphs correspond d to the initial rate for each catalyst.at $[CAN]_0$ = 0.200 M.

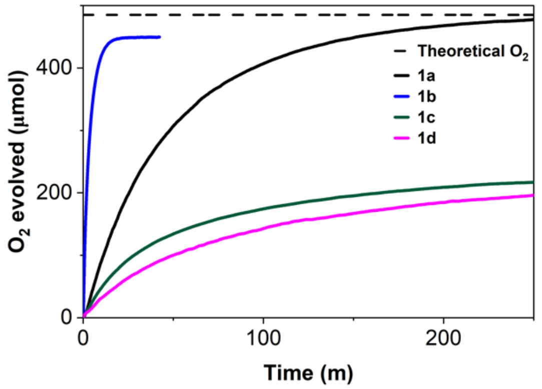


**Figure S5**: $O_2$ evolution of **1a-1b** over the course of ~4 h when $[cat]_0$ = 20 μM. **1b** [Ru(EtO-tpy)(phen-$SO_3$)OTf] consumes all sacrificial oxidant 30 x faster than parent compound **1a,** containing no substituents on terpyridine ligand.Catalysts **1c** and **1d** do not reach theoretical $O_2$ yield at $[cat]_0$ = 20 μM.

| Catalyst (20μM) | Initial rate (μM $O_2$/sec) | TON | % yield | $R^2$ |
|---|---|---|---|---|
| **1a** | 0.069 | 2400 (7 h) | 98±5 | 0.9917 |
| **1b** | **2.07** | **2010 (0.4 h)** | **94±2.4** | **0.9969** |
| **1c** | 0.095 | 1130 (12 h) | 47±1.4 | 0.9780 |
| **1d** | 0.096 | 1170 (12 h) | 47**±2.2** | 0.9844 |

**Table S4**: Initial rates and turnover number from CAN testing of catalysts **1a-1d.**

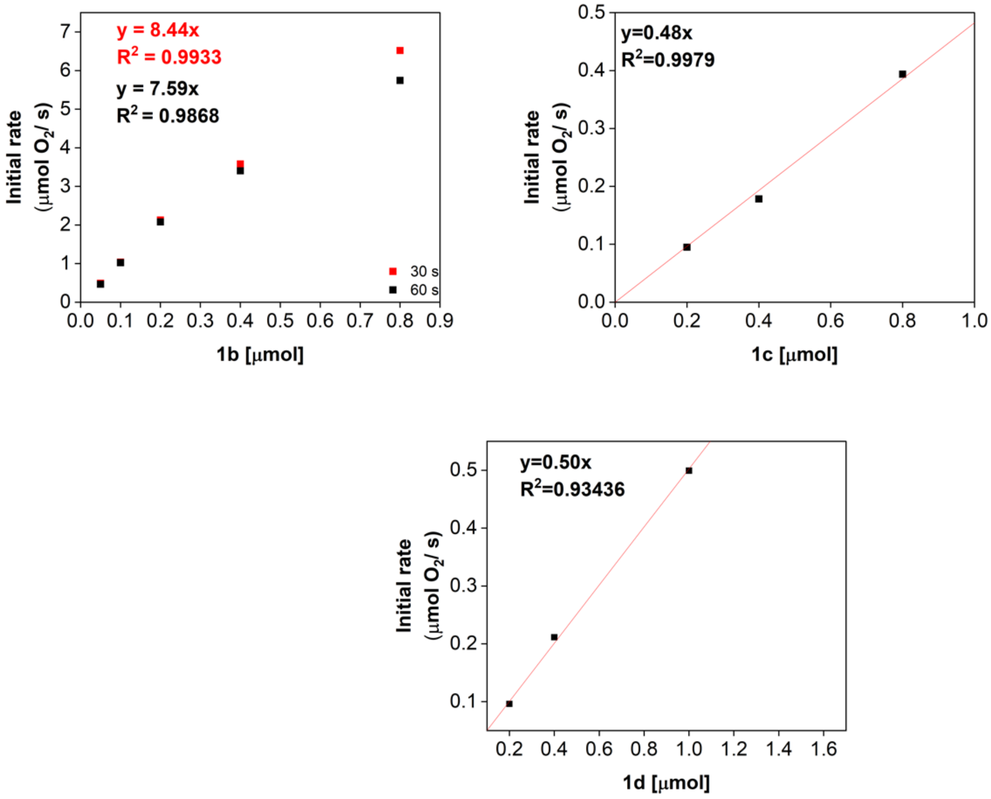


**Figure S6:** Determining TOF 1st order rate constant with respect to catalyst concentration for **1b** at 0.2 M CAN solution. First-order rate with respect to catalyst concentration was observed based on the linear fit of the plots. The slope should be equivalent to the TOF a constant concentration of cerium with units of μmol $O_2$ per second per μmol catalyst.

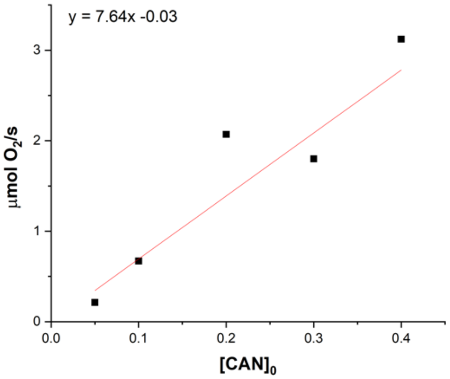


**Figure S7:** Determination of reaction order with respect to sacrificial oxidant [$Ce^{IV}$] with a constant 20 μM concentration of **1b**. An observed linear correlation suggests first order kinetics of the reaction with respect to [$Ce^{IV}$].

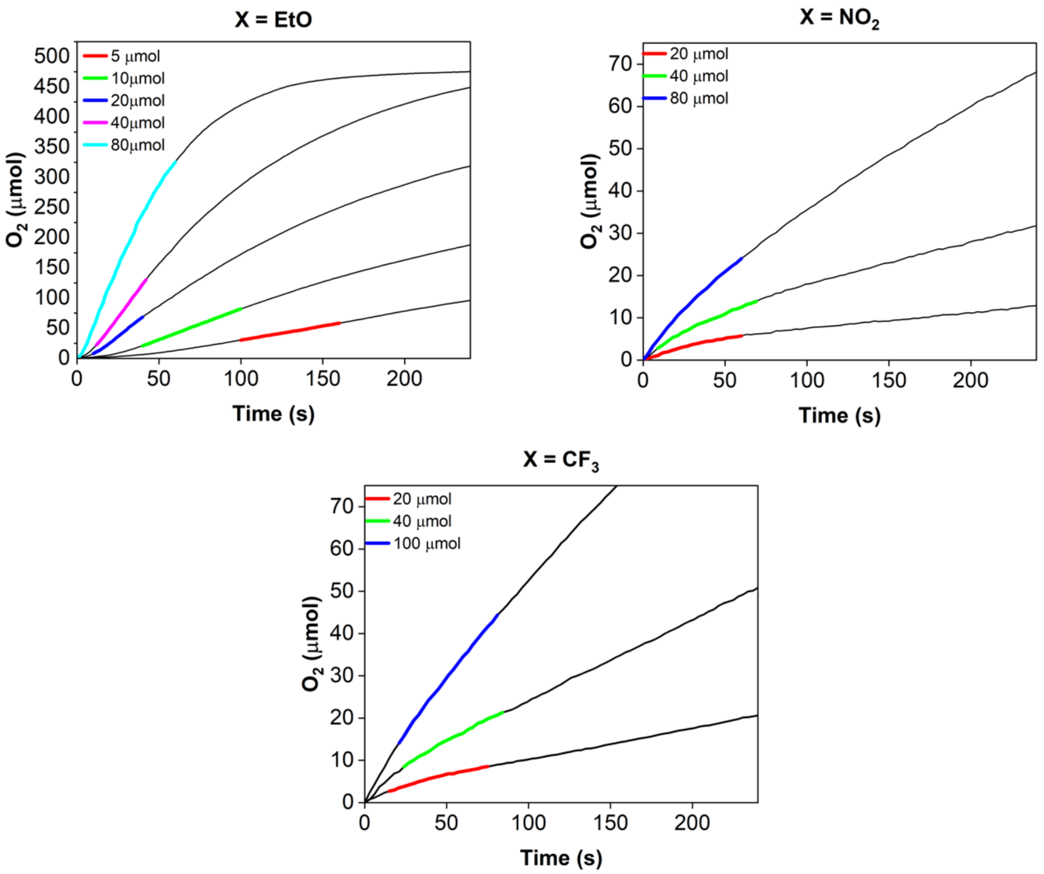


**Figure S8:** Initial rates of catalyst **1b-1d** at varying $[cat]_0$. Linear regression fitted line was used to find the slope for each catalyst at a given concentration. The highlighted region shows the area at which the slope was taken.

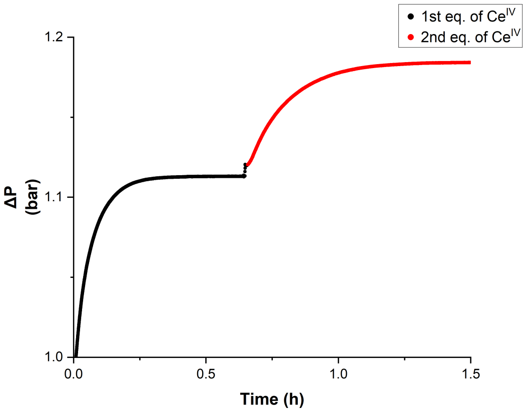


**Figure S9:** Sacrificial oxidant testing using two separate equivalents of $Ce^{IV}$ to demonstrate **1b** remains active after consumption of 1st equivalent sacrificial oxidant when $[cat]_0$= 20μM and $[Ce^{IV}]$ = 0.2M. Black line shows 1st equivalent of $Ce^{IV}$ resulting in 100% conversion followed by the 2nd equivalent of $Ce^{IV}$ illustrated by the red line where 57% of $Ce^{IV}$ is consumed. Pressure generation proportional to the theoretical amount of oxygen produced from the known amount of sacrificial oxidant illustrates full consumption of 1st equivalent of sacrificial oxidant. After a second equivalent of sacrificial oxidant is added (red line), a change of pressure is observed suggesting catalyst is still active and pressure change observed is due to oxygen evolution rather than $CO_2$ gas from catalyst degradation. Additionally, the amount of $CO_2$ produced from a 20μM solution of catalysts would not generate the large amount of pressure observed here.

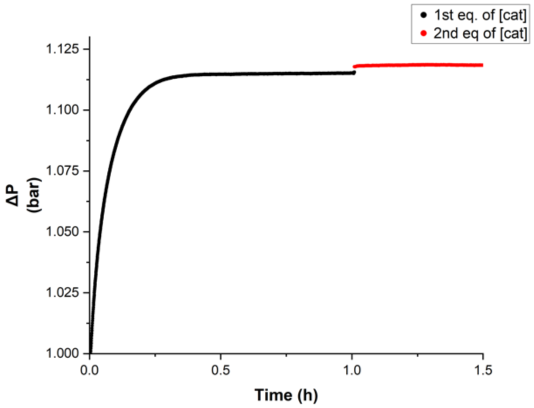


**Figure S10:** Sacrificial oxidant testing of **1b**, $[cat]_0$ = 20μM using $[Ce^{IV}]$ = 0.2M (black line), reaching completion after ~ 0.4h, followed by a $2^{nd}$ equivalent of **1b** (red line). The minute change in pressure of 3.3mbar attributed to sample injection confirms full consumption of $Ce^{IV}$ in the ~0.4h we have reported.

## IV. Electrochemical Measurements

Phosphate buffer ionic strength I = 0.5

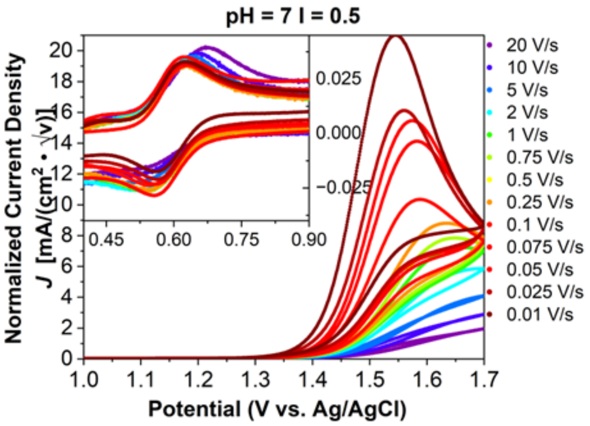


Phosphate buffer ionic strength I = 0.25

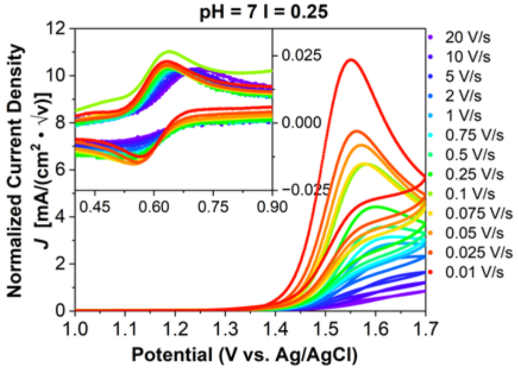


Phosphate buffer ionic strength I = 0.1

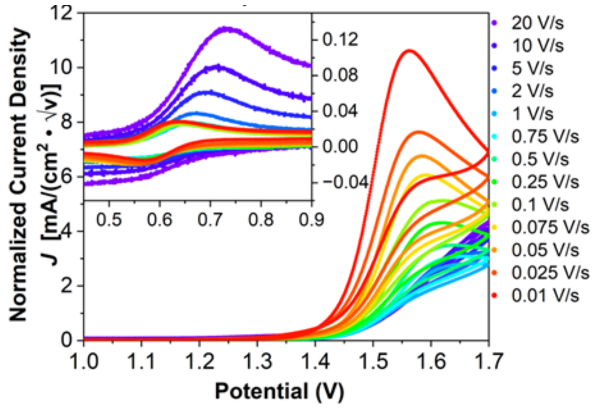


**Figure S11**: Full range of scan rates of **1b** demonstrating scan rate dependence at varying ionic strength of buffer. Conditions: $[cat]_0$ = 50 μM, pH = 7 phosphate buffer ($H_2PO_4^-/HPO_4^{2-}$), and 3mm boron-doped diamond electrode. Overall ionic strength adjusted to I = 0.5 M using $KNO_3$.CVs are normalized to scan rate.

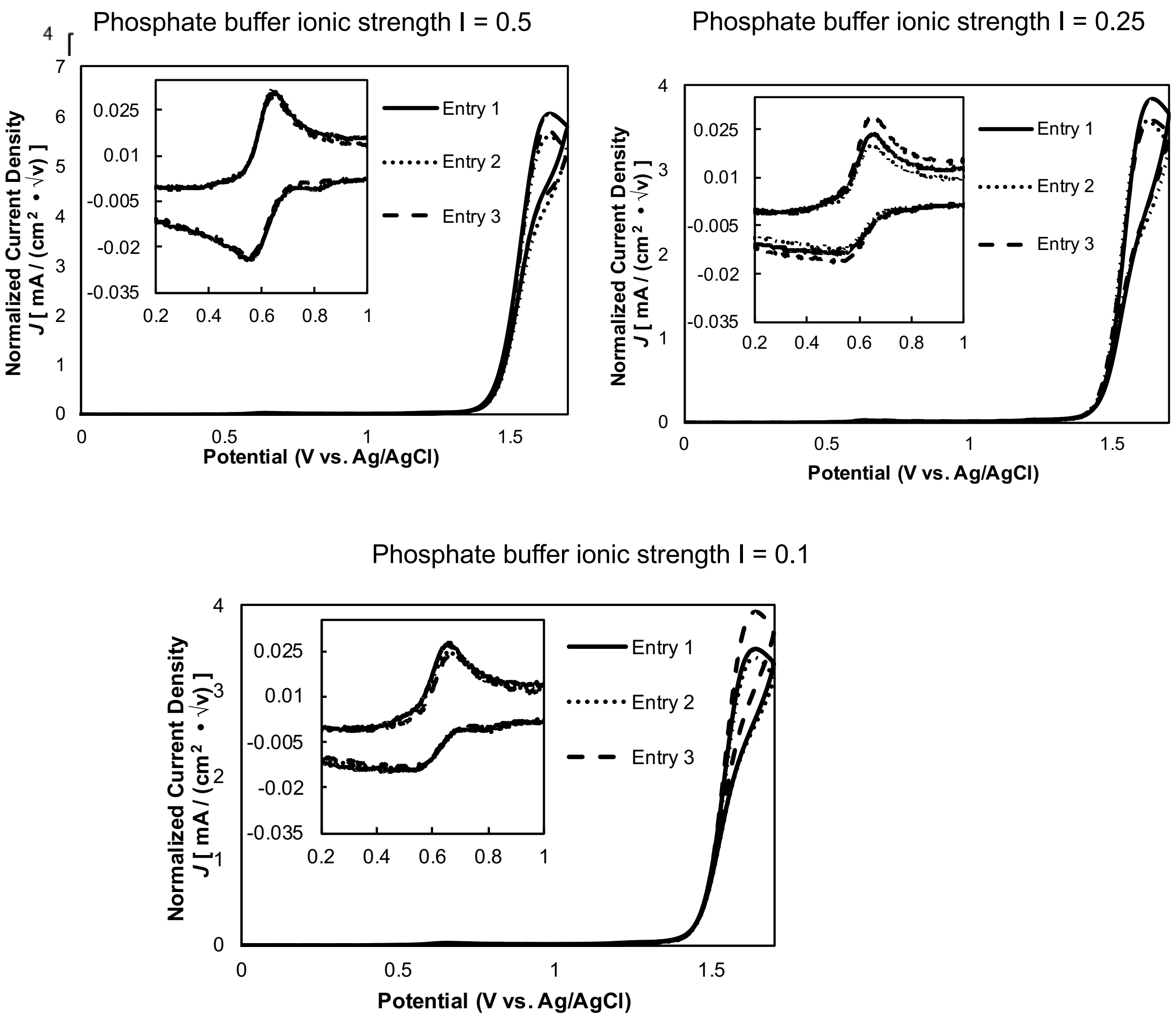


**Figure S12**: Background-subtracted cyclic voltammograms of **1b** in triplicate at varying ionic strength of buffer. Conditions: $[cat]_0$ = 50 μM, SR = 0.5 V/s, pH = 7 ($H_2PO_4^-/HPO_4^{2-}$), I = 0.5, 0.25, 0.1, and 3mm boron-doped diamond electrode). Overall ionic strength adjusted to I = 0.5 M using $KNO_3$. CVs are normalized to scan rate.

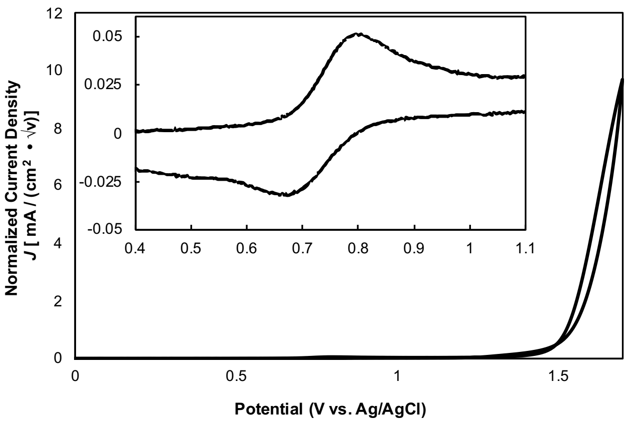


**Figure S13**: Cyclic voltammogram of **1c** [Ru($NO_2$-tpy)(phen-$SO_3$)OTf]. Conditions: $[cat]_0$ = 50 μM, SR = 0.5 V/s, pH = 7 ($H_2PO_4$-/$HPO_4^{2-}$) I = 0.1, and 3mm boron-doped diamond electrode. More positive $E_{1/2}$ (V) was observed compared to ethoxy-substituted terpyridine analog **1b.** Overall ionic strength adjusted to I = 0.5 M using $KNO_3$. CVs are normalized to scan rate.

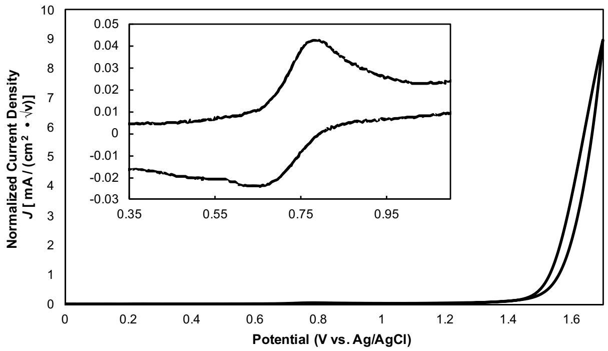


**Figure S14**: Cyclic voltammogram of **1d** [Ru($CF_3$-tpy)(phen-$SO_3$)OTf]. Conditions: $[cat]_0$ = 50 μM, SR = 0.5 V/s, pH = 7 ($H_2PO_4$-/$HPO_4^{2-}$), I = 0.1, and 3mm boron-doped diamond electrode. More positive $E_{1/2}$ (V) was observed compared to ethoxy-substituted terpyridine analog **1b** but less positive than **1c.** Overall ionic strength adjusted to I = 0.5 M using $KNO_3$. CVs are normalized to scan rate.

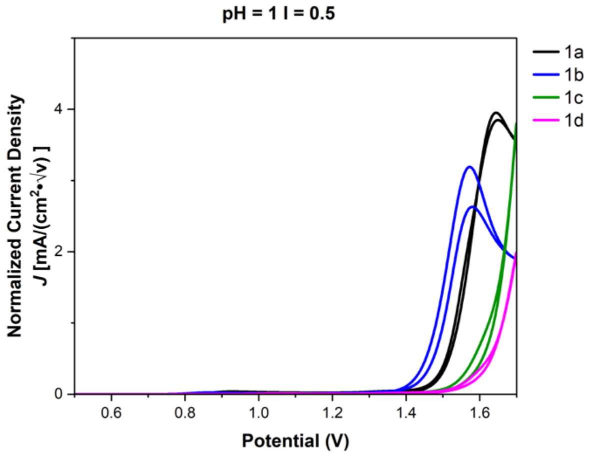


**Figure S15**: Current density of 1a-1d to compare the overpotential of each catalyst as described by Appel (ref). Observed overpotentials (η) are η(1a) = 590 mV, η(1b) = 560 mV, η(1c) = 680 mV, and η(1d) = 690 mV. Precise determination of overpotential is somewhat of a tricky topic when regarding these homogenous water oxidation catalysts. Because water is acting as both the substrate and the solvent, the redox event responsible for catalysis is not obtainable through the limitation of the substrate. Inaccuracies may be present with the determination of overpotential, especially when used for 1c and 1d as both do not show any signs of a purely kinetic, S-shaped, wave.

•

### *V. A method for analyzing water oxidation electrocatalysts in the absence of limiting current.*

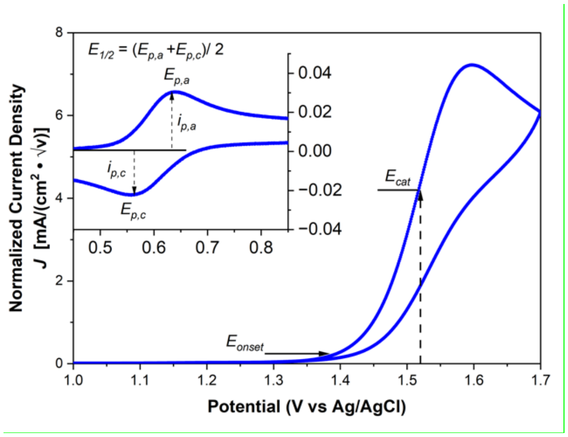


**Figure S16:** Cyclic voltammogram of catalyst **1b** under conditions of pH 7, I=0.5 phosphate buffer, and v = 0.1 V/s. Variables of interest were illustrated on the relevant region of the CV and are defined as follows: $i_{p,a}$ is the peak anodic current, $i_{p,c}$ is the peak cathodic current, $E_{p,a}$ is the potential at peak anodic current, $E_{p,c}$ is the potential at peak cathodic current, $E_{1/2}$ is the half-wave potential of the $Ru^{II/III}$ couple, $E_{onset}$ is the onset potential of catalysis, and $E_{cat}$ is the optimized potential to determine $i_{cat}$ for use in pseudo-limiting current methods.

The following analysis uses work from T. J. Meyer group[16,30] (see also Groves[29]) where a buffer base dependence on catalysis was observed to follow eq. 1

Eq 1 $$k_{cat} = k_{H2O} + k_B[B]$$

Where $k_{H2O}$ is the rate constant of water nucleophilic attack that is unassisted by buffer base B, and kB is the rate constant of the base-assisted reaction. To determine $k_{cat}$ as a function of base, Meyer used limiting-current methods, and determined the value of $E_{cat}$ from the potential that provided the best approximation to limiting current.

Eq 2 $$\frac{i_{lim}}{i_p} = 2.241 \cdot \frac{n_{cat}}{n_p} \cdot \left(\frac{k_{cat} \cdot R \cdot T}{n_p \cdot F \cdot v}\right)^{\frac{1}{2}}$$

Where $i_p$ is the peak current for a simple pre-catalytic redox couple in amps, $n_p$ is the number of electrons transferred during the redox event, $i_{lim}$ is limiting current of catalysis in amps, $n_{cat}$ is the number of electrons transferred in catalysts, , $F$ is Faraday's constant in C/mol, $v$ is the scan rate in V/s, $R$ is the gas constant in J/Kmol, T is the temperature in K and $k_{cat}$ is the forward rate constant of the rate limiting step of catalysis.In theory, limiting current will be obtained upon lowering the scan rate to be slow enough to not outpace the rate of catalysis. However, for complex **1b**, regardless of how low a scan rate was used, the classic S-shaped curve indicating limiting current[18] was never obtained. Therefore, to use existing limiting current methods to determine the catalytic rate constant (see Eq 2), a potential at which catalysis behaves similar to that of limiting current is required. To obtain this potential at which catalytic current was determined, the $R^2$ was determined for two separate plots over a range of potentials. The first plot (Fig. S17) optimizes the linear fit of $k_{cat}$ vs the concentration of buffer base, [B] for five reasonable scan rates (0.025 V/s, 0.05 V/s, 0.075 V/s, 0.1 V/s, 0.25 V/s) as the linearity of this plot should be scan rate independent. The second plot (Fig. S18) optimizes the linear fit of $i_{cat}/i_p$ vs $1/v^{½}$ at three different buffer concentrations (0.047 M, 0.1123 M, 0.223 M) as the linearity should be independent of base concentration. The average of both plots' y-axis values was generated (Fig. S19), and the maximum y-axis value of this plot was found at a potential of 1.52 V. Therefore, $i_{cat}$ for **1b** will be taken at the potential of 1.52 V vs Ag/AgCl.

Other workers, such as the Llobet group, for foot-of-the-wave analysis (FOWA),[9] determine the instead of limiting current methods to determine catalytic rate constants. However, there are inherent issues as well as a few assumptions that are made when using foot-of-the-wave as described by Llobets group in 2016. One of said assumptions is the determination of the formal potential of catalysis $E^0_{cat}$ as because water is both the substrate and solvent one cannot remove or decrease the concentration of substrate to observe the true formal potential.

Previous work has used differential pulse voltammetry (DPV) or square wave voltammetry (SWV) to determine this formal potential, however because catalysis is a kinetic process the peak response obtained from DPV or SWV will shift depending on the scan rate (Fig. S14).

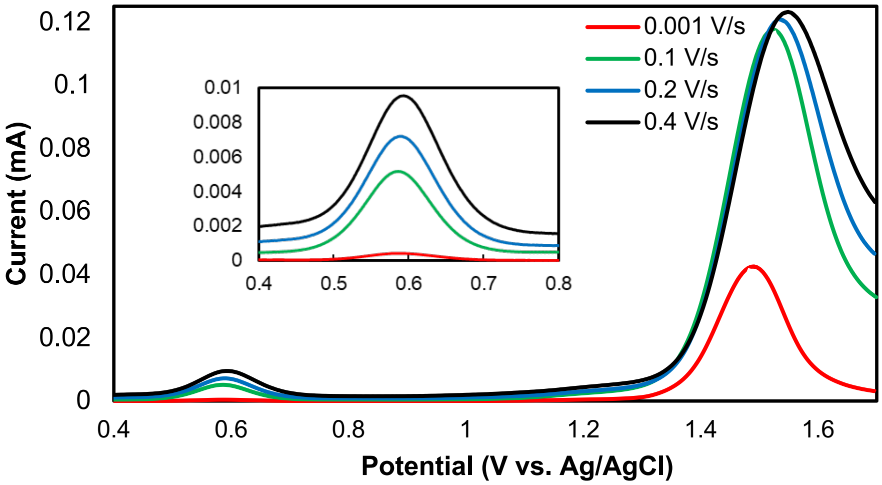


**Figure S17**: SWV of **1b** at 50uM concentration in pH = 7 and I =0.5 phosphate buffer solution.

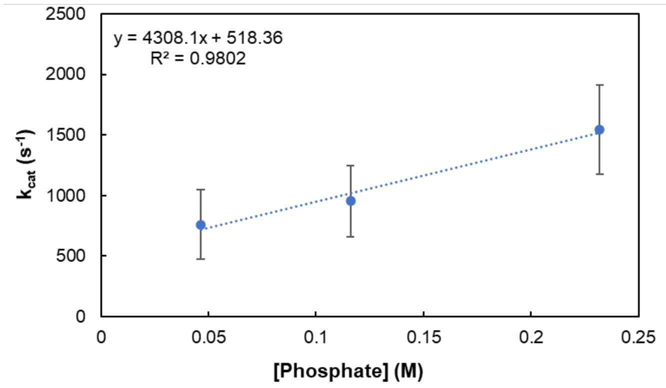


**Figure S18**: Scan rate independent plot of rate vs. buffer concentration of **1b** at $[cat]_0$ = 50 μM at 1.52V in pH = 7 phosphate buffer. The ionic strength of the solution normalized to I = 0.5 using $KNO_3$. BDD working electrode (S = 0.0707 $cm^2$), Ag/AgCl reference electrode, Pt wire counter electrode. The slope of the line represents $K_B$, the ratio of $K_{cat}$ to buffer ion concentration, while the intercept represents the values of $k_{H_2O}$ (see eq. 1).

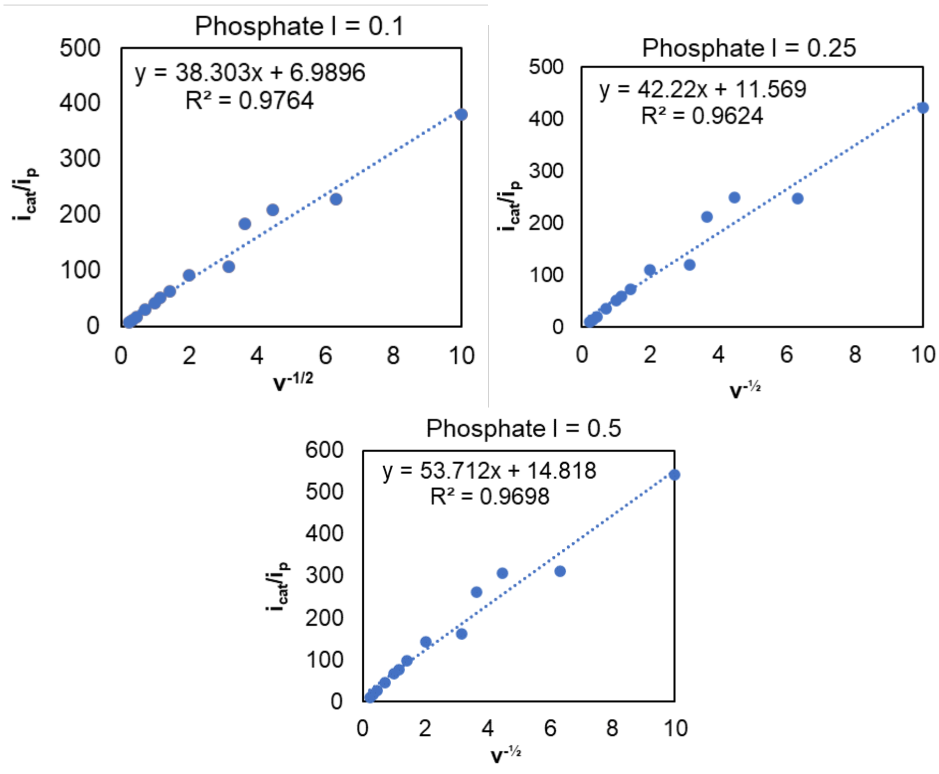


**Figure S19:** The plot of $i_{cat}/i_p$ vs. v $^{-½}$ of a 50 μM solution of **1b** in pH 7 phosphate buffer at multiple ionic strengths**,** v = 0.025 V/s, V = 1.46 V. BDD working electrode (S = 0.0707 $cm^2$), Ag/AgCl reference electrode, Pt wire counter electrode.

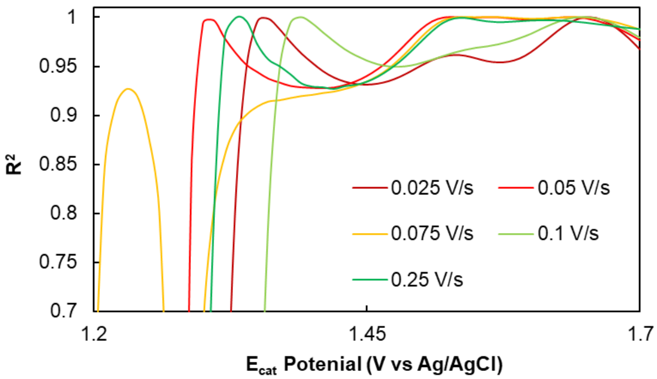


**Figure S20**: The plot for the fitted $R^2$ value of the $(i_{cat}/i_p)^2$ vs [B] with respect to various possible $E_{cat}$ potentials at multiple scan rates.

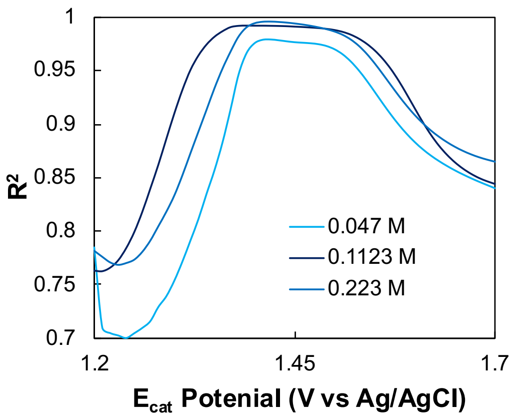


**Figure S21**: The plot for the fitted R2 value of the icat/ip vs. v $^{-½}$ with respect to various possible Ecat potentials at multiple pH 7 phosphate buffer concentrations.

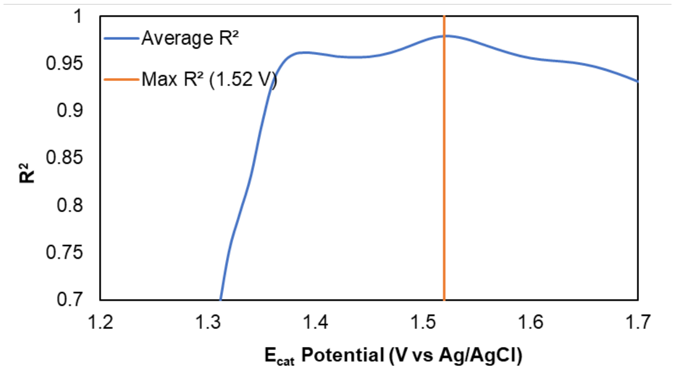


**Figure S22**: For **1b**, the average plot for the fitted $R^2$ values from the Figures S17 and S18 with respect to various possible $E_{cat}$ potentials. The maximum y value of this plot appears at 1.52 V, which is the potential at which we get the highest combined linear fit for both graphs.

## VI. Spectral Data for Starting Materials and Catalysts

*a.* $^{1}$HNMR experimental data

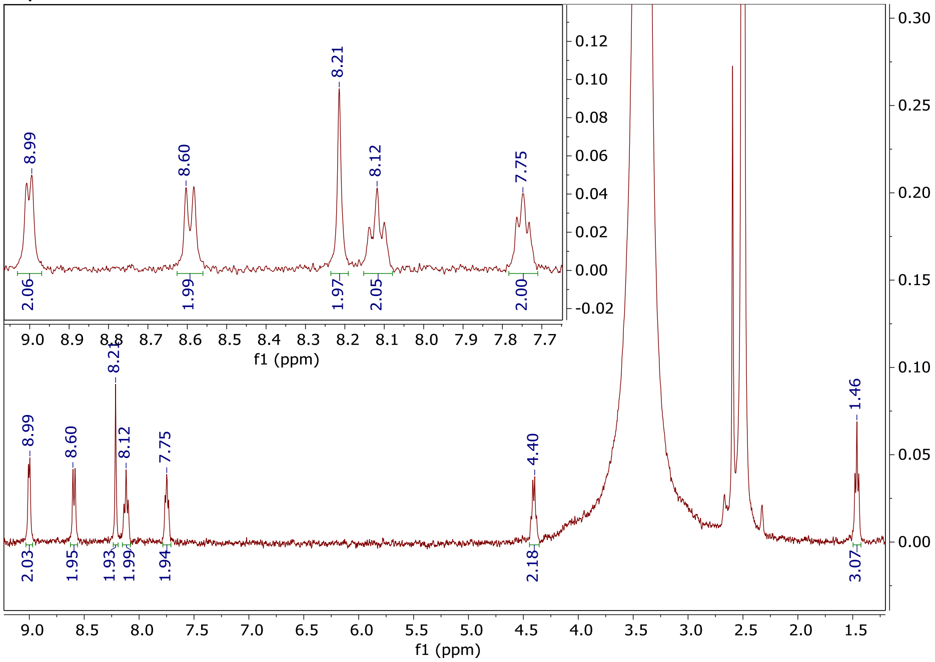

**Figure S23**: **C2,** Ru(EtO-terpy)$Cl_2$(DMSO): *$^{1}$H NMR (400 MHz, d-DMSO) δ 8.99 (d, J = 4.9 Hz, 2H), 8.60 (d, J = 7.2 Hz, 2H), 8.21 (s, 2H), 8.12 (s, 0H), 7.75 (t, J = 6.2, 6.1 Hz, 2H), 4.41 (q, J = 6.8, 6.6, 6.5, 6.5, 5.7 Hz, 3H), 1.46 (t, J = 6.9, 6.9, 6.9, 6.9 Hz, 2H).*

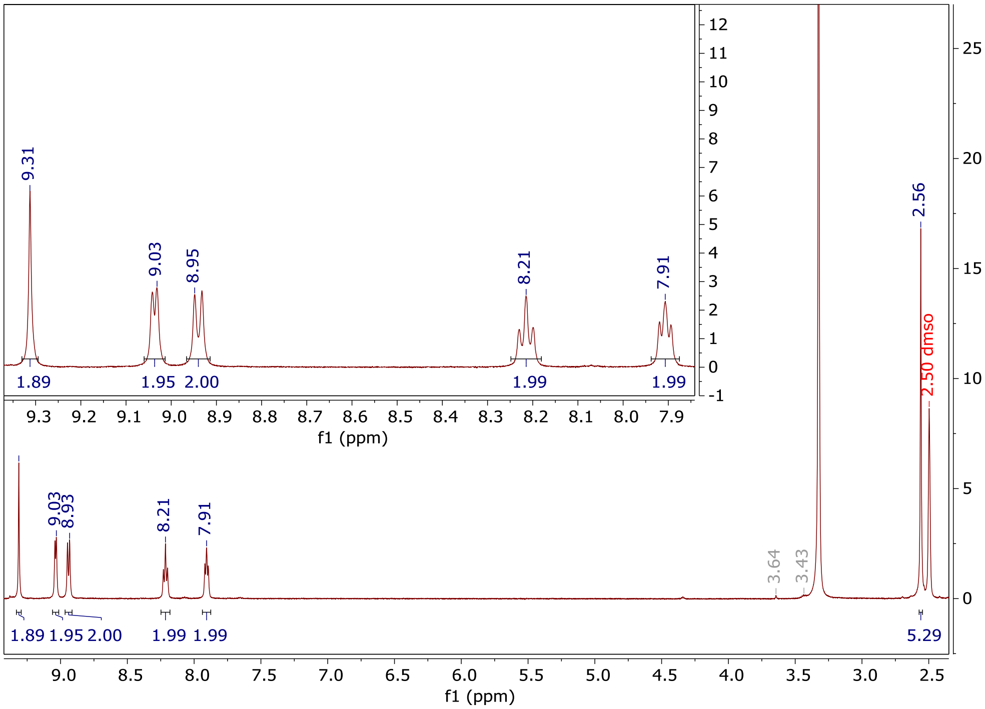

**Figure S24**: **C3,** Ru($NO_2$-terpy)$Cl_2$(DMSO): *$^{1}$H NMR (500 MHz, DMSO) δ 9.31 (s, 2H), 9.03 (s, 2H), 8.93 (s, 2H), 8.21 (s, 2H), 7.91 (s, 2H).*

**Elem. Anal. Calculated** for $C_{17}H_{16}Cl_2N_4O_3RuS$ (528.37): C, 38.64; H, 3.05; N, 10.60; O, 9.08; S, 6.07. Found: C, 38.26; H, 3.06; N, 10.53; S, 6.18.

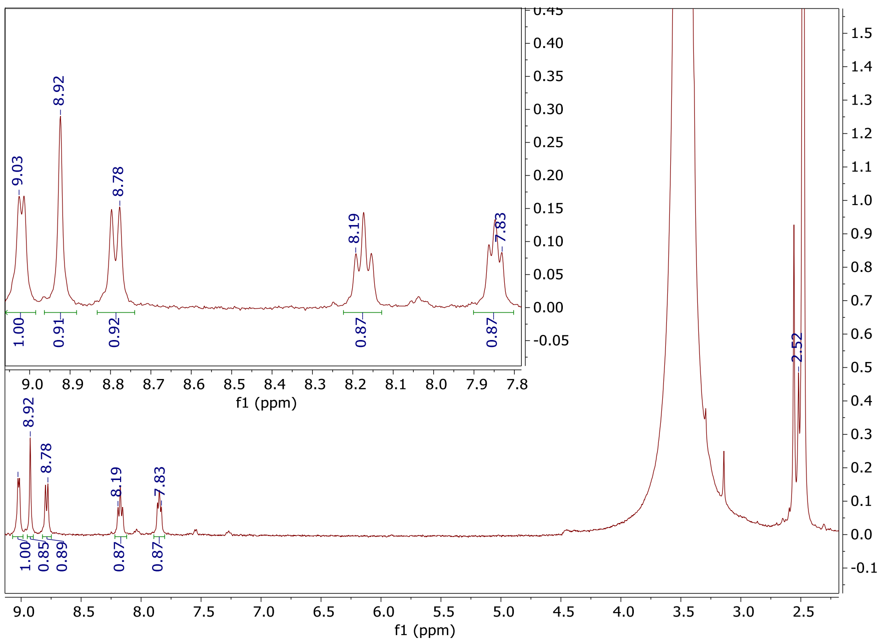


**Figure S25**: **C4,** Ru($CF_3$-terpy)$Cl_2$(DMSO): *$^1$H NMR (400 MHz, DMSO) δ 9.03 (s, 2H), 8.92 (s, 2H), 8.78 (s, 2H), 8.19 (s, 2H), 7.85 (s, 2H), 2.56 (s, 6H), 1.01 (s, 7H).*

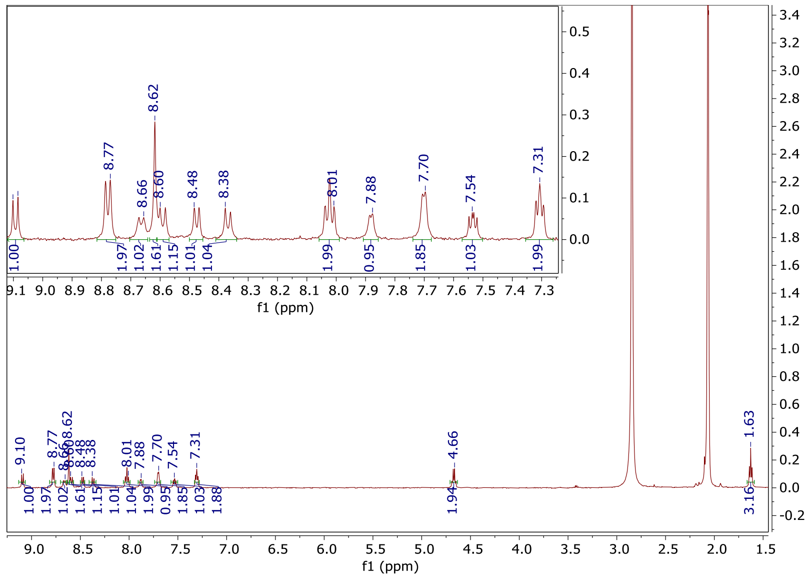


**Figure S26**: $^1$HNMR [Ru(EtO-tpy)(phen-$SO_3$)OTf] **1b**

$^1$H NMR (500 MHz, acetone) δ 9.10 (d, *J* = 8.5 Hz, 1H), 8.77 (d, *J* = 8.1 Hz, 2H), 8.66 (d, *J* = 8.2 Hz, 1H), 8.62 (s, 2H), 8.60 (d, *J* = 8.9 Hz, 1H), 8.48 (d, *J* = 8.1 Hz, 1H), 8.38 (d, *J* = 8.7 Hz, 1H), 8.01 (t, *J* = 7.6, 7.2 Hz, 2H), 7.88 (d, *J* = 4.5 Hz, 1H), 7.70 (d, *J* = 4.8 Hz, 2H), 7.54 (td, *J* = 5.4, 5.4, 2.6 Hz, 1H), 7.31 (t, *J* = 6.1, 6.1 Hz, 2H), 4.66 (q, *J* = 7.1, 7.0, 7.0 Hz, 1H), 1.63 (t, *J* = 7.2, 7.0 Hz, 2H).

**Elem. Anal. Calculated** for $C_{30}H_{22}F_3N_5O_7RuS_2$ (786.72): C, 45.80; H, 2.82; N, 8.90; S, 8.15. Found: C, 45.00; H, 2.91; N, 8.87; S, 7.94. Anal. Calculated for $C_{30}H_{22}F_3N_5O_7RuS_2$ + $H_2O$ (804.73): C, 44.78; H, 3.01; N, 8.70; S, 7.97.

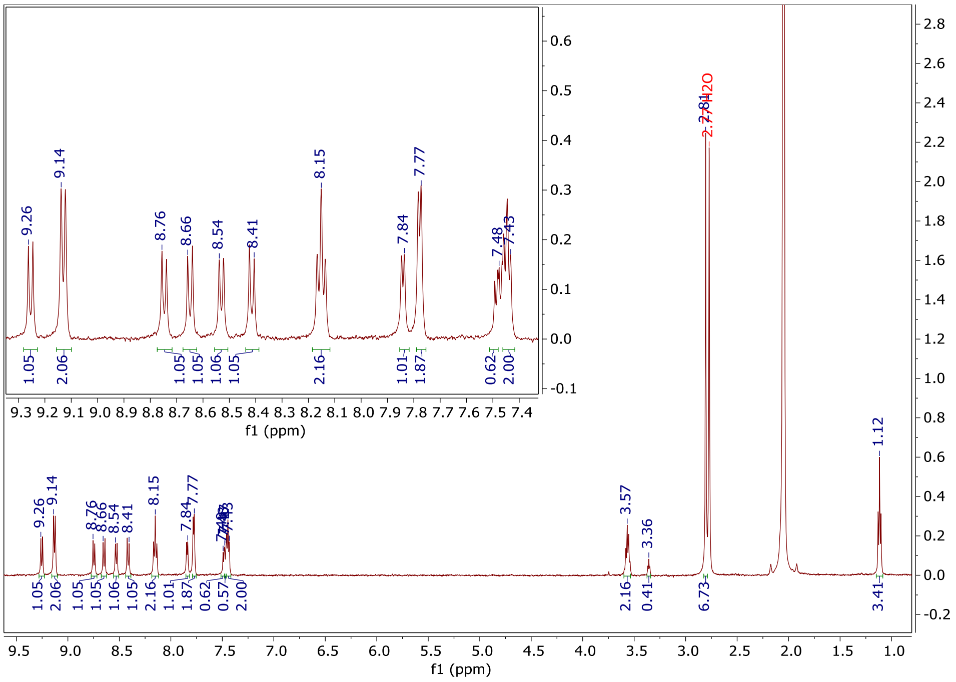

**Figure S27**: [Ru($NO_2$-tpy)(phen-$SO_3$)OTf] **1c**

$^{1}$H NMR (500 MHz, acetone) δ 9.26 (t, *J* = 8.6, 8.3 Hz, 1H), 9.14 (d, *J* = 8.0 Hz, 2H), 8.76 (d, *J* = 8.4 Hz, 1H), 8.66 (t, *J* = 9.3, 8.1 Hz, 1H), 8.54 (d, *J* = 8.3 Hz, 1H), 8.41 (d, *J* = 8.7 Hz, 1H), 8.15 (t, *J* = 8.0, 7.7 Hz, 2H), 7.84 (d, *J* = 5.9 Hz, 1H), 7.77 (d, *J* = 5.9 Hz, 2H), 7.49 (d, *J* = 4.8 Hz, 0H), 7.47 (d, *J* = 5.7, 5.5 Hz, 0H), 7.43 (t, *J* = 6.6, 6.3 Hz, 1H).

**Elem. Anal. Calculated** for $C_{28}H_{17}F_3N_6O_8RuS_2$ (787.66): C, 42.70; H, 2.18; N, 10.67; S, 8.14. Found: C, 43.12; H, 2.57; N, 10.16; S, 7.98. Anal. Calculated for $C_{28}H_{17}F_3N_6O_8RuS_2$ + $CH_3CH_2OH$ (833.73): C, 43.22; H, 2.78; N, 10.08; S, 7.69.

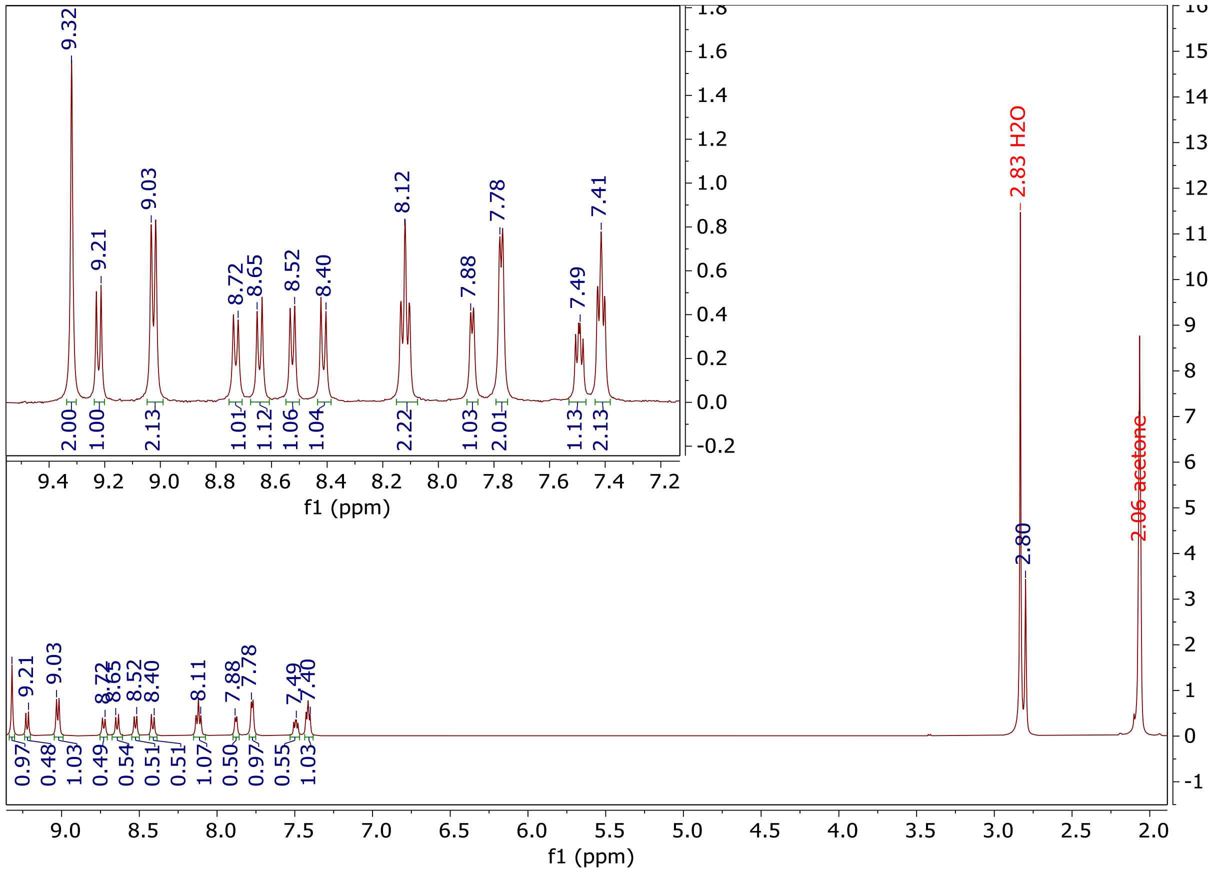

**Figure S28**: [Ru($CF_3$-tpy)(phen-$SO_3$)OTf] **1d**

$^{1}$H NMR (500 MHz, acetone) δ 9.32 (s, 1H), 9.21 (d, *J* = 9.0 Hz, 1H), 9.03 (d, *J* = 7.5 Hz, 1H), 8.72 (d, *J* = 9.0 Hz, 0H), 8.65 (d, *J* = 9.7 Hz, 0H), 8.52 (d, *J* = 10.1 Hz, 0H), 8.40 (d, *J* = 8.2 Hz, 0H), 8.11 (dd, *J* = 8.2, 6.3 Hz, 0H), 7.88 (d, *J* = 5.6 Hz, 0H), 7.78 (d, *J* = 6.3 Hz, 1H), 7.49 (ddd, *J* = 9.3, 5.6, 0.7 Hz, 0H), 7.40 (t, *J* = 6.3, 5.6 Hz, 0H). Elem. Anal. Calculated for C29H17F6N5O6RuS2 (810.66): C, 42.97; H, 2.11; N, 8.64; S, 7.91. Found: C, 43.48; H, 2.41; N, 8.19; S, 7.68. Anal. Calculated for C29H17F6N5O6RuS2 + CH3CH2OH (856.73): C, 43.46; H, 2.71; N, 8.17; S, 7.48.

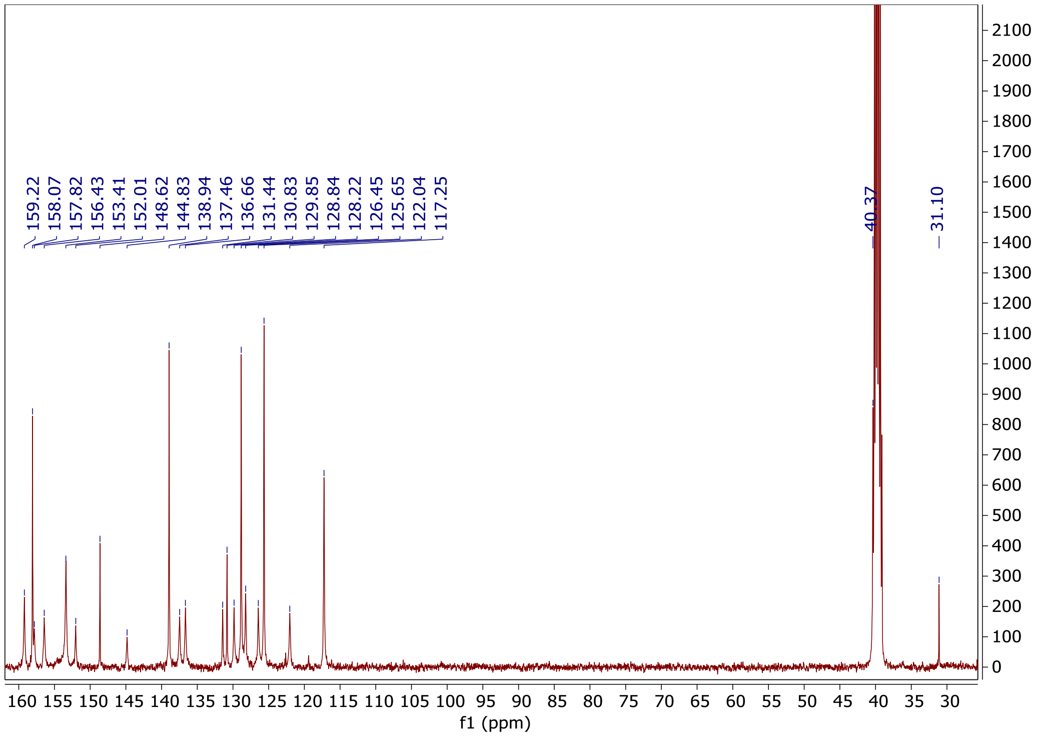


**Figure S29**: [Ru($NO_2$-tpy)(phen-$SO_3$)OTf] **1c** $^{13}$CNMR

## VII. Crystal data and structure refinement for 1b and 1c.

**Table S5. Crystal data and structure refinement for 1b and 1c.**

| Compound | **1b** | **1c** |
|---|---|---|
| **Empirical formula** | $C_{32}H_{29}F3N_5O_8RuS_2$ | $C_{33}$ $H_{30}$ $F_3$ $N_6$ $O_9$ Ru $S_2$ |
| **Formula weight** | 843.50 | 876.82 |
| **Wavelength (Å)** | 0.71073 | 0.71073 |
| **Crystal system** | Monoclinic | Triclinic |
| **Space group** | P 1 21/c 1 | P-1 |
| **a (Å)** | 16.3100 | 8.4705(16) |
| **b (Å)** | 13.1444 | 13.862(3) |
| **c (Å)** | 16.9082 | 14.962(3) |
| **α (°)** | 90 | 82.992(5) |
| **β (°)** | 109.386 | 80.324(5) |
| **γ (°)** | 90 | 81.531(6) |
| **Volume ($Å^3$)** | 3419.3 | 1704.4(5) |
| **Z** | 4 | 2 |
| **Density (calculated) ($Mg/m^3$)** | 1.639 | 1.709 |
| **Absorption coefficient (0.665 $mm^{-1}$)** | 0.658 | 0.665 |
| **F(000)** | | 890 |
| **Crystal size ($mm^3$)** | 0.14 x 0.1 x 0.02 | 0.2 x 0.2 x 0.03 |
| **Crystal color, habit** | | red plate |
| **Theta range for data collection** | 2.008 to 25.681° | 1.388 to 26.044° |
| **Index ranges** | -14<=h19, -16<=k, -20<=1<20 | -10<=h<=10, -17<=k<=17, -18<=l<=18 |
| **Reflections collected** | 31407 | 41757 |
| **Independent reflections** | 6478 [R(int) = 0.0451] | 6743 [R(int) = 0.0522] |
| **Completeness to theta = 25.242°** | 99.8 % | 100.0 % |
| **Absorption correction** | Semi-empirical from equivalents | Semi-empirical from equivalents |
| **Max. and min. transmission** | 0.4906 and 0.4481 | 0.5624 and 0.5191 |
| **Refinement method** | Full-matrix least squares on $F^2$ | Full-matrix least-squares on $F^2$ |
| **Data / restraints / parameters** | 6478 / 125 / 564 | 6743 / 139 / 506 |
| **Goodness-of-fit on $F^2$** | 1.139 | 1.038 |
| **Final R indices [I>2σ(I)]** | R1 = 0.0544, wR2 = 0.1250 | R1 = 0.0388, wR2 = 0.0906 |
| **R indices (all data)** | R1 = 0.1680, wR2 = 0.1319 | R1 = 0.0504, wR2 = 0.0951 |
| **Largest diff. peak and hole** | 1.860 and -1.135 e.Å-3 | 0.636 and -0.783 e.$Å^{-3}$ |

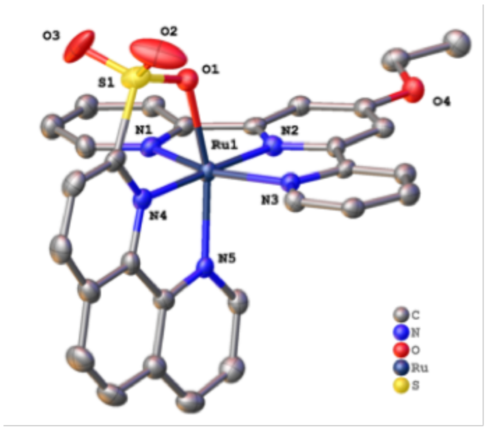


**1b**

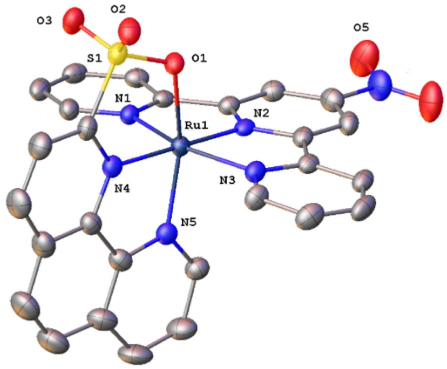


**1c**

**Figure S30:** Crystal structure of the **1b** and **1c** catalysts.

**Table S6**: Selected bond lengths for **1a-1c**

| Complex | Ru1-O1 | Ru1-N1 | Ru1-N2 | Ru1-N3 | Ru1-N4 | Ru1-N5 | S1-O1 | S1-O2 | S1-O3 |
|---|---|---|---|---|---|---|---|---|---|
| **1a**[21] | 2.180 | 2.065 | 1.969 | 2.069 | 2.003 | 2.040 | 1.468 | 1.453 | 1.446 |
| **1b** | 2.177 | 2.067 | 1.981 | 2.066 | 2.000 | 2.027 | 1.477 | 1.416 | 1.439 |
| **1c** | 2.174 | 2.067 | 1.958 | 2.067 | 2.009 | 2.034 | 1.494 | 1.436 | 1.441 |

**Table S7**: Selected bond angles for **1a-1c**

| Complex | O1-Ru1-N4 | O1-Ru1-N2 | S1-O1-Ru1 | N2-Ru1-N5 | S1-C44-N4 | C44-N4-Ru1 | C44-S1-O1 |
|---|---|---|---|---|---|---|---|
| **1a**[21] | 79.076 | 102.056 | 116.354 | 98.307 | 111.550 | 124.860 | 103.1935 |
| **1b** | 79.504 | 101.605 | 118.407 | 98.372 | 112.721 | 124.457 | 104.1843 |
| **1c** | 79.109 | 102.363 | 116.925 | 98.172 | 111.962 | 124.744 | 103.3755 |

## VIII. Computational Data

Geometry optimizations and frequency calculations for all reported structures were performed with the Gaussian 16 suite of programs[1] at the B3LYP-D3BJ/[6-31G(d,p) + Lanl2dz (Ru)] level of theory with the corresponding Hay-Wadt effective core potential for Ru, and Grimme's empirical dispersion-correction (D3) with Becke-Johnson (BJ) damping for B3LYP.[2-5] Frequency analysis was used to characterize each minimum with zero imaginary frequencies. Bulk solvent effects are incorporated for all calculations using the self-consistent reaction field polarizable continuum model (IEF-PCM).[6] As a solvent we chose water. The Gibbs free energies are calculated at 298.15 K temperature and 1 atm. pressure.

The redox process is modeled as a series of discrete one-electron or one-proton losses. The solid-state structure of **1a**, and **1b** were used to generate an energy minimized structure. A water molecule was then introduced near the metal center of the of **1a** and **1b** catalysts and the geometry optimized. Several bonding motifs were isolated, and the lowest energy structures of [**1b-OH$_2$**] and [**1d-OH$_2$**] were found to exhibit a water ligated to the metal center, with each OH unit donating a hydrogen bond to one oxygen of the sulfonate (see Figures S26 and S27). Similar geometry motive was previously reported for the [**1a-OH$_2$**] structure.[7] These structures were used as the basis for subsequent calculations.

One electron oxidation of [**1b-OH$_2$**] and [**1d-OH$_2$**] (S = 0, singlet) is predicted to afford the [**1b-OH$_2$**]$^+$ and [**1d-OH$_2$**]$^+$ cations which are a Ru(III) (doublets with ρ(Ru) = 0.92 and 0.92 |e|, respectively) complexes. Next, the [**1b-OH$_2$**]$^+$ and [**1d-OH$_2$**]$^+$ cations are predicted to undergo deprotonation by bulk water to yield other doublet

species [**1b-OH**] and [**1d-OH**], respectively, now exhibiting radical character on both the Ru (ρ(Ru) = 0.66 and 0.65 |e|, respectively) and the newly formed hydroxo ligand [ρ($O^w$) = 0.32 and 0.33 |e|, respectively).

Consistent with our previous finding for the **1a** catalyst,[7] the presented calculations suggest that 1$e^-$ oxidation of [**1b-OH**] and [**1d-OH**] species results in diradical species of [**1b-OH**]$^+$ and [**1d-OH**]$^+$ (triplets with: ρ(Ru) = 1.09 and ρ($O^w$) = 0.85 |e| for [**1b-OH**]$^+$, and ρ(Ru) = 1.06 and ρ($O^w$) = 0.89 |e| for [**1d-OH**]$^+$) and a proton transfer event wherein the proton *is transferring to the sulfonate.* That is to say, the weakly basic sulfonate acts a pendant base, deprotonating the oxidized hydroxo ligand, as was previously reported for the **1a** catalyst.[7] The sulfonate is then predicted to transfer the proton to bulk water affording [**1b-O**] and [**1d-O**] species, a Ru(III)-oxyl complex (triplets ρ(Ru) = 0.90 and ρ($O^w$) = 1.05 |e| for [**1b-O**], and ρ(Ru) = 0.88 and ρ($O^w$) = 1.08 |e| for [**1d-O**])). At this point the Ru(II)-$OH_2$ starting material has lost 2$e^-$ and 2$H^+$.

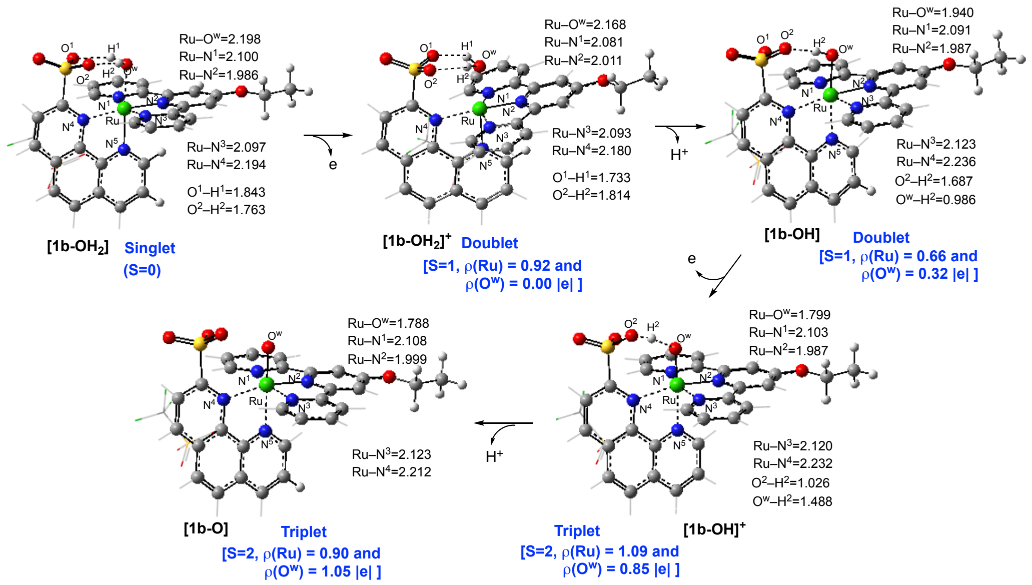


**Figure S31:** The calculated intermediates of the initial ETPTs by the **1b** catalyst. The presented distances are in Å, and spin densities are in |e|.

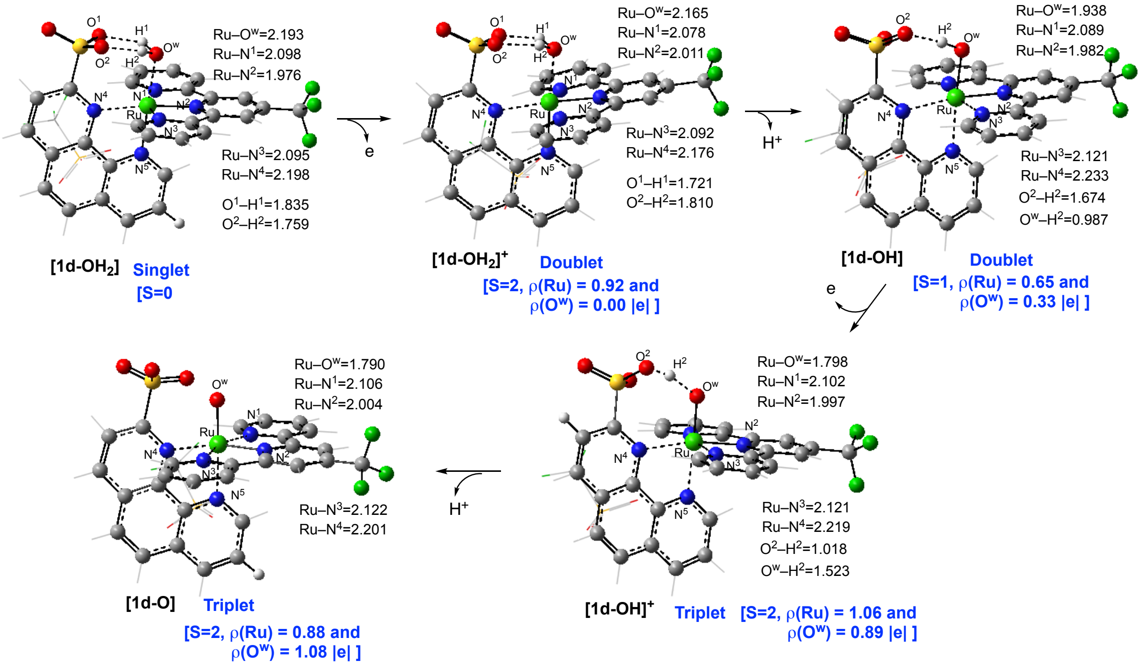


**Figure S32:** The calculated intermediates of the initial ETPTs by the **1d** catalyst. The presented distances are in Å, and spin densities are in |e|.

**REFERENCES (Computational):**


1. Gaussian 16, Revision A.03, M. J. Frisch, G. W. Trucks, H. B. Schlegel, G. E. Scuseria, M. A. Robb, J. R. Cheeseman, G. Scalmani, V. Barone, G. A. Petersson, H. Nakatsuji, X. Li, M. Caricato, A. Marenich, J. Bloino, B. G. Janesko, R. Gomperts, B. Mennucci, H. P. Hratchian, J. V. Ortiz, A. F. Izmaylov, J. L. Sonnenberg, D. Williams-Young, F. Ding, F. Lipparini, F. Egidi, J. Goings, B. Peng, A. Petrone, T. Henderson, D. Ranasinghe, V. G. Zakrzewski, J. Gao, N. Rega, G. Zheng, W. Liang, M. Hada, M. Ehara, K. Toyota, R. Fukuda, J. Hasegawa, M. Ishida, T. Nakajima, Y. Honda, O. Kitao, H. Nakai, T. Vreven, K. Throssell, J. A. Montgomery, Jr., J. E. Peralta, F. Ogliaro, M. Bearpark, J. J. Heyd, E. Brothers, K. N. Kudin, V. N. Staroverov, T. Keith, R. Kobayashi, J. Normand, K. Raghavachari, A. Rendell, J. C. Burant, S. S. Iyengar, J. Tomasi, M. Cossi, J. M. Millam, M. Klene, C. Adamo, R. Cammi, J. W. Ochterski, R. L. Martin, K. Morokuma, O. Farkas, J. B. Foresman, and D. J. Fox, *Gaussian, Inc., Wallingford CT*, **2016**.
2. (a) Hay, P. J.; Wadt, W. R. Ab Initio Effective Core Potentials for Molecular Calculations. Potentials for the Transition Metal Atoms Sc to Hg. *J. Chem. Phys.* **1985**, *82*, 270-283. (b) Hay, P. J.; Wadt, W. R. Ab Initio Effective Core Potentials for Molecular Calculations. Potentials for K to Au Including the Outermost Core Orbitals. *J. Chem. Phys.* **1985**, *82*, 299-310. (c) Wadt, W. R.; Hay, P. J. Ab Initio Effective Core Potentials for Molecular Calculations. Potentials for Main Group Elements Na to Bi. *J. Chem. Phys*. **1985**, *82*, 284-298.
3. (a) Becke, A. D. Density-Functional Exchange-Energy Approximation with Correct Asymptotic Behavior. *Phys. Rev. A* **1988,** *38*, 3098-3100. (b) Lee, C.; Yang, W.; Parr, R. G. Development of The Colle-Salvetti Correlation-Energy Formula into a Functional of the Electron Density. *Phys. Rev. B* **1988,** *37*, 785-789. (c) Becke, A. D. A New Mixing of Hartree–Fock and Local Density-Functional Theories. *J. Chem. Phys.* **1993,** *98*, 1372-1377.
4. Grimme, S., Hansen, A., Brandenburg, J. G. & Bannwarth, C. Dispersion-Corrected Mean-Field Electronic Structure Methods. *Chem. Rev.* **116**, 5105-5154 (2016).
5. (a) Grimme, S.; Antony, J.; Ehrlich, S.; Krieg, H. A Consistent and Accurate Ab Initio Parametrization of Density Functional Dispersion Correction (DFT-D) for the 94 Elements H-Pu. *J. Chem. Phys.* **2010,** *132*, 154104-154122. (b) Becke, A. D.; Johnson, E. R. A Density-Functional Model of the Dispersion Interaction. *J. Chem. Phys.* **2005,** *123*, 154101-154106. (c) Becke, A. D.; Johnson, E. R. Exchange-Hole Dipole Moment and the Dispersion Interaction. *J. Chem. Phys.* **2005,** *122*, 154104-154109. (d) Johnson, E. R.; Becke, A. D. A Post-Hartree-Fock Model of Intermolecular Interactions: Inclusion of Higher-Order Corrections. *J. Chem. Phys.* **2006,** *124*, 174104-174112.
6. (a) Barone, V.; Cossi, M. Quantum Calculation of Molecular Energies and Energy Gradients in Solution by a Conductor Solvent Model. *J. Phys. Chem. A* **1998,** *102*, 1995-2001. (b) Cossi, M.; Rega, N.; Scalmani, G.; Barone, V. Energies, structures, and electronic properties of molecules in solution with the C-PCM solvation model. *J. Comput. Chem.* **2003,** *24*, 669-681.
7. Nash, A. G.; Breyer, C. J.; Vincenzini, B. D.; Elliott, G. I.; Niklas, J.; Poluektov, O. G.; Rheingold, A. L.; Smith, D. K.; Musaev, D. G.; Grotjahn, D. B. *Angew. Chem. Int. Ed.* **2021**, *60*, 1540–1545.

## IX. Cartesian Coordinates of the Calculated Structures

**1a, X = H**
Ru 2.62754800 7.82675300 4.19086100
S 3.47351900 6.56817200 6.96942900
O 3.55258100 7.89503500 6.20913500
O 4.70170200 6.30847400 7.74259100
O 2.19649300 6.41329300 7.69670900
N 4.48858300 8.31931500 3.34718800
N 2.36529700 9.77050700 3.80118400
N 2.00612400 7.05436800 2.36449300
N 2.94603600 5.83952200 4.51185300
N 0.68064600 8.12081500 4.91499100
C 5.53539300 7.50377000 3.15258900
H 5.40931400 6.47967600 3.48273900
C 6.71807800 7.93711400 2.56112200
H 7.53660800 7.24072800 2.42424100
C 6.81697400 9.26663600 2.15680600
H 7.72295600 9.63840900 1.69164800
C 5.73359100 10.11936200 2.35736100
H 5.79108700 11.15623800 2.05101900
C 4.57380100 9.62881100 2.95551300
C 3.37574200 10.45345600 3.21545000
C 3.21136400 11.80762400 2.92259500
H 4.00391000 12.37556500 2.45247200
C 2.00122300 12.42286900 3.24755500
H 1.85643500 13.47401100 3.02665300
C 0.97776100 11.69596100 3.85638600
H 0.04114900 12.17650200 4.10865100
C 1.18527400 10.34304400 4.13067500
C 0.23423900 9.40815300 4.76356600
C -1.03829100 9.77856500 5.19214900
H -1.37866200 10.79896900 5.06811700
C -1.86675400 8.82539700 5.78247700
H -2.85958300 9.10181200 6.11952400
C -1.40321800 7.52140300 5.92589600
H -2.01734600 6.74537300 6.36385500
C -0.12299200 7.20858800 5.47733800
H 0.27994500 6.21086300 5.56198000
C 1.48099300 7.67131000 1.30725000
H 1.31319300 8.73740800 1.40130200
C 1.16302900 6.98438000 0.12273100
H 0.73793200 7.54017000 -0.70459400
C 1.39732000 5.62563900 0.02328800
H 1.16037000 5.08415700 -0.88633700
C 1.94757600 4.94145500 1.12772400
C 2.23512500 3.53227400 1.15596500
H 2.01842700 2.95255200 0.26518800
C 2.75768600 2.92023900 2.26073900
H 2.96384900 1.85533700 2.25341400
C 3.02587600 3.66836400 3.45587900
C 3.53836000 3.14414000 4.66759800
H 3.77726200 2.08820000 4.73564600
C 3.72916700 3.97289600 5.76539300
H 4.12101600 3.59089900 6.70010600
C 3.41033300 5.33328800 5.64469400
C 2.74608200 5.04832300 3.43140300
C 2.22392400 5.70138700 2.27938700
S -1.47973600 4.54184400 2.81026200
F -1.39227200 2.49896800 4.50066000
F -1.66706700 4.43022200 5.46007700
F 0.28555200 3.87125200 4.68717100
O -0.83027400 5.87211300 2.86064300
O -0.89430600 3.58906500 1.84065700
O -2.96062600 4.56712500 2.84849300
C -1.04115200 3.79242300 4.45120100

**1b, X = OEt**
Ru 2.63038600 7.77110800 4.24040000
S 3.38022400 6.33803700 6.96434700
O 3.52188400 7.70023300 6.28131400
O 4.58231300 5.98922900 7.74421700
O 2.08470400 6.19213200 7.66102800
N 4.52673100 8.22235100 3.44916800
N 2.46013300 9.74242800 3.91285700
N 2.01520200 7.11958000 2.36752400
N 2.86788400 5.76119100 4.46415800
N 0.68889900 8.13966900 4.95113600
C 5.54699700 7.37409000 3.25344100
H 5.37516900 6.34625700 3.54944500
C 6.75927200 7.77888300 2.70305200
H 7.55407300 7.05586800 2.56420000
C 6.91788700 9.11484300 2.34192900
H 7.84805500 9.46564800 1.90941400
C 5.86256500 10.00188400 2.54419900
H 5.96751700 11.04447100 2.27175700
C 4.67123500 9.53938700 3.10039000
C 3.49785100 10.40240300 3.36021600
C 3.40254600 11.76531200 3.08480400
H 4.23253800 12.29353600 2.63994800
C 2.20410900 12.42771500 3.39714700
O 1.98064400 13.73337200 3.18167100
C 1.13856300 11.71246300 3.97530400
H 0.22304800 12.24016700 4.20845300
C 1.29489400 10.35772700 4.22504800
C 0.29467000 9.44619600 4.81984600
C -0.96892300 9.85691600 5.23632400
H -1.26793900 10.89181800 5.12584400
C -1.84205800 8.92567500 5.79723200
H -2.82941900 9.23355500 6.12301500
C -1.42786300 7.60403400 5.92970700
H -2.07449100 6.84471900 6.35019200
C -0.15352600 7.25090700 5.49446400
H 0.21648400 6.24146800 5.57689000
C 1.53258900 7.81038800 1.33479600
H 1.38655700 8.87288800 1.48813500

C 1.23598900 7.20236200 0.10277500
H 0.84500500 7.81469500 -0.70112800
C 1.45576800 5.84813700 -0.07474300
H 1.24407200 5.36969200 -1.02513400
C 1.96009700 5.08706800 1.00145900
C 2.23561600 3.67629100 0.94807000
H 2.04995200 3.15745500 0.01354000
C 2.70856100 2.98909900 2.03034000
H 2.90631800 1.92475500 1.96171300
C 2.93851500 3.65539000 3.28051700
C 3.41351800 3.05034400 4.46995200
H 3.63609300 1.98867500 4.47898900
C 3.59367400 3.80892400 5.61892500
H 3.96053900 3.36589900 6.53669200
C 3.30136800 5.17990600 5.57291700
C 2.67753200 5.03802100 3.33456200
C 2.20613800 5.76929200 2.20837800
S -1.36959500 4.36832000 3.00877000
F -1.29707200 2.46591300 4.85565000
F -1.64894100 4.45788700 5.65174500
F 0.34223000 3.88942700 4.99099300
O -0.77313600 5.72438900 2.98223100
O -0.70672400 3.37028700 2.14084800
O -2.85081400 4.33796400 2.98683300
C -0.97346700 3.75982500 4.71704400
C 3.02371900 14.53494300 2.58693200
H 3.91293300 14.49692200 3.22641700
H 3.27945700 14.11869600 1.60586100
C 2.49053200 15.94730300 2.46563300
H 3.25460400 16.58835500 2.01770700
H 1.60090200 15.97183700 1.83072000
H 2.23275900 16.34941700 3.44899700

**1d, X = $CF_3$**
Ru 2.62131500 7.77542300 4.23740900
S 3.36962700 6.35965000 6.96525400
O 3.50673900 7.71615000 6.26538800
O 4.57216500 6.02817100 7.75001700
O 2.07273600 6.21561700 7.65797400
N 4.51747400 8.22109900 3.44763300
N 2.44186700 9.73438000 3.91396400
N 2.01082300 7.10691400 2.36401100
N 2.86659700 5.76273300 4.46940600
N 0.67792300 8.12120100 4.94537900
C 5.53891800 7.37491100 3.25174000
H 5.36961400 6.34630200 3.54591700
C 6.75049700 7.78425100 2.70255200
H 7.54702000 7.06334400 2.56308000
C 6.90692100 9.12101400 2.34343000
H 7.83670600 9.47405000 1.91214500
C 5.85007100 10.00605500 2.54619400
H 5.95246200 11.04950700 2.27608800
C 4.66028900 9.53793100 3.10092900
C 3.48719300 10.39516800 3.36279200
C 3.37611900 11.75817200 3.09635600
H 4.18983900 12.31054300 2.64636500
C 2.17934300 12.40257500 3.41262800
C 1.11577900 11.70227800 3.98286100
H 0.19011100 12.21088700 4.21653500
C 1.27298300 10.33959100 4.22991300
C 0.27562700 9.42514400 4.81625300
C -0.99227600 9.82847000 5.22719300
H -1.29860300 10.86132500 5.11821700
C -1.86150500 8.88958900 5.78135600
H -2.85237600 9.18986100 6.10315100
C -1.43908400 7.57051400 5.91207600
H -2.08294000 6.80605100 6.32720900
C -0.16036600 7.22547600 5.48200200
H 0.21478200 6.21794600 5.56325100
C 1.52512300 7.78970400 1.32790900
H 1.37250500 8.85221100 1.47386700
C 1.23188600 7.17327400 0.09919200
H 0.83809500 7.77934100 -0.70796700
C 1.45830900 5.81941600 -0.07035700
H 1.24902700 5.33456500 -1.01798100
C 1.96558600 5.06674100 1.01038400
C 2.24658000 3.65676400 0.96526700
H 2.06317200 3.13195700 0.03372500
C 2.72165600 2.97752800 2.05159000
H 2.92359100 1.91365300 1.98936800
C 2.94756500 3.65208500 3.29789900
C 3.42249800 3.05608600 4.49187800
H 3.64925000 1.99540600 4.50738300
C 3.59696400 3.82110500 5.63733400
H 3.96313800 3.38453300 6.55839100
C 3.29965000 5.19063500 5.58240100
C 2.68098600 5.03400200 3.34374200
C 2.20803000 5.75716300 2.21310400
S -1.36540000 4.37394100 2.98755200
F -1.30196600 2.44756200 4.81013300
F -1.65040500 4.43005700 5.63105100
F 0.34063400 3.86530700 4.96728900
O -0.75997100 5.72649600 2.97941000
O -0.70703300 3.38313400 2.10811200
O -2.84651800 4.35338900 2.96301500
C -0.97505200 3.74204700 4.68884200
C 2.05197100 13.88755600 3.18645700
F 0.78099100 14.23891200 2.90556200
F 2.42418000 14.57891200 4.28576000
F 2.83132300 14.30432500 2.16859900

**[1a–$OH_2$]**

Ru 2.61602000 7.90729600 4.23469400
S 3.71237200 5.92995500 7.18086300
O 4.85326100 6.85247300 6.86340600
O 4.05669800 4.87033200 8.14955000
O 2.44650800 6.66203600 7.50229100
N 4.49549300 8.36984700 3.43105900
N 2.31764200 9.78994300 3.69305200
N 2.02559100 7.12189800 2.43037600
N 2.96277500 5.76391500 4.56573900
N 0.64458900 8.19183800 4.89490200
C 5.57276300 7.57475500 3.35050500
H 5.45772700 6.57975700 3.76191200
C 6.76894900 7.99941400 2.77923800
H 7.61315800 7.32184600 2.73540200
C 6.84894300 9.29552200 2.27516600
H 7.76469800 9.65820400 1.82216400
C 5.73447200 10.12816500 2.36140300
H 5.77799100 11.14027300 1.97931900
C 4.56400100 9.64822900 2.94548700
C 3.33490600 10.45449900 3.09917200
C 3.14919700 11.77974800 2.70414600
H 3.94485400 12.33268700 2.22177300
C 1.91453300 12.38517800 2.94270100
H 1.75272300 13.41305700 2.63953200
C 0.88887900 11.67987900 3.57315100
H -0.06544900 12.15425300 3.76259000
C 1.11775400 10.35637600 3.95277500
C 0.17003800 9.44968800 4.63254500
C -1.11865600 9.82066300 5.00664300
H -1.48309200 10.81809700 4.79498600
C -1.93216700 8.89601600 5.66174500
H -2.93831400 9.17174600 5.95738700
C -1.43603000 7.62381000 5.92672800
H -2.03614400 6.87212600 6.42277300
C -0.13954900 7.30936400 5.52574700
H 0.29744600 6.33941800 5.70678600
C 1.49577100 7.81001400 1.41676000
H 1.32238200 8.86492700 1.58098100
C 1.18344100 7.21321200 0.18770800
H 0.75525300 7.82243800 -0.59927500
C 1.43942500 5.86927400 -0.00093800
H 1.22197000 5.38530600 -0.94717100
C 1.98564200 5.11653600 1.05833200
C 2.27577300 3.71734500 0.95597000
H 2.08249700 3.21863900 0.01224300
C 2.76549800 3.02748600 2.02137900
H 2.97493200 1.96567700 1.94788500
C 2.99809400 3.67730900 3.27746500
C 3.46200300 2.99029000 4.41473400
H 3.66153200 1.92531700 4.35961400
C 3.64812700 3.68627000 5.58966700
H 3.99163100 3.20519200 6.49493800
C 3.39100300 5.06601700 5.62083800
C 2.75125500 5.06673600 3.40338200
C 2.24789400 5.78652800 2.27520300
S -1.30129300 4.35001900 3.15123800
F -1.14187900 2.48811500 5.03254500
F -1.54996100 4.48222800 5.79567800
F 0.45039300 3.96731700 5.12000100
O -0.75616400 5.72634900 3.09028300
O -0.61268700 3.36006500 2.29426800
O -2.78066500 4.26449300 3.14908700
C -0.86292000 3.78942900 4.86552800
O 3.24666600 8.80984300 6.13395000
H 2.66829200 8.33632300 6.77230900
H 4.09847300 8.35307100 6.33465800

**[1b–OH$_2$]**
Ru 2.67261000 7.86503000 4.28256600
S 3.69905800 5.80100100 7.19170700
O 4.86455900 6.70090400 6.89981500
O 4.01205100 4.71257600 8.13931200
O 2.45061900 6.55761700 7.52416600
N 4.56733800 8.30837900 3.50036100
N 2.42594100 9.76988500 3.77736900
N 2.07592200 7.13588000 2.45887000
N 2.96061800 5.70930400 4.57018700
N 0.70295100 8.19337500 4.93413500
C 5.62682100 7.49082800 3.40807200
H 5.48551800 6.48948400 3.79528100
C 6.83685200 7.90009100 2.85498700
H 7.66514600 7.20381800 2.80092400
C 6.95098400 9.20525100 2.38220000
H 7.87813500 9.55703100 1.94404200
C 5.85530200 10.06131500 2.48057400
H 5.92619700 11.08055400 2.12230700
C 4.66954000 9.59625900 3.04481200
C 3.45615400 10.42778500 3.20919100
C 3.32044400 11.76473200 2.84056500
H 4.14319800 12.29151500 2.38094700
C 2.09386400 12.40426900 3.08475300
O 1.83044300 13.68233400 2.77635400
C 1.04319800 11.69448900 3.69690500
H 0.10873000 12.20662200 3.88491100
C 1.23911100 10.36618400 4.04051800
C 0.26192500 9.46791600 4.69358600
C -1.02038100 9.86326500 5.06325400
H -1.35755400 10.87344000 4.86704400
C -1.86305500 8.94702000 5.69271500
H -2.86481600 9.24210100 5.98461500
C -1.40102800 7.65794500 5.93713800
H -2.02402900 6.91203400 6.41328400

C -0.10941900 7.31907900 5.54097500
H 0.30180400 6.33521400 5.70687500
C 1.57414400 7.86011100 1.45589400
H 1.43150900 8.91607100 1.64166900
C 1.25401900 7.29819500 0.21250300
H 0.84976000 7.93580700 -0.56464900
C 1.47196100 5.95134300 -0.00255800
H 1.24757100 5.49360500 -0.96019100
C 1.98832900 5.16126900 1.04443700
C 2.23883200 3.75664500 0.91523100
H 2.03795300 3.28301400 -0.03976200
C 2.70132900 3.03137700 1.96928700
H 2.88050100 1.96563900 1.87508300
C 2.94463500 3.64838700 3.23986700
C 3.38275500 2.92567300 4.36522400
H 3.55262600 1.85679900 4.28967500
C 3.58220700 3.59260600 5.55480400
H 3.90750200 3.08406700 6.45177300
C 3.36351600 4.97811400 5.61277100
C 2.73639700 5.04138400 3.39278900
C 2.26098400 5.79806800 2.27676600
S -1.32724200 4.42112100 3.11354200
F -1.22807800 2.53054500 4.97008100
F -1.59434700 4.52313900 5.75777900
F 0.39846600 3.96959400 5.09052500
O -0.74608400 5.78336400 3.07428200
O -0.65847900 3.42425800 2.24884900
O -2.80840700 4.37460500 3.09965300
C -0.91648800 3.82697500 4.82338600
O 3.31152000 8.71651800 6.20551800
H 2.71707800 8.24353800 6.82891300
H 4.14951300 8.23307500 6.40054700
C 2.85811000 14.48067800 2.14987400
H 3.73266400 14.51823700 2.80906300
H 3.15065200 14.00895900 1.20501500
C 2.27758200 15.86080400 1.92250000
H 3.02837800 16.49829600 1.44789900
H 1.40244100 15.80975000 1.26932100
H 1.98332700 16.31794200 2.87093500

**[1d–OH$_2$]**

Ru 2.67047100 7.86097600 4.31236800
S 3.71082800 5.75625500 7.18868900
O 4.87622400 6.65746800 6.90055400
O 4.02488000 4.65651400 8.12170200
O 2.46462000 6.51170500 7.53251700
N 4.56120000 8.30252000 3.52435400
N 2.42228700 9.76524400 3.84622700
N 2.06067800 7.15227500 2.48106500
N 2.95895900 5.69759700 4.56955900
N 0.70829700 8.16814800 4.98698300
C 5.61525900 7.48185600 3.40737600
H 5.47316300 6.47348000 3.77525400
C 6.82231700 7.89783500 2.85201600
H 7.64698900 7.19930700 2.77708200
C 6.93870900 9.21134600 2.40370300
H 7.86367800 9.56710600 1.96439600
C 5.84817700 10.07068300 2.52781700
H 5.91989500 11.09629500 2.18840500
C 4.66635800 9.59778400 3.09367500
C 3.46019900 10.42823000 3.28569400
C 3.31649700 11.77523900 2.95892200
H 4.12691900 12.33321200 2.50998700
C 2.10045400 12.40082800 3.23553800
C 1.05291900 11.70193800 3.83601600
H 0.12171900 12.20309200 4.06321000
C 1.24092500 10.35503700 4.14122500
C 0.26667300 9.44781100 4.77871100
C -1.01336300 9.83692500 5.16239500
H -1.35298300 10.85036200 4.98904600
C -1.85256800 8.90660700 5.77602700
H -2.85272000 9.19503600 6.07938700
C -1.38941300 7.61270700 5.98931000
H -2.01020100 6.85687600 6.45231600
C -0.10002500 7.28075500 5.57828300
H 0.31073800 6.29304700 5.71961200
C 1.55202200 7.88867300 1.49113100
H 1.40856900 8.94219800 1.68921800
C 1.22411900 7.34250000 0.24277700
H 0.81399800 7.98965600 -0.52320200
C 1.44250100 5.99899800 0.00963100
H 1.21250900 5.55310600 -0.95221900
C 1.96650800 5.19619700 1.04305000
C 2.21740800 3.79375800 0.89368100
H 2.01133800 3.33288100 -0.06632800
C 2.68617800 3.05494700 1.93539300
H 2.86548900 1.99072200 1.82631100
C 2.93573000 3.65551200 3.21247400
C 3.37985700 2.91844800 4.32610700
H 3.54962200 1.85073800 4.23568900
C 3.58517700 3.56928900 5.52347400
H 3.91482600 3.04889500 6.41200500
C 3.36698700 4.95380100 5.60085000
C 2.72791600 5.04628100 3.38489600
C 2.24591000 5.81734500 2.28169900
S -1.32516400 4.43994000 3.11593700
F -1.23365800 2.50911600 4.93121200
F -1.58537600 4.48652600 5.76262300
F 0.40222100 3.93527600 5.07831000
O -0.73236200 5.79798200 3.10473400
O -0.66628900 3.45673000 2.22854800
O -2.80648300 4.40641400 3.10469600

C -0.91452300 3.80609900 4.81160700
O 3.32386900 8.67798800 6.23927500
H 2.73380900 8.19912000 6.86342300
H 4.16436500 8.19397800 6.42500500
C 1.89636100 13.83981100 2.83278400
F 1.38648300 13.92298300 1.58485800
F 1.03728500 14.46884500 3.65911600
F 3.05574600 14.52688600 2.83881200

**[1a–OH2]+**
Ru 2.72315600 7.90269200 4.29373500
S 3.87035600 6.19332600 7.33642700
O 4.97922900 7.11886700 6.94250600
O 4.22134000 5.22099600 8.38294400
O 2.57554000 6.91796400 7.57006800
N 4.47424100 8.39755500 3.26055100
N 2.34371600 9.80194900 3.75036700
N 2.12492700 7.00841700 2.54410400
N 3.09842300 5.80487000 4.74593200
N 0.73192700 8.08985900 4.85428000
C 5.49911900 7.56620100 3.01556900
H 5.38646900 6.54490800 3.35501300
C 6.64960800 8.00308300 2.36878400
H 7.46073000 7.30768800 2.19332500
C 6.72648800 9.33267800 1.95975100
H 7.60858300 9.70108700 1.44893700
C 5.65581600 10.19235900 2.20527000
H 5.69744500 11.22591000 1.88687000
C 4.53032300 9.70518100 2.85986500
C 3.32746300 10.50417500 3.15379900
C 3.12452300 11.85477200 2.87167900
H 3.89338900 12.44303700 2.38844300
C 1.90602600 12.43482100 3.22663300
H 1.73120700 13.48341800 3.01724500
C 0.90924300 11.68003500 3.84539200
H -0.03590600 12.13313400 4.11405200
C 1.15663100 10.33219100 4.10624300
C 0.24054300 9.36471600 4.73475900
C -1.04076700 9.66628900 5.17377100
H -1.42840200 10.67226400 5.07954400
C -1.82156500 8.65511600 5.73934700
H -2.82226900 8.87958600 6.09017800
C -1.31326400 7.36499900 5.83442400
H -1.89697000 6.55214200 6.24369000
C -0.02775200 7.11167800 5.36493400
H 0.41186000 6.12601100 5.40014500
C 1.61190400 7.64813800 1.49328000
H 1.48100500 8.71810900 1.58024500
C 1.25974000 6.96871100 0.32068000
H 0.84665800 7.53165400 -0.50690300
C 1.43823100 5.60184900 0.24460400
H 1.16524600 5.05388300 -0.64983500
C 1.97676900 4.90710600 1.34691700
C 2.19081900 3.49066100 1.36311700
H 1.92426400 2.92087700 0.48058500
C 2.70804900 2.87434200 2.46196700
H 2.86715600 1.80191400 2.47369800
C 3.03870800 3.62126600 3.64003600
C 3.53848600 3.03088600 4.81543100
H 3.71315100 1.96095400 4.85017700
C 3.78611700 3.82154100 5.92026800
H 4.15291700 3.40878800 6.85001900
C 3.55128500 5.20122300 5.84936600
C 2.83258500 5.02162900 3.65303300
C 2.30457800 5.65911500 2.49316700
S -1.51950800 4.82930300 2.56839200
F -1.54950800 2.56835500 3.95992700
F -1.74723000 4.36127600 5.17392600
F 0.18605200 3.82584400 4.34016500
O -0.77938800 6.09222800 2.81057900
O -0.98425200 3.98695400 1.47527800
O -2.99343300 4.95395300 2.60537100
C -1.14148000 3.83675200 4.09065700
O 3.28210600 8.94779800 6.10330100
H 2.76994700 8.45063600 6.79679200
H 4.18456000 8.61435200 6.33415200

**[1b–OH2]+**
Ru 2.78558000 7.85457600 4.36827900
S 3.84636300 6.00703500 7.35861200
O 4.98846100 6.91088800 7.01423500
O 4.15240900 4.98787700 8.37477900
O 2.57081300 6.76201400 7.60121700
N 4.55437100 8.33633200 3.35838400
N 2.46448200 9.77564900 3.86884300
N 2.17411900 7.03674300 2.58422400
N 3.09512900 5.73017000 4.74687100
N 0.79327600 8.09439900 4.91810700
C 5.55631200 7.48212300 3.09729600
H 5.41429600 6.45743400 3.41506300
C 6.71968500 7.89971100 2.46102400
H 7.51125800 7.18558700 2.27198500
C 6.83386700 9.23455500 2.07914300
H 7.72636300 9.58875700 1.57639300
C 5.78655500 10.11787700 2.34073900
H 5.85759500 11.15565100 2.04194000
C 4.64686000 9.64931400 2.98385100
C 3.46046800 10.47158100 3.28941500
C 3.30573700 11.82593900 3.02053800
H 4.10052300 12.38314400 2.54821400
C 2.09187200 12.44412700 3.37619000
O 1.81409700 13.72907500 3.17376200

C 1.07121200 11.68431700 3.98572300
H 0.14136000 12.17234400 4.24549200
C 1.28785600 10.33970300 4.22435700
C 0.33948100 9.38475800 4.82706500
C -0.93486900 9.71255800 5.26703000
H -1.29223100 10.73149600 5.19366700
C -1.74748900 8.71180800 5.80491200
H -2.74339800 8.95682000 6.15580000
C -1.27655700 7.40567500 5.87257900
H -1.88516400 6.60020900 6.25954600
C 0.00328700 7.12676200 5.40305900
H 0.41477700 6.12855400 5.41747900
C 1.68407900 7.72668900 1.55456100
H 1.58267600 8.79610600 1.67982900
C 1.31921800 7.09844400 0.35721500
H 0.92520100 7.70106400 -0.45155700
C 1.46220700 5.73089300 0.23286800
H 1.17998400 5.22263900 -0.68198700
C 1.97577600 4.98341900 1.31224300
C 2.15159400 3.56175900 1.28014500
H 1.87595600 3.03051400 0.37659300
C 2.64352800 2.89327500 2.35993400
H 2.77295400 1.81703900 2.33566800
C 2.98486900 3.58918300 3.56579800
C 3.45724000 2.94481500 4.72398900
H 3.60197700 1.86986000 4.72254200
C 3.71545000 3.68930800 5.85822400
H 4.06078500 3.23434200 6.77640100
C 3.51919700 5.07652500 5.83338100
C 2.81801200 4.99344300 3.62573600
C 2.31665900 5.68590300 2.48588600
S -1.51922500 4.92770000 2.55286600
F -1.61147600 2.64797200 3.91076500
F -1.78213200 4.42659100 5.14940100
F 0.14722200 3.86121200 4.32602900
O -0.75229000 6.16975600 2.81764900
O -0.99530500 4.08809000 1.45227800
O -2.99028300 5.08566700 2.58050100
C -1.17727800 3.90523100 4.06409000
O 3.35617300 8.82851300 6.21941100
H 2.82218300 8.32173400 6.88788900
H 4.24654800 8.46658100 6.44993700
C 2.80069000 14.58395100 2.54096100
H 3.03203500 14.17855500 1.55035600
H 3.71054000 14.58013700 3.15003900
C 2.19651500 15.96852100 2.44976900
H 2.91307100 16.64444600 1.97562200
H 1.28319900 15.95383500 1.84954200
H 1.96073500 16.35452000 3.44478100

**[1d–OH$_2$]$^+$**

Ru 2.77796600 7.84895600 4.35884900
S 3.85555200 6.01243200 7.34798900
O 4.99564400 6.91775600 7.00144600
O 4.16185900 4.99544400 8.36540100
O 2.57836400 6.76669100 7.58790500
N 4.54686100 8.32691700 3.34851300
N 2.45336700 9.77373200 3.87423500
N 2.15821800 7.02983200 2.57970400
N 3.09725800 5.73034500 4.73788900
N 0.79202400 8.07472100 4.92580000
C 5.54925700 7.47667100 3.07952900
H 5.40960400 6.44911400 3.38857600
C 6.71178400 7.90196200 2.44506200
H 7.50374100 7.19018600 2.24906300
C 6.82499400 9.23963800 2.07447900
H 7.71660300 9.59886900 1.57391800
C 5.77703400 10.12047700 2.34518100
H 5.84622400 11.16119100 2.05620500
C 4.63977400 9.64342500 2.98508100
C 3.45686300 10.46368300 3.29848200
C 3.29252700 11.82541800 3.05160600
H 4.07683800 12.41106500 2.59212000
C 2.08796200 12.42205600 3.42232700
C 1.06847600 11.68337300 4.02206200
H 0.14184700 12.16083300 4.31024100
C 1.27989500 10.32365900 4.24322400
C 0.33549200 9.36562300 4.84225300
C -0.93759900 9.69188900 5.28501100
H -1.29827900 10.70984500 5.21541500
C -1.74794500 8.68746600 5.82185400
H -2.74321100 8.93007900 6.17582600
C -1.27481100 7.38270700 5.88405200
H -1.88133400 6.57571800 6.27113500
C 0.00487100 7.10543800 5.40948100
H 0.41684400 6.10748400 5.42046800
C 1.65780000 7.71653300 1.55246400
H 1.55165100 8.78566700 1.67540400
C 1.28754400 7.08544200 0.35876100
H 0.88532500 7.68543900 -0.44783700
C 1.43546600 5.71813200 0.23530900
H 1.14872500 5.20773400 -0.67687300
C 1.96004700 4.97394200 1.31171700
C 2.14097600 3.55297100 1.28039400
H 1.86138800 3.01939800 0.37947400
C 2.64239400 2.88798300 2.35805300
H 2.77558000 1.81222700 2.33462800
C 2.98863200 3.58689300 3.56070400
C 3.46953200 2.94628400 4.71749400
H 3.61845800 1.87194500 4.71725500
C 3.72971500 3.69367200 5.84928500
H 4.08022400 3.24140000 6.76685000

C 3.52815100 5.08015600 5.82387400
C 2.81685500 4.99051500 3.61928400
C 2.30611900 5.67902900 2.48203000
S -1.52352600 4.92310100 2.57319700
F -1.61084600 2.63299800 3.91418700
F -1.76806100 4.40292400 5.16726100
F 0.15360000 3.83985000 4.32479700
O -0.75275000 6.16175600 2.84537200
O -1.00626300 4.09317900 1.46230900
O -2.99382600 5.08333200 2.61084500
C -1.17298200 3.88780000 4.07350700
O 3.35754000 8.82308300 6.20383800
H 2.82919500 8.31440500 6.87759500
H 4.25129100 8.46587500 6.43129300
C 1.85824500 13.88769000 3.12255800
F 1.25526400 14.03417500 1.92693600
F 1.06796800 14.45572000 4.05113000
F 3.01771200 14.56778500 3.08352500

**[1a–OH]**
Ru 2.63006800 7.97515000 4.31268900
S 3.30158800 5.82751400 7.36269700
O 4.42641100 6.81912800 7.31402800
O 3.56621100 4.70302600 8.28810400
O 1.96137700 6.44559000 7.50188800
N 4.44817200 8.34072300 3.28355400
N 2.34811900 9.85362800 3.73497800
N 1.96614600 7.13532300 2.48154900
N 2.93812200 5.78648200 4.64450800
N 0.65786600 8.27118100 4.93405600
C 5.45855700 7.48778400 3.07491100
H 5.32881100 6.48681500 3.46675700
C 6.61434800 7.86551600 2.39727900
H 7.41023900 7.14605100 2.24936800
C 6.71480600 9.17188600 1.92598900
H 7.60038700 9.50026900 1.39386400
C 5.66267800 10.06097800 2.14225500
H 5.72502800 11.07877700 1.77934100
C 4.53058800 9.62560300 2.82655600
C 3.35331000 10.48115300 3.08953400
C 3.19359300 11.81565800 2.71601500
H 3.98081400 12.34650100 2.19682200
C 1.99332300 12.45905300 3.02285000
H 1.85391300 13.49617700 2.74117100
C 0.96962900 11.77842200 3.68137600
H 0.03689000 12.27773000 3.90925300
C 1.17291500 10.44366900 4.03701600
C 0.20968000 9.54381800 4.70076500
C -1.08158500 9.91608100 5.05825500
H -1.43258000 10.92321500 4.87273500
C -1.92056400 8.97298500 5.65478600
H -2.93101300 9.24924600 5.93470900
C -1.44995000 7.68412400 5.87891300
H -2.07266600 6.92096100 6.32677700
C -0.14543600 7.36782400 5.50505900
H 0.28543800 6.39502800 5.67594900
C 1.39695300 7.80380300 1.48073900
H 1.14481000 8.84038400 1.66561600
C 1.14532600 7.21124100 0.23617000
H 0.67778200 7.79576600 -0.54674100
C 1.51932900 5.89768700 0.03039100
H 1.35811200 5.41685500 -0.92882600
C 2.10972300 5.16548500 1.08143600
C 2.51033200 3.79776600 0.94886800
H 2.38600400 3.31242600 -0.01309800
C 3.01197600 3.11929800 2.01526600
H 3.30076400 2.07749900 1.92686900
C 3.14568600 3.74983400 3.29517900
C 3.58904100 3.05197600 4.43458800
H 3.86182400 2.00496900 4.35393400
C 3.63639900 3.70543600 5.64472100
H 3.92359400 3.20513400 6.55893300
C 3.29587800 5.06748200 5.71037900
C 2.81443800 5.11885300 3.45001400
C 2.29452700 5.82789500 2.31718100
S -1.13426800 4.31223200 3.16509400
F -1.07825700 2.50590000 5.10496500
F -1.55272200 4.51703100 5.78221300
F 0.49107700 4.00529100 5.24586200
O -0.67907000 5.72217100 3.12715600
O -0.30833400 3.36288300 2.38741100
O -2.59861200 4.12659300 3.03978800
C -0.80276000 3.80462100 4.91763200
O 3.37078200 8.80995100 5.89894200
H 3.80616400 8.12775000 6.46260900

**[1b–OH]**
Ru 2.68706700 7.92735200 4.37727400
S 3.28318200 5.68166800 7.37277500
O 4.43362200 6.64434700 7.35659800
O 3.51300900 4.52585800 8.26885200
O 1.95872100 6.33036500 7.52310100
N 4.52064300 8.27682100 3.36536200
N 2.46151300 9.82737600 3.84022400
N 2.01593300 7.15444700 2.51972200
N 2.93455800 5.72261100 4.65290900
N 0.71721600 8.27027500 4.98865100
C 5.50873300 7.40270900 3.13881400
H 5.35036600 6.39640000 3.50584500
C 6.67667800 7.76499300 2.47363000
H 7.45339900 7.02801500 2.31060700
C 6.81344300 9.07892200 2.03399800

H 7.70902900 9.39631000 1.51204000
C 5.78427700 9.99017100 2.26860800
H 5.87551400 11.01386400 1.92920200
C 4.63867500 9.56979700 2.93899200
C 3.48024700 10.44950600 3.21849000
C 3.37300500 11.79340400 2.87214100
H 4.18625600 12.29619600 2.37092100
C 2.18332200 12.47333300 3.18867900
O 1.95387800 13.76175100 2.91482000
C 1.13331400 11.78750900 3.82954200
H 0.22105400 12.32367900 4.05519200
C 1.30085300 10.44947600 4.14743000
C 0.30554000 9.55945300 4.78075800
C -0.97908900 9.95742700 5.13328500
H -1.30110300 10.97729000 4.96519100
C -1.84912200 9.02450400 5.70035300
H -2.85492300 9.32142400 5.97595200
C -1.41506500 7.71892800 5.90080200
H -2.06221700 6.96297500 6.32568700
C -0.11596400 7.37653700 5.53216600
H 0.28767700 6.38900500 5.68475000
C 1.47563000 7.86537400 1.53229300
H 1.25144300 8.90325000 1.74378400
C 1.22027500 7.31413200 0.26959500
H 0.77735300 7.93257600 -0.50153100
C 1.55951400 5.99650400 0.03166300
H 1.39507100 5.54638300 -0.94179200
C 2.11870800 5.21993100 1.06781300
C 2.48252400 3.84559400 0.90160500
H 2.35340800 3.38991100 -0.07417000
C 2.95573600 3.12521100 1.95340400
H 3.21653700 2.07853100 1.83936800
C 3.09589800 3.71733800 3.25085400
C 3.51077700 2.97705000 4.37406100
H 3.75426000 1.92512600 4.26723500
C 3.56832000 3.59684200 5.60134000
H 3.83541300 3.06474300 6.50359100
C 3.26574000 4.96581800 5.70102400
C 2.80120900 5.09042000 3.44019900
C 2.30996900 5.84343900 2.32279800
S -1.16496500 4.39063800 3.11671000
F -1.17387000 2.54916800 5.02402200
F -1.60611700 4.55887800 5.73283200
F 0.43015200 4.00658200 5.20775100
O -0.67774700 5.79025200 3.10945200
O -0.35214700 3.43665500 2.33081100
O -2.63167700 4.24061900 2.97189600
C -0.86464100 3.84397000 4.86304700
O 3.43316100 8.70460600 5.99076200
H 3.84900400 7.99758000 6.53708000
C 2.97770000 14.53721600 2.24926000
H 3.20191800 14.07319900 1.28250900
H 3.88451200 14.52967100 2.86389600
C 2.43538400 15.94043300 2.07803400
H 3.18342200 16.56008900 1.57631600
H 1.52666500 15.93269500 1.47047500
H 2.20751800 16.38860400 3.04868400

**[1d–OH]**

Ru 2.68032400 7.92355700 4.37088300
S 3.28565800 5.68286900 7.36558500
O 4.43658300 6.64558500 7.34686200
O 3.51570900 4.52805600 8.26178200
O 1.96220500 6.33356100 7.51425800
N 4.51082900 8.27022500 3.35766400
N 2.45041400 9.82246100 3.85023800
N 2.00175800 7.14998800 2.51625200
N 2.93230000 5.72193900 4.64540000
N 0.71641400 8.25324800 5.00099900
C 5.49746900 7.39728800 3.12314900
H 5.34073000 6.38863500 3.48401700
C 6.66376700 7.76482600 2.45679400
H 7.43969700 7.02864800 2.28683200
C 6.80058000 9.08122400 2.02530300
H 7.69484800 9.40125900 1.50292000
C 5.77272000 9.99196800 2.26910000
H 5.86235200 11.01815700 1.93674600
C 4.63018600 9.56544400 2.94010800
C 3.47585500 10.44226700 3.22940400
C 3.35843600 11.79198000 2.90381100
H 4.16180400 12.32337000 2.41212500
C 2.17584800 12.45412700 3.23585100
C 1.13049900 11.78744500 3.87322400
H 0.22068600 12.31364300 4.12842900
C 1.29346000 10.43580900 4.17617700
C 0.30280000 9.54416500 4.80777900
C -0.97911600 9.94091800 5.17034900
H -1.30348700 10.96159100 5.01242500
C -1.84563900 9.00249500 5.73480500
H -2.84971000 9.29679500 6.01885900
C -1.40991400 7.69589000 5.92118400
H -2.05441600 6.93660000 6.34405600
C -0.11252800 7.35524000 5.54175800
H 0.29103500 6.36642400 5.68404500
C 1.45228400 7.85784500 1.53151200
H 1.22253900 8.89460900 1.74205400
C 1.19229900 7.30495300 0.27066300
H 0.74172800 7.92118400 -0.49771300
C 1.53695500 5.98910400 0.03101700
H 1.36940500 5.53811800 -0.94146000
C 2.10530300 5.21540200 1.06430200
C 2.47432400 3.84265800 0.89676200

H 2.34331300 3.38647300 -0.07846500
C 2.95402800 3.12412800 1.94689700
H 3.21839100 2.07847900 1.83208200
C 3.09587000 3.71684700 3.24382000
C 3.51589300 2.97861000 4.36648900
H 3.76259200 1.92746700 4.25973600
C 3.57394300 3.59901300 5.59343800
H 3.84407700 3.06787000 6.49535600
C 3.26768700 4.96700700 5.69369000
C 2.79734500 5.08888500 3.43335900
C 2.29984200 5.83988800 2.31806300
S -1.15708100 4.38657000 3.12866400
F -1.16573000 2.53305500 5.02467000
F -1.59898500 4.53840500 5.74544900
F 0.43764900 3.99025600 5.21725600
O -0.67222200 5.78731900 3.13256600
O -0.34071700 3.44030800 2.33737300
O -2.62309500 4.23515500 2.98061400
C -0.85735500 3.82865100 4.87152800
O 3.43756500 8.69205900 5.98093200
H 3.85457800 7.98435200 6.52862600
C 2.00116700 13.90146200 2.84089100
F 1.46221400 13.99731300 1.60765800
F 1.18268000 14.54958400 3.69127500
F 3.17983500 14.55246100 2.81840900

**[1a–OH]$^+$, $^3$A**

Ru 2.60719400 7.98178600 4.36508100
S 3.21882200 5.73389200 7.32653800
O 4.40868100 6.78915400 7.35779400
O 3.59623800 4.65536400 8.23188900
O 1.92709000 6.40272600 7.48037400
N 4.43809800 8.32766900 3.35619500
N 2.31640600 9.84773500 3.72219100
N 1.92778600 7.19610000 2.46310300
N 2.88677900 5.78904000 4.60447300
N 0.61726300 8.28693800 4.96926200
C 5.46241000 7.47924000 3.21230300
H 5.34572800 6.50005700 3.65814000
C 6.62155900 7.83856000 2.52937300
H 7.42934500 7.12388600 2.43333400
C 6.70935100 9.11685300 1.98687100
H 7.59700500 9.42829500 1.44856500
C 5.64226900 10.00250800 2.14058800
H 5.69664800 11.00000700 1.72458000
C 4.50866200 9.58864900 2.83267400
C 3.31994600 10.44171300 3.04012600
C 3.13922600 11.74794200 2.58917200
H 3.91736300 12.26038500 2.03965700
C 1.92607200 12.38609400 2.85515800
H 1.77155500 13.40164300 2.51011600
C 0.90531700 11.73259400 3.54681500
H -0.03723700 12.22994400 3.73268300
C 1.12425200 10.42648500 3.98339000
C 0.16731600 9.54958800 4.68615600
C -1.12216400 9.93355000 5.02952000
H -1.47366400 10.93321000 4.80917400
C -1.96095800 9.01110500 5.66102800
H -2.97056300 9.29781200 5.93231400
C -1.49135500 7.73184300 5.92767400
H -2.11293400 6.98324000 6.40077900
C -0.18578500 7.40208800 5.56488700
H 0.23823800 6.43242500 5.76338100
C 1.35597600 7.87745800 1.47320300
H 1.08546900 8.90607800 1.67217000
C 1.11749100 7.30658500 0.21562000
H 0.64362300 7.90113800 -0.55546500
C 1.51453000 6.00506300 -0.01721200
H 1.36837500 5.54357400 -0.98793500
C 2.11067700 5.25980900 1.02201100
C 2.54478200 3.90517100 0.85593200
H 2.44121500 3.44592200 -0.12093100
C 3.05685400 3.20790600 1.90440700
H 3.37569500 2.17824200 1.78841400
C 3.16220900 3.80487000 3.20201000
C 3.62539100 3.09091600 4.32027500
H 3.92806200 2.05518900 4.21287200
C 3.66157900 3.70928400 5.55282400
H 3.96898900 3.18539800 6.44692800
C 3.27918500 5.05244300 5.64723000
C 2.78975000 5.16154400 3.39028200
C 2.27192500 5.89620600 2.27303300
S -0.97880000 4.30758000 3.29400900
F -1.01659600 2.53820200 5.26956700
F -1.58222800 4.55010500 5.87161200
F 0.50537200 4.07848700 5.48233400
O -0.60099000 5.74185500 3.27626100
O -0.03194000 3.40468800 2.60398800
O -2.41242700 4.03638700 3.04715000
C -0.76234400 3.83793500 5.07461700
O 3.31779200 8.52123200 5.92587900
H 4.09015900 7.61788900 6.85319900

**[1a–OH]$^+$, $^1$A, open-shell**

Ru 2.61087400 7.98547800 4.36918100
S 3.21289600 5.73808100 7.33216300
O 4.39623800 6.79743900 7.36067600
O 3.59195600 4.66129800 8.23921600
O 1.91763000 6.40136300 7.48313200
N 4.43683100 8.32730500 3.35374100
N 2.31791100 9.84749700 3.72398700
N 1.92950700 7.19776300 2.46948800

N 2.88705000 5.79140600 4.60933700
N 0.62161400 8.28638300 4.97076400
C 5.45870200 7.47652400 3.20598500
H 5.34250400 6.49820700 3.65374500
C 6.61510200 7.83297900 2.51697100
H 7.42106600 7.11666400 2.41793300
C 6.70277700 9.11053000 1.97257800
H 7.58835100 9.41950700 1.42946400
C 5.63829400 9.99854300 2.13054800
H 5.69243600 10.99538200 1.71294500
C 4.50729600 9.58763500 2.82861200
C 3.32056900 10.44174300 3.03995200
C 3.13958300 11.74839700 2.59070600
H 3.91705400 12.26090600 2.04029200
C 1.92728900 12.38705300 2.85966100
H 1.77269300 13.40298900 2.51582800
C 0.90735800 11.73346200 3.55265200
H -0.03455400 12.23117400 3.74079600
C 1.12622300 10.42702900 3.98773600
C 0.17032400 9.54931700 4.69001100
C -1.11958300 9.93122600 5.03361800
H -1.47184500 10.93110000 4.81548400
C -1.95793300 9.00621000 5.66203800
H -2.96799100 9.29121000 5.93339900
C -1.48759100 7.72642300 5.92487900
H -2.10913700 6.97591300 6.39495800
C -0.18151700 7.39859100 5.56232300
H 0.24275700 6.42855200 5.75808200
C 1.35745000 7.87895700 1.47929800
H 1.08515200 8.90701100 1.67848800
C 1.12086800 7.30865500 0.22117800
H 0.64685800 7.90330400 -0.54975200
C 1.51973800 6.00780500 -0.01261700
H 1.37502700 5.54703400 -0.98388800
C 2.11545700 5.26241500 1.02671700
C 2.55034100 3.90797100 0.86086800
H 2.44843800 3.44906400 -0.11632700
C 3.06043600 3.21020600 1.91002600
H 3.37932000 2.18053500 1.79422200
C 3.16343300 3.80667000 3.20805500
C 3.62377200 3.09279100 4.32755100
H 3.92613100 2.05686000 4.22116000
C 3.65743500 3.71146000 5.56007800
H 3.96239400 3.18764300 6.45508300
C 3.27603700 5.05496900 5.65361200
C 2.79140400 5.16352500 3.39546800
C 2.27489300 5.89824300 2.27813000
S -0.97910000 4.30993200 3.28615100
F -1.01723700 2.53451900 5.25633900
F -1.58148400 4.54492400 5.86465800
F 0.50562700 4.07329500 5.47330700
O -0.60087000 5.74418600 3.27286200
O -0.03261300 3.40907900 2.59300900
O -2.41284300 4.03996400 3.03869800
C -0.76232800 3.83469700 5.06523200
O 3.33154200 8.52667900 5.92547400
H 4.07372000 7.62361100 6.84368300

**[1b–OH]$^{+}$, $^{3}$A**

Ru 2.66704100 7.94563400 4.42937700
S 3.18356900 5.59919200 7.33946900
O 4.39414700 6.62584200 7.40245900
O 3.52829400 4.48977500 8.22089100
O 1.90520800 6.29211500 7.50156100
N 4.51289000 8.26744300 3.43715600
N 2.43119600 9.82221300 3.82146200
N 1.98460000 7.21931000 2.50314500
N 2.88101700 5.73197000 4.61753700
N 0.67843400 8.29319500 5.01986100
C 5.51458200 7.39459100 3.27973300
H 5.37066700 6.41158800 3.70908200
C 6.68337600 7.73380200 2.60332600
H 7.47189400 6.99949000 2.49566300
C 6.80505200 9.01786600 2.08135200
H 7.70050000 9.31445800 1.54758200
C 5.76162500 9.92865000 2.24958100
H 5.84310900 10.93038500 1.84851200
C 4.61734100 9.53461800 2.93498600
C 3.44797300 10.41393900 3.15792400
C 3.32086600 11.72860700 2.73347600
H 4.12618100 12.21265000 2.20235500
C 2.11727400 12.40986600 3.00827100
O 1.87281900 13.66760300 2.66204900
C 1.06871600 11.74689100 3.68314100
H 0.14615800 12.28164500 3.86405100
C 1.25163800 10.43829200 4.08450800
C 0.26299700 9.57187500 4.75899500
C -1.02067000 9.97996900 5.09585600
H -1.34451000 10.99253500 4.89233300
C -1.88916100 9.06565700 5.69735900
H -2.89472900 9.37131500 5.96307600
C -1.45448100 7.76923500 5.94150700
H -2.10017000 7.02640900 6.39084000
C -0.15378400 7.41533300 5.58673400
H 0.24424900 6.43132800 5.76818300
C 1.44286700 7.94138500 1.52540500
H 1.20119300 8.97213300 1.74875600
C 1.20151700 7.40962300 0.25131400
H 0.75305500 8.03679400 -0.50908600
C 1.56388700 6.10350100 -0.01054000
H 1.41553700 5.67082900 -0.99410100
C 2.12747600 5.31563000 1.01529900

C 2.52504500 3.95387600 0.81877800
H 2.41814800 3.52253100 -0.17040000
C 3.00736900 3.21650500 1.85377100
H 3.29861700 2.18156900 1.71443800
C 3.11607800 3.77719100 3.16724400
C 3.54628500 3.02166400 4.27135100
H 3.82013400 1.98055800 4.14045700
C 3.58619200 3.60706200 5.51969900
H 3.86869400 3.05219900 6.40327400
C 3.24088400 4.95790100 5.64441200
C 2.77975800 5.13864100 3.38686800
C 2.29388900 5.91556900 2.28369700
S -1.00669100 4.38123900 3.24396500
F -1.11767600 2.57592900 5.18358100
F -1.63189900 4.59130700 5.81921000
F 0.44540400 4.06705500 5.44309800
O -0.58554800 5.80326500 3.25766700
O -0.08055900 3.46302800 2.54598800
O -2.44530800 4.15851800 2.97768700
C -0.82369700 3.87150000 5.01716000
O 3.38023200 8.42228600 6.01069100
H 4.09668800 7.47311100 6.90554000
C 2.88821000 14.43406100 1.96120000
H 3.12328100 13.92437500 1.02142000
H 3.78774200 14.47210700 2.58398000
C 2.31561300 15.81384900 1.72062900
H 3.05386200 16.42264000 1.19195800
H 1.41085900 15.75683300 1.11010200
H 2.07480600 16.30398300 2.66738600

**[1b–OH]$^{+}$, $^{1}$A, open-shell**
Ru 2.66825400 7.94869000 4.42923500
S 3.19526500 5.62104200 7.34568700
O 4.39215700 6.65979300 7.38458100
O 3.55469800 4.52300900 8.23604300
O 1.90920100 6.30115100 7.50742800
N 4.50675700 8.26598000 3.42253700
N 2.43505400 9.82668600 3.82533700
N 1.98677900 7.21559500 2.50966300
N 2.88623600 5.73555900 4.62768500
N 0.68178000 8.29023300 5.00487000
C 5.50273800 7.38811500 3.25474400
H 5.35431100 6.40227200 3.67559200
C 6.67180700 7.72708600 2.57918400
H 7.45589700 6.98939000 2.46290200
C 6.79990500 9.01570100 2.06979200
H 7.69611500 9.31221900 1.53725800
C 5.76228300 9.93114100 2.24872600
H 5.84911500 10.93633900 1.85757400
C 4.61678400 9.53744500 2.93198200
C 3.45095300 10.41939700 3.16235100
C 3.32444200 11.73520700 2.74074700
H 4.12939600 12.21942500 2.20920000
C 2.12116300 12.41655000 3.01703900
O 1.87739000 13.67550900 2.67360500
C 1.07186700 11.75158700 3.68817700
H 0.14819700 12.28483300 3.86799800
C 1.25499200 10.44175600 4.08664000
C 0.26572000 9.57116900 4.75300700
C -1.01909700 9.97418300 5.09110000
H -1.34406700 10.98787600 4.89512600
C -1.88690900 9.05371600 5.68407700
H -2.89340100 9.35572200 5.95042500
C -1.45083200 7.75597400 5.91985200
H -2.09601500 7.00933800 6.36341300
C -0.14954500 7.40620000 5.56457100
H 0.25040500 6.42190100 5.74043500
C 1.44459100 7.93507200 1.52977800
H 1.20423900 8.96676200 1.75032700
C 1.20262000 7.39944600 0.25772400
H 0.75469500 8.02437000 -0.50482500
C 1.56379100 6.09190700 0.00016500
H 1.41457000 5.65626900 -0.98195900
C 2.12676200 5.30647100 1.02824400
C 2.52105300 3.94317200 0.83655500
H 2.41258500 3.50832000 -0.15089300
C 3.00121200 3.20824300 1.87438600
H 3.28919100 2.17187900 1.73893100
C 3.11203900 3.77362000 3.18562200
C 3.54062400 3.02122100 4.29285500
H 3.81042300 1.97855000 4.16601100
C 3.58494700 3.61214900 5.53840700
H 3.86734800 3.06089700 6.42435900
C 3.24527200 4.96496700 5.65707100
C 2.78034900 5.13708600 3.39982300
C 2.29480600 5.91055400 2.29431000
S -1.01559900 4.37652400 3.22715000
F -1.11593200 2.56747400 5.16408900
F -1.62623800 4.58171900 5.80625300
F 0.44880900 4.05795300 5.41773600
O -0.58598400 5.79612700 3.24029700
O -0.09998200 3.45416800 2.52099500
O -2.45743200 4.16296500 2.97126400
C -0.82264000 3.86317200 4.99833500
O 3.37386200 8.44819900 6.00833700
H 4.07913700 7.50239600 6.87223000
C 2.89357900 14.44290600 1.97554700
H 3.12870000 13.93584900 1.03433000
H 3.79306800 14.47852800 2.59852300
C 2.32247400 15.82396500 1.73859400
H 3.06162500 16.43372200 1.21236800
H 1.41817300 15.76966600 1.12717500

H 2.08131500 16.31145900 2.68659100

**[1d–OH]$^+$, $^3$A**
Ru 2.66474400 7.92395600 4.43360100
S 3.18471500 5.57574500 7.33110200
O 4.41680300 6.58128000 7.40660000
O 3.51081400 4.45657200 8.20594900
O 1.91985800 6.29156400 7.49354900
N 4.50760800 8.25079100 3.43692100
N 2.43534000 9.82359800 3.86127400
N 1.96836100 7.21869200 2.50774200
N 2.87516800 5.72230800 4.60881500
N 0.68488700 8.27283200 5.04773400
C 5.50200900 7.37586300 3.25320400
H 5.35564000 6.38418700 3.66090600
C 6.66912500 7.72345200 2.57626300
H 7.45154500 6.98617600 2.44689200
C 6.79718500 9.01753000 2.08263000
H 7.69111200 9.31975800 1.54966100
C 5.76104900 9.93182900 2.27861700
H 5.84556600 10.94187800 1.89958100
C 4.61964700 9.52831400 2.96266600
C 3.46145300 10.41113400 3.21007300
C 3.33358900 11.74387100 2.82552500
H 4.13273000 12.25919800 2.31089600
C 2.14339100 12.40764100 3.12547400
C 1.09658500 11.76547900 3.78628200
H 0.17997900 12.29534100 4.00567300
C 1.26664300 10.43190500 4.15448300
C 0.27813200 9.56118200 4.81837100
C -0.99818900 9.97519300 5.17396700
H -1.31560000 10.99438600 4.99524600
C -1.87093100 9.05547400 5.76307300
H -2.87130500 9.36465300 6.04336200
C -1.44593300 7.75122600 5.97544200
H -2.09421900 7.00447200 6.41436800
C -0.15005900 7.39217800 5.60295300
H 0.23932600 6.40113000 5.76185300
C 1.41987500 7.94497300 1.53678900
H 1.17650300 8.97406300 1.76551300
C 1.17207500 7.41964200 0.26138100
H 0.71853800 8.05041200 -0.49294400
C 1.53438000 6.11528700 -0.00945100
H 1.38088800 5.68789000 -0.99448300
C 2.10407700 5.32233800 1.00916200
C 2.50069300 3.96147100 0.80445500
H 2.38922300 3.53481900 -0.18620500
C 2.98718200 3.21883400 1.83384500
H 3.27723300 2.18448300 1.68820700
C 3.10182500 3.77307000 3.14952700
C 3.53580600 3.01307300 4.24902200
H 3.80852300 1.97230800 4.11340900
C 3.58040900 3.59338500 5.49979100
H 3.86463300 3.03392700 6.37990200
C 3.23736300 4.94391400 5.63215800
C 2.76755600 5.13372400 3.37607000
C 2.27701400 5.91586800 2.27937600
S -1.01711100 4.38826300 3.24845300
F -1.12248500 2.56348700 5.17074800
F -1.63786300 4.57223400 5.82646300
F 0.43946000 4.05414100 5.44134700
O -0.59544100 5.81029300 3.27739000
O -0.09148800 3.47829300 2.53934700
O -2.45586300 4.16884800 2.98191700
C -0.83080100 3.86051900 5.01610000
O 3.38990000 8.39177200 6.01034000
H 4.13827800 7.43567200 6.92887200
C 1.96101200 13.83969200 2.66719400
F 1.43381200 13.87068600 1.42829500
F 1.13088900 14.51170200 3.48398900
F 3.13561300 14.49331800 2.62996200

**[1d–OH]$^+$, $^1$A, open-shell**
Ru 2.66829600 7.92720600 4.43830000
S 3.17986700 5.57796600 7.33564000
O 4.40456200 6.58933600 7.40825000
O 3.50948200 4.46033600 8.21144100
O 1.91052400 6.28686800 7.49602600
N 4.50689400 8.25018100 3.43579300
N 2.43688700 9.82292800 3.86269200
N 1.96915800 7.22065600 2.51459500
N 2.87567900 5.72409800 4.61288800
N 0.68904500 8.27239600 5.04915100
C 5.49935600 7.37346400 3.24984600
H 5.35335700 6.38263500 3.65962800
C 6.66448400 7.71880800 2.56838000
H 7.44549400 6.98034600 2.43730100
C 6.79265800 9.01218600 2.07280700
H 7.68512300 9.31246700 1.53631700
C 5.75857300 9.92825600 2.27138900
H 5.84296900 10.93773900 1.89081500
C 4.61899700 9.52701100 2.95975300
C 3.46216800 10.41033400 3.20919200
C 3.33361800 11.74274900 2.82425600
H 4.13214900 12.25770400 2.30829000
C 2.14384400 12.40680600 3.12557600
C 1.09781700 11.76495000 3.78805300
H 0.18140800 12.29481800 4.00826600
C 1.26824200 10.43173300 4.15688300
C 0.28080100 9.56070600 4.82094700
C -0.99608600 9.97282800 5.17635000
H -1.31442500 10.99194500 4.99887600

C -1.86822400 9.05114800 5.76334800
H -2.86910300 9.35877600 6.04350200
C -1.44226100 7.74678700 5.97327400
H -2.09039100 6.99874600 6.41021200
C -0.14587500 7.38941100 5.60120400
H 0.24413800 6.39839800 5.75830600
C 1.41942300 7.94722000 1.54424000
H 1.17382800 8.97545100 1.77419100
C 1.17301400 7.42317600 0.26812500
H 0.71862000 8.05432200 -0.48537300
C 1.53748600 6.11975100 -0.00466800
H 1.38499100 5.69363800 -0.99040200
C 2.10759200 5.32617300 1.01320600
C 2.50511900 3.96567100 0.80776200
H 2.39481200 3.53977600 -0.18334800
C 2.99046500 3.22214300 1.83714100
H 3.28071200 2.18793700 1.69089200
C 3.10335100 3.77522300 3.15345500
C 3.53514300 3.01478200 4.25354400
H 3.80774900 1.97395100 4.11816000
C 3.57750300 3.59472000 5.50462500
H 3.85971500 3.03493900 6.38520800
C 3.23514000 4.94539800 5.63711100
C 2.76926800 5.13582800 3.38011800
C 2.27935000 5.91850200 2.28402700
S -1.01676100 4.39033500 3.24306500
F -1.12374000 2.56105400 5.16096500
F -1.63634000 4.56896600 5.82155100
F 0.44016800 4.04907100 5.43463100
O -0.59483800 5.81224100 3.27576900
O -0.09127100 3.48211600 2.53157000
O -2.45555200 4.17190900 2.97603900
C -0.83044800 3.85805800 5.00932200
O 3.40240200 8.39570100 6.01096200
H 4.12075900 7.44132300 6.91889900
C 1.96112300 13.83873700 2.66697300
F 1.43344500 13.86924400 1.42829800
F 1.13132100 14.51080900 3.48397400
F 3.13570100 14.49230000 2.62911000

**$[1a–OH]^+$, $^1A$, closed-shell, $SO_3$---HO**
Ru 2.87920600 7.96285800 4.44940000
S 3.15941300 6.57666700 7.35180900
O 4.51151200 7.10515300 7.65407400
O 2.43280700 5.93956400 8.45051200
O 2.29144500 7.61204600 6.61144000
N 4.23816000 8.47030800 2.88925600
N 2.52108900 9.94488400 4.07251400
N 2.11011400 6.96502800 2.69613300
N 3.16028400 5.87989800 4.83871000
N 0.79067200 8.19967900 4.68309300
C 5.04603400 7.58630300 2.29260900
H 4.84112300 6.53769000 2.45760100
C 6.11296600 7.99794700 1.50221400
H 6.74147900 7.25349400 1.02996000
C 6.35022000 9.36198500 1.34824600
H 7.17844100 9.71296900 0.74383000
C 5.51150600 10.27647700 1.98092500
H 5.67345100 11.34116700 1.87323100
C 4.45519700 9.80743400 2.75559900
C 3.45395200 10.65418600 3.40982600
C 3.32173500 12.03784100 3.30265600
H 4.06405500 12.62709200 2.78046900
C 2.19360200 12.64510500 3.85537500
H 2.07612400 13.72032100 3.79071500
C 1.18631300 11.87176000 4.43207500
H 0.27263400 12.32850900 4.78939100
C 1.37046500 10.49194100 4.50711200
C 0.38441100 9.48216500 4.89929400
C -0.89747200 9.74404700 5.36756000
H -1.21373600 10.76454800 5.54164000
C -1.76396400 8.67548100 5.59881100
H -2.76826800 8.86290000 5.96084800
C -1.33260500 7.37558500 5.35232100
H -1.97705400 6.51944600 5.49676700
C -0.03199500 7.16947300 4.90556200
H 0.35308800 6.18103400 4.71408900
C 1.53176500 7.55836400 1.65475100
H 1.28631700 8.60777300 1.75537800
C 1.25953300 6.85024600 0.47474700
H 0.78706400 7.37307100 -0.34739300
C 1.57377000 5.50679500 0.38284300
H 1.35078500 4.94532600 -0.51688000
C 2.17096400 4.85768500 1.48331100
C 2.48646200 3.45702100 1.54368400
H 2.28563800 2.85230400 0.66735900
C 2.97764900 2.88102600 2.68082600
H 3.16815700 1.81450600 2.71899600
C 3.20792400 3.66210500 3.86298900
C 3.54760200 3.14690000 5.13476500
H 3.72446000 2.08384200 5.25526300
C 3.57148400 3.98784900 6.23789400
H 3.72844400 3.60657200 7.23904000
C 3.35867000 5.35729800 6.04045100
C 2.99152000 5.05300300 3.78176400
C 2.43430600 5.64564900 2.61844300
S -1.39274300 4.33621700 2.42011000
F -1.20444900 2.28082000 4.09562300
F -1.71258500 4.16850900 5.04977600
F 0.32167500 3.80772300 4.37512300
O -0.89635100 5.72687900 2.56152700
O -0.63387100 3.49097300 1.47119900

O -2.86405700 4.20153700 2.34804900
C -0.98123800 3.59925000 4.07185200
O 4.46963200 8.55559500 5.33604200
H 4.69680100 8.01945500 6.12645100

**[1b–OH]$^{+}$, $^{1}$A, closed-shell, $SO_3$---HO**
Ru 2.85475400 7.96477400 4.37653800
S 3.23006300 6.22696100 7.52569500
O 4.55076100 6.95147600 7.49443500
O 3.10204400 5.27787400 8.64208400
O 2.07763600 7.13672400 7.29836200
N 4.43118100 8.33187500 3.04195500
N 2.52399500 9.86807100 3.80892900
N 2.18262100 7.01829500 2.60640800
N 3.04522600 5.82904800 4.86084000
N 0.84484600 8.19056200 4.77144100
C 5.32533900 7.41077700 2.66386500
H 5.16760000 6.39884900 3.01187900
C 6.41709700 7.75304800 1.87315400
H 7.12513600 6.98582800 1.58649200
C 6.57593400 9.08047400 1.48260300
H 7.41894400 9.37671000 0.86942500
C 5.63998300 10.03155800 1.88518400
H 5.74023300 11.06635600 1.58434600
C 4.56335000 9.63801000 2.67265700
C 3.47334000 10.52034900 3.11196200
C 3.32230000 11.86717500 2.81084500
H 4.08158900 12.38903900 2.24804700
C 2.14926200 12.52037600 3.23738500
O 1.88681800 13.80490600 3.03037400
C 1.14157500 11.78683900 3.90279300
H 0.21965800 12.28547600 4.17098000
C 1.35816100 10.44949600 4.17021200
C 0.40852300 9.49260700 4.75904800
C -0.85341200 9.80792900 5.23427700
H -1.19205400 10.83586000 5.23898600
C -1.68051300 8.78022400 5.69866500
H -2.67265000 9.01423300 6.06728000
C -1.23254600 7.46464100 5.66885000
H -1.85334900 6.64202000 5.99525900
C 0.04623300 7.19802600 5.19261200
H 0.44801600 6.19959900 5.15075100
C 1.68601500 7.64486300 1.54167200
H 1.50464400 8.70791400 1.63011800
C 1.41576100 6.95884200 0.35088100
H 1.00514000 7.50640700 -0.48815900
C 1.67894300 5.60522600 0.26891900
H 1.48202900 5.05606700 -0.64498800
C 2.19855700 4.92520900 1.38944200
C 2.47150700 3.51892500 1.41120200
H 2.29652700 2.94775200 0.50696700
C 2.90865200 2.90728500 2.54736200
H 3.08981500 1.83849000 2.56707700
C 3.10082200 3.65050700 3.75789300
C 3.42825200 3.05449600 4.99259900
H 3.59773000 1.98463500 5.04649000
C 3.47212200 3.83405300 6.13101700
H 3.64750900 3.41140100 7.11134200
C 3.26904800 5.21697100 6.02411600
C 2.89580800 5.04993500 3.74721500
C 2.43026000 5.68218700 2.55657300
S -1.32644000 4.63132300 2.57370400
F -1.39368300 2.49873200 4.15614200
F -1.75570300 4.38094300 5.18314800
F 0.25917800 3.85619500 4.56048200
O -0.66385600 5.94497100 2.76954500
O -0.65967600 3.73088800 1.60772700
O -2.80125300 4.68604000 2.47434900
C -1.04435000 3.78915900 4.20399900
O 4.15206200 8.57852700 5.56299000
H 4.39267100 7.92765600 6.30415500
C 2.85799900 14.63494100 2.33946300
H 3.01661000 14.22542300 1.33653900
H 3.80154300 14.60222600 2.89382700
C 2.28804100 16.03564300 2.28720300
H 2.99388700 16.69245600 1.77198600
H 1.34058900 16.04838800 1.74251900
H 2.12339500 16.42479100 3.29515200

**[1d–OH]$^{+}$, $^{1}$A, closed-shell, $SO_3$---HO**
Ru 2.91284700 7.91184900 4.44981300
S 3.14149800 6.52973100 7.35082000
O 4.50125100 7.02927000 7.66521200
O 2.38660100 5.91552100 8.44269700
O 2.30432200 7.58361900 6.59676400
N 4.29822400 8.38473300 2.89660100
N 2.60675400 9.90290600 4.06641400
N 2.13251000 6.93244900 2.69067200
N 3.14558900 5.82819400 4.83998100
N 0.82801800 8.20212500 4.66753900
C 5.09065400 7.48261600 2.30795600
H 4.85640800 6.43896900 2.46543200
C 6.17968100 7.86948600 1.53394600
H 6.79501600 7.11031400 1.06783400
C 6.45544400 9.22665000 1.38853500
H 7.30153900 9.55792900 0.79804800
C 5.63107400 10.16112100 2.01202100
H 5.82300300 11.22143000 1.91043000
C 4.55265300 9.71591100 2.76873700
C 3.56301300 10.58550100 3.41024200
C 3.46743500 11.97072200 3.29716500
H 4.23100700 12.54471400 2.78969200

C 2.34736300 12.60087000 3.83845900
C 1.31488100 11.86006400 4.41036600
H 0.41684800 12.34553700 4.76768700
C 1.46570700 10.47702900 4.48877600
C 0.45158200 9.49485100 4.87820700
C -0.82569100 9.79154000 5.33646400
H -1.11812000 10.81998800 5.50573500
C -1.72107000 8.74545400 5.56360600
H -2.72279600 8.95933700 5.91781600
C -1.32069600 7.43512700 5.32304100
H -1.98742300 6.59573700 5.46479300
C -0.02167200 7.19430100 4.88663000
H 0.33997300 6.19585600 4.70174200
C 1.57145400 7.53647400 1.64593900
H 1.34476800 8.59021200 1.74390600
C 1.29174300 6.83290700 0.46489500
H 0.83413500 7.36459800 -0.35993200
C 1.57943600 5.48342000 0.37544200
H 1.35002600 4.92595400 -0.52515900
C 2.15716900 4.82312600 1.47971900
C 2.44288900 3.41627400 1.54367000
H 2.23501000 2.81482500 0.66675400
C 2.91502800 2.83144900 2.68443500
H 3.08288600 1.76123800 2.72488900
C 3.15441000 3.60890500 3.86718800
C 3.47518600 3.08875400 5.14188500
H 3.62936000 2.02250500 5.26505200
C 3.50903700 3.93051100 6.24428300
H 3.65162100 3.54734800 7.24685000
C 3.32525700 5.30353400 6.04388400
C 2.96744500 5.00378800 3.78263300
C 2.43044000 5.60657600 2.61544100
S -1.42075100 4.38934400 2.40351500
F -1.29213300 2.33631900 4.08771100
F -1.75979200 4.23968000 5.03165700
F 0.26894900 3.82707500 4.37031000
O -0.89190800 5.76832300 2.54404100
O -0.67615500 3.52313000 1.46233900
O -2.89424400 4.29001900 2.32252400
C -1.03705200 3.64862500 4.06021200
O 4.51160900 8.47077100 5.34327300
H 4.72209400 7.93451400 6.13835400
C 2.20097800 14.10063400 3.69900600
F 1.62730800 14.41070800 2.52035500
F 1.43043100 14.60874800 4.67690000
F 3.39841800 14.71145100 3.74484700

**[1a–O], $^{3}$A**

Ru 2.62910400 7.94614000 4.41057200
S 3.28913100 5.71255500 7.36463400
O 4.54889700 6.49213000 7.44693800
O 3.29405700 4.49056600 8.21224200
O 2.03216900 6.49453500 7.47661100
N 4.46410300 8.30584700 3.40816400
N 2.33296100 9.81841900 3.76155300
N 1.93857600 7.18258600 2.47641800
N 2.90465600 5.76728400 4.61702000
N 0.63751000 8.26014400 5.02112600
C 5.49694600 7.46645000 3.27373500
H 5.38147100 6.48849600 3.72380300
C 6.65787900 7.83090800 2.59619400
H 7.47258400 7.12282300 2.50822600
C 6.73895300 9.10774500 2.04835200
H 7.62795800 9.42447400 1.51510000
C 5.66312100 9.98412900 2.19070800
H 5.71179600 10.98059700 1.77108500
C 4.52756500 9.56349800 2.87731800
C 3.32804400 10.40913200 3.06750000
C 3.13348800 11.70146200 2.58080500
H 3.90652900 12.20832900 2.01874800
C 1.91111000 12.33063400 2.82408800
H 1.74429000 13.33463900 2.45175600
C 0.89605700 11.67913800 3.52531600
H -0.05581800 12.16622700 3.69074500
C 1.13210900 10.38651900 3.99582100
C 0.17824100 9.51136200 4.70940100
C -1.11675100 9.89337700 5.03870800
H -1.47505400 10.88526600 4.79466400
C -1.95044400 8.97967500 5.68801000
H -2.96403400 9.26363700 5.94786900
C -1.46883000 7.71238300 5.98983500
H -2.08637600 6.97134100 6.48025100
C -0.15831000 7.38579900 5.64072800
H 0.28353400 6.43139000 5.87642100
C 1.37821600 7.87137700 1.48678400
H 1.10779800 8.89921800 1.69295500
C 1.14850200 7.31377600 0.22099600
H 0.68494000 7.91645700 -0.55040800
C 1.53930100 6.01132900 -0.01667800
H 1.39747800 5.55600700 -0.99129600
C 2.12306300 5.25559000 1.02178000
C 2.54512700 3.89929100 0.85019800
H 2.44014700 3.44364600 -0.12852000
C 3.04776200 3.19646500 1.90004600
H 3.35712600 2.16346400 1.78263400
C 3.15861300 3.78917400 3.19930100
C 3.61187600 3.06461100 4.31867700
H 3.90617300 2.02645600 4.20473700
C 3.64164200 3.67739600 5.54872500
H 3.92923500 3.14918600 6.44678200
C 3.27135700 5.03028200 5.66799800
C 2.79971400 5.14410400 3.39566700
C 2.28271500 5.88409800 2.27998900

S -1.06486700 4.35074000 3.26700600
F -1.04742400 2.53937600 5.20185200
F -1.59059800 4.53806400 5.86559400
F 0.48345600 4.07030300 5.40695800
O -0.70576200 5.78874600 3.26511700
O -0.13449600 3.46959300 2.52772200
O -2.50410100 4.06768100 3.05977100
C -0.79275400 3.84462100 5.02956700
O 3.31176400 8.53952700 5.95367700

**[1b–O], $^{3}$A**
Ru 2.69783000 7.90494900 4.47829700
S 3.27838900 5.57362800 7.37995200
O 4.55716200 6.31832300 7.48652400
O 3.24977500 4.33127500 8.19745000
O 2.04040700 6.38328700 7.50733900
N 4.54832200 8.24909200 3.49620400
N 2.45262300 9.79227800 3.86608600
N 2.00607600 7.20586300 2.51861000
N 2.91036100 5.70869200 4.63260900
N 0.70653000 8.25591900 5.07402300
C 5.56176900 7.38836400 3.34940600
H 5.41926000 6.40400500 3.77747600
C 6.73516200 7.73899200 2.68633600
H 7.53332600 7.01365400 2.58760200
C 6.84951400 9.02483500 2.16629500
H 7.74880700 9.33189400 1.64475800
C 5.79337400 9.92291800 2.32080400
H 5.86895300 10.92604000 1.92154300
C 4.64403400 9.51563500 2.99173000
C 3.45911800 10.38305800 3.19255900
C 3.31171900 11.68443500 2.72710200
H 4.11062400 12.16599400 2.18412100
C 2.09515600 12.34953600 2.97295400
O 1.82847400 13.59637600 2.58635100
C 1.05476200 11.68609700 3.65608000
H 0.11902200 12.20627800 3.81072000
C 1.26083400 10.39089900 4.09674700
C 0.27789700 9.52243400 4.78218500
C -1.01234400 9.92545300 5.10453200
H -1.34586300 10.92963600 4.87577400
C -1.87264200 9.01790400 5.72654800
H -2.88286800 9.31856600 5.98081500
C -1.42182900 7.73456900 6.00892100
H -2.06087500 6.99783200 6.47773700
C -0.11540000 7.38671000 5.66733100
H 0.30339800 6.41814200 5.88785000
C 1.47737500 7.93631800 1.54166000
H 1.24322000 8.96823700 1.77025700
C 1.23615300 7.41631500 0.26209600
H 0.79994500 8.05264900 -0.49819200
C 1.58026300 6.10597400 -0.00228900
H 1.42806800 5.67750100 -0.98744700
C 2.12956900 5.30675000 1.02228400
C 2.50087900 3.93960900 0.82234500
H 2.38228700 3.50957400 -0.16636400
C 2.97354600 3.19602400 1.85788300
H 3.24496400 2.15510200 1.71865600
C 3.10209900 3.75590600 3.17008900
C 3.52653000 2.99026700 4.27329900
H 3.78468600 1.94528800 4.13607600
C 3.57394200 3.57330300 5.51701300
H 3.84085200 3.01532400 6.40336000
C 3.24805700 4.93462200 5.66665100
C 2.79057700 5.11822500 3.39641000
C 2.30535400 5.90099400 2.29509900
S -1.12301800 4.46070300 3.19911400
F -1.15535900 2.58293300 5.06920200
F -1.60960200 4.57658700 5.80974800
F 0.43816700 4.04361200 5.30838300
O -0.67419300 5.87265900 3.23429900
O -0.26534500 3.54746600 2.41222900
O -2.58118600 4.27512900 3.01499800
C -0.84985600 3.88232200 4.93937100
O 3.37833400 8.44121800 6.04183600
C 2.83609500 14.35405200 1.87157100
H 3.08973100 13.82047500 0.94952900
H 3.73119700 14.42801000 2.49822000
C 2.24590400 15.71723100 1.58081400
H 2.97935400 16.32091700 1.03974700
H 1.34776100 15.62693300 0.96446000
H 1.98793800 16.23350600 2.50905800

**[1d–O], $^{3}$A**
Ru 2.68777600 7.89489400 4.45942500
S 3.29199100 5.60169800 7.37047600
O 4.56563000 6.35735600 7.46061700
O 3.27670200 4.36775800 8.19990600
O 2.04909600 6.40447900 7.49475700
N 4.53447900 8.24188800 3.47410100
N 2.43648900 9.79060400 3.86081900
N 1.99193500 7.19025700 2.50773600
N 2.91121000 5.71110000 4.62443400
N 0.70074600 8.23556400 5.06855000
C 5.54674600 7.38263200 3.31629800
H 5.40728200 6.39505300 3.73759700
C 6.71718100 7.73916200 2.64966600
H 7.51463500 7.01440100 2.54181700
C 6.82986500 9.02826600 2.13843200
H 7.72663500 9.33875900 1.61480400
C 5.77462200 9.92589400 2.30474100
H 5.84806600 10.93202200 1.91266300

C 4.62959000 9.51196500 2.97844100
C 3.44913500 10.37802000 3.19086000
C 3.28872900 11.68784200 2.74257400
H 4.07158700 12.19614200 2.19688300
C 2.07801000 12.33151100 3.00142000
C 1.04045800 11.68606800 3.67292200
H 0.09867700 12.19088400 3.83850800
C 1.24674800 10.37617200 4.10554200
C 0.26904400 9.50414100 4.78821200
C -1.01833900 9.90680700 5.12087700
H -1.35397500 10.91256700 4.90269800
C -1.87505200 8.99439100 5.74220400
H -2.88344700 9.29336000 6.00496200
C -1.42204800 7.71004500 6.01268600
H -2.05790700 6.97013700 6.48078100
C -0.11665600 7.36334600 5.66110400
H 0.30254400 6.39355200 5.87400300
C 1.45390800 7.91226400 1.52951300
H 1.21423100 8.94401100 1.75258600
C 1.20829600 7.38374500 0.25444900
H 0.76478700 8.01374500 -0.50680600
C 1.55721100 6.07349600 -0.00435400
H 1.40148600 5.63889600 -0.98622100
C 2.11548800 5.28255100 1.02179800
C 2.49106400 3.91547700 0.82916300
H 2.36949400 3.47860300 -0.15612600
C 2.97069100 3.18000700 1.86744300
H 3.24485700 2.13914000 1.73366500
C 3.10286500 3.74861700 3.17552600
C 3.53397700 2.99276400 4.28298600
H 3.79492200 1.94762700 4.15276600
C 3.58480500 3.58537200 5.52221400
H 3.85730800 3.03486600 6.41154900
C 3.25588300 4.94667200 5.66353300
C 2.78790100 5.11124000 3.39309800
C 2.29541900 5.88505700 2.28977600
S -1.11774300 4.44425400 3.21349500
F -1.14324400 2.56142900 5.07901000
F -1.59406800 4.55351000 5.82615900
F 0.45152100 4.02145200 5.31537400
O -0.66808400 5.85620100 3.25238500
O -0.26230900 3.53412500 2.42085900
O -2.57633800 4.26005000 3.03350800
C -0.83822700 3.86074000 4.95105600
O 3.37235500 8.44203300 6.01990800
C 1.90188700 13.77251500 2.57668100
F 0.61120400 14.05622000 2.32325500
F 2.31929900 14.60501600 3.55112800
F 2.61638100 14.04912700 1.47029000

**[1a–O], $^{1}$A, diamagnetic**

Ru 2.66217900 7.92371000 4.49007700
S 3.43095100 5.70887600 7.29820900
O 4.54710000 6.68790800 7.25837500
O 3.72637800 4.51097500 8.12382100
O 2.08442600 6.28249600 7.54347600
N 4.52229300 8.29592900 3.57160000
N 2.30858400 9.72613000 3.70394400
N 1.93806100 7.23827300 2.48259400
N 2.92234400 5.77619300 4.56362800
N 0.66610100 8.22852100 5.11800700
C 5.60517900 7.51234500 3.56674500
H 5.52608200 6.58301400 4.11703000
C 6.77203500 7.87105600 2.89372300
H 7.62703400 7.20636400 2.91166000
C 6.80727400 9.08385200 2.21277600
H 7.69794400 9.39207600 1.67746800
C 5.68196700 9.90888100 2.22787900
H 5.69359800 10.85894000 1.70917500
C 4.54630400 9.49664200 2.91851800
C 3.30729500 10.30103600 2.99987100
C 3.08123600 11.55389800 2.43261800
H 3.84995600 12.05214300 1.85732700
C 1.83549500 12.15725600 2.62038700
H 1.64528300 13.13075100 2.18358600
C 0.83008300 11.52798800 3.35637400
H -0.13135300 12.00532200 3.49066600
C 1.09144500 10.27279000 3.90731100
C 0.16342100 9.42626000 4.68715600
C -1.14910600 9.79254000 4.96141300
H -1.53818500 10.74206300 4.61648100
C -1.96096100 8.91678400 5.68614000
H -2.98752800 9.18712900 5.90627700
C -1.43774000 7.70294200 6.11042000
H -2.03635300 6.98901300 6.66121600
C -0.11136400 7.39086700 5.80546600
H 0.35657100 6.47396700 6.13031800
C 1.36732400 7.93992200 1.50711600
H 1.10706500 8.96744000 1.71921300
C 1.10505100 7.40452800 0.23770400
H 0.63633000 8.02923800 -0.51290600
C 1.46535500 6.10028800 -0.02938400
H 1.29606200 5.66022200 -1.00642100
C 2.05269500 5.32426800 0.99056500
C 2.43869700 3.95982800 0.79674300
H 2.29970800 3.51805100 -0.18409500
C 2.94941400 3.23270400 1.82554300
H 3.23259600 2.19456500 1.68998200
C 3.10669500 3.81000700 3.12658000
C 3.58452100 3.07177100 4.22427200
H 3.85748500 2.02968400 4.09509000
C 3.67712400 3.67860300 5.45558300

H 4.00324700 3.14513600 6.33689000
C 3.33133200 5.03284000 5.60008200
C 2.77583700 5.16998700 3.34081300
C 2.25139400 5.93579100 2.25186100
S -1.12130800 4.49042000 3.29204300
F -1.03458100 2.59748200 5.14328600
F -1.51918300 4.57031100 5.91909900
F 0.52397100 4.10110900 5.34055300
O -0.71588700 5.91531800 3.32201300
O -0.26233200 3.60961400 2.46972800
O -2.57836400 4.25957800 3.15513500
C -0.77074500 3.90681300 5.01618900
O 3.27969800 8.61976600 6.00308800

**[1b–O], $^1$A, diamagnetic**
Ru 2.73051200 7.88005800 4.60591000
S 3.37661800 5.47344700 7.30138800
O 4.54517500 6.38872000 7.34184800
O 3.58695600 4.21509000 8.06131600
O 2.05786700 6.10478400 7.55536200
N 4.61363300 8.22879500 3.72239300
N 2.45302700 9.71723400 3.90071400
N 2.00351900 7.31577900 2.55641900
N 2.91242200 5.71459900 4.56624400
N 0.73724000 8.22964600 5.22308600
C 5.66883000 7.40839300 3.69363800
H 5.55134200 6.45904400 4.20146100
C 6.85504300 7.75460400 3.04887500
H 7.68610900 7.05992600 3.04589700
C 6.94025900 8.99420700 2.42281200
H 7.84724300 9.29467500 1.91099300
C 5.84377500 9.85632800 2.46265800
H 5.89582100 10.82708800 1.98656800
C 4.68632700 9.45474800 3.12211900
C 3.47044500 10.29496800 3.22661100
C 3.30586400 11.56897000 2.70172100
H 4.10737500 12.04429700 2.15717000
C 2.07054400 12.22037400 2.89726300
O 1.78545800 13.43588400 2.44241700
C 1.03437700 11.57842500 3.61055100
H 0.09477500 12.09774300 3.74189400
C 1.25068400 10.30770700 4.10923200
C 0.28220600 9.46244200 4.84284800
C -1.01949900 9.86253700 5.12076300
H -1.36962800 10.84065000 4.81601100
C -1.87014700 8.98461600 5.79590400
H -2.88893100 9.28140100 6.01836600
C -1.39545000 7.73394000 6.16853100
H -2.02571500 7.01752700 6.67936100
C -0.07804000 7.38905600 5.86308500
H 0.35385300 6.44201100 6.14901100
C 1.46591300 8.08740800 1.61559500
H 1.23589300 9.10954100 1.88182100
C 1.20112300 7.63045100 0.31641200
H 0.76032900 8.30949900 -0.40334100
C 1.52399200 6.33192500 -0.01835000
H 1.35230500 5.95145500 -1.01975500
C 2.07524800 5.48343200 0.96335300
C 2.42165400 4.12022400 0.69934900
H 2.28200400 3.73673200 -0.30563600
C 2.89733500 3.32325600 1.69257200
H 3.15084100 2.28578600 1.50337500
C 3.05537400 3.82394100 3.02503400
C 3.49387200 3.01148000 4.08597100
H 3.73516400 1.96955500 3.90326300
C 3.58619700 3.54656900 5.34988100
H 3.88026300 2.95484600 6.20484300
C 3.28078600 4.90142900 5.56486000
C 2.76445600 5.18008700 3.31035100
C 2.27791400 6.01922000 2.25819900
S -1.14226600 4.62659200 3.21502100
F -1.15845900 2.65644100 4.98499300
F -1.58720700 4.61092600 5.83631600
F 0.44868400 4.09236100 5.27424900
O -0.69049000 6.03436500 3.31143000
O -0.29733600 3.75121000 2.37253800
O -2.60334300 4.45163000 3.04180200
C -0.84551900 3.95968200 4.91907100
O 3.35874500 8.45069000 6.16547700
C 2.77510600 14.16993700 1.67551000
H 3.04073400 13.58010700 0.79225900
H 3.66687200 14.30828000 2.29520400
C 2.14929600 15.49412200 1.29464900
H 2.86768200 16.07841900 0.71357200
H 1.25459800 15.33899300 0.68631600
H 1.87749900 16.06498900 2.18619100

**[1d–O], $^1$A, diamagnetic**
Ru 2.70788100 7.94520900 4.38306800
S 2.95402000 5.79837800 7.49781100
O 4.28726000 6.29772800 7.91002300
O 2.51357900 4.59727500 8.25874300
O 1.88821300 6.82301700 7.36641100
N 4.40934100 8.27801100 3.16636600
N 2.47992700 9.86407600 3.89592300
N 2.01936400 7.10356500 2.48999600
N 2.89748700 5.78062200 4.70957100
N 0.72516600 8.25635700 4.90485900
C 5.32799200 7.36898300 2.81610400
H 5.15310000 6.35239000 3.14193800
C 6.45482200 7.71749700 2.07907100
H 7.17589600 6.95448200 1.81379100

C 6.63018400 9.04918400 1.70887700
H 7.49937700 9.35303700 1.13711700
C 5.67676100 9.99387900 2.08154000
H 5.79442200 11.03279800 1.80176600
C 4.56494200 9.58792200 2.81578800
C 3.47079300 10.48314700 3.21940200
C 3.34446300 11.83561000 2.90364000
H 4.11793700 12.35671200 2.35610500
C 2.18380300 12.50234100 3.29104500
C 1.15187000 11.82758600 3.94599200
H 0.23598700 12.34212100 4.20341800
C 1.32431300 10.47761900 4.23723400
C 0.33270900 9.56776200 4.83211500
C -0.93266100 9.94664100 5.25784800
H -1.24017800 10.98341000 5.21044600
C -1.80569800 8.96786200 5.74124600
H -2.79933700 9.24930600 6.07114200
C -1.39625100 7.64010900 5.78617200
H -2.04993200 6.85334000 6.13762000
C -0.10976500 7.31622500 5.36304800
H 0.27648900 6.31298700 5.39340900
C 1.49543200 7.76138500 1.46045300
H 1.24449500 8.80340200 1.62086100
C 1.28696100 7.14873700 0.21666500
H 0.84987200 7.72062100 -0.59265600
C 1.66958200 5.83211900 0.04449000
H 1.54586400 5.33941700 -0.91429700
C 2.22263900 5.11396500 1.12566000
C 2.63850700 3.74779500 1.02977300
H 2.55345500 3.24661300 0.07168400
C 3.10969400 3.08839900 2.12355200
H 3.41145100 2.04860100 2.05840000
C 3.18886000 3.73684600 3.39865100
C 3.59168900 3.06809500 4.57379500
H 3.89057400 2.02623200 4.52469300
C 3.54610300 3.73363600 5.77603400
H 3.76172700 3.23326000 6.71008500
C 3.16959400 5.09216500 5.81712300
C 2.83640500 5.10067900 3.51752100
C 2.35517100 5.79465500 2.35826900
S -1.11770500 4.27414700 3.04653100
F -1.13870300 2.46066600 4.98395700
F -1.62647300 4.47378600 5.64698200
F 0.43223300 3.95267400 5.18267200
O -0.64659500 5.68017400 3.03572200
O -0.27441800 3.32263900 2.29143900
O -2.57811900 4.10544700 2.87014700
C -0.85151700 3.75694700 4.80812300
O 3.74402500 8.34717000 5.76423800
C 2.04646600 13.98183100 3.01812400
F 0.76116600 14.32578700 2.81112400
F 2.49524700 14.70227500 4.06609800
F 2.75744900 14.35220600 1.93662600

**$[Ru–O]^{+}$, for 1a : $^{2}A$**

Ru 2.71763100 7.88151400 4.43441200
S 3.66098200 5.83009400 7.25449300
O 5.05015500 6.28773500 7.15819500
O 3.32226600 4.84324600 8.28962800
O 2.63977300 6.98943200 7.35374000
N 4.57676000 8.28487500 3.50551000
N 2.43528700 9.77926100 3.86778700
N 2.11188100 7.20231100 2.45720100
N 2.93276100 5.73929500 4.60953400
N 0.72240600 8.15130400 5.00654600
C 5.61197600 7.44804600 3.37131900
H 5.48551100 6.45269000 3.77732300
C 6.79229800 7.84298200 2.74710300
H 7.61050700 7.13964900 2.65653300
C 6.88813700 9.14130400 2.25567600
H 7.79301100 9.47940500 1.76423000
C 5.80812400 10.01336600 2.39962800
H 5.86986200 11.02635400 2.02396600
C 4.65314500 9.56652900 3.03201800
C 3.45052400 10.40234000 3.22951500
C 3.26455800 11.71822700 2.81071000
H 4.04872000 12.25524600 2.29433600
C 2.03626400 12.33334500 3.06402500
C 1.00559600 11.64997800 3.71163800
H 0.05222100 12.13083000 3.88525300
C 1.22948900 10.33442200 4.11587500
C 0.27313800 9.42675300 4.77832400
C -1.00919400 9.79826400 5.15779400
H -1.36164600 10.80625500 4.98175500
C -1.83543400 8.85658400 5.77719800
H -2.83802400 9.13565400 6.08020800
C -1.36317300 7.56903000 5.99570400
H -1.97629200 6.80875700 6.45998500
C -0.06924900 7.24752200 5.58937600
H 0.34893600 6.26384300 5.73374500
C 1.64860900 7.92752000 1.44097000
H 1.50276300 8.98551800 1.61298900
C 1.36416700 7.36353400 0.19008000
H 0.98625600 7.99747600 -0.60223000
C 1.58605900 6.01513800 -0.00795600
H 1.39095100 5.55502400 -0.97062400
C 2.06528000 5.22229000 1.05611700
C 2.31059600 3.81673000 0.93302300
H 2.14042000 3.34710300 -0.02927700
C 2.72829100 3.08438800 2.00111700
H 2.90164900 2.01796100 1.91232700
C 2.92899600 3.69967800 3.27805300

C 3.31552200 2.97561000 4.42323300
H 3.47004500 1.90434800 4.35540900
C 3.48319300 3.63268600 5.62382800
H 3.75611200 3.10532400 6.52783900
C 3.28925900 5.02086700 5.67414700
C 2.72866100 5.09429000 3.41988100
C 2.29729200 5.86301100 2.29305900
S -1.21832800 4.58278700 3.05447100
F -1.27983700 2.54921700 4.75773400
F -1.63740900 4.48559500 5.67912400
F 0.37491900 3.92579900 5.07993900
O -0.56269900 5.91033900 3.16026700
O -0.54587400 3.62574300 2.15023700
O -2.69398400 4.62334600 2.95654700
C -0.92959800 3.84051000 4.73156900
O 3.35105900 8.28158200 6.03007200
H 1.87746900 13.35587700 2.74247700

**[Ru–O]$^{+}$, for 1a : $^{4}$A**

Ru 2.79992200 7.95583700 4.41682300
S 3.53778000 5.71080700 7.31274500
O 4.78563700 6.50262300 7.20859800
O 3.65745000 4.51914100 8.18802700
O 2.28068300 6.47304800 7.51461900
N 4.66539700 8.38247600 3.50529200
N 2.50662300 9.84942100 3.84280700
N 2.13835700 7.23747800 2.44140500
N 2.96293100 5.74901700 4.59362200
N 0.78627500 8.21518300 4.97228200
C 5.71205700 7.55974700 3.37249500
H 5.58750300 6.55659000 3.76050400
C 6.89982300 7.97317900 2.77378300
H 7.72530500 7.27794500 2.68469200
C 6.99361000 9.28023500 2.30551800
H 7.90361800 9.63437100 1.83501500
C 5.90317300 10.13896600 2.44636000
H 5.96129300 11.15870100 2.08850300
C 4.74176300 9.67031500 3.05261200
C 3.52767200 10.49411300 3.24010100
C 3.34318400 11.81737600 2.84050100
H 4.13553000 12.36773200 2.35106800
C 2.10840300 12.42249800 3.08150200
C 1.07071500 11.71912600 3.69480800
H 0.11366800 12.19346800 3.86646700
C 1.29378600 10.39530500 4.07434800
C 0.32114100 9.47205100 4.69680100
C -0.99472100 9.81678100 4.98428900
H -1.36274800 10.81008700 4.76216400
C -1.83712000 8.86435900 5.56089800
H -2.86622000 9.11964000 5.78645600
C -1.34427800 7.59479600 5.84037800
H -1.96646300 6.82896100 6.28417700
C -0.01623900 7.30537400 5.53309300
H 0.42970300 6.34630600 5.74824400
C 1.69870200 7.98707400 1.41988000
H 1.64561000 9.05438000 1.58848300
C 1.32364000 7.44712300 0.18547500
H 0.97474000 8.10483100 -0.60024400
C 1.40978000 6.08118500 0.00347700
H 1.12903600 5.61991600 -0.93679800
C 1.86456900 5.27076100 1.06896700
C 1.91881700 3.86430700 0.97799900
H 1.61674400 3.37447100 0.06017300
C 2.34612600 3.10022500 2.06642200
H 2.38475200 2.02072500 1.98369200
C 2.75019600 3.70886800 3.28148100
C 3.20131000 2.94699900 4.37665300
H 3.29597500 1.87070200 4.28648200
C 3.48071500 3.59397900 5.55912200
H 3.78736700 3.06182900 6.44835800
C 3.31794000 4.98398700 5.64880700
C 2.66431000 5.11664200 3.42789500
C 2.21814600 5.90399800 2.29295200
S -0.99772300 4.15761300 3.13714300
F -1.18491400 2.37395800 5.07762600
F -1.65710100 4.41331200 5.67389600
F 0.41181300 3.83010000 5.34833000
O -0.33525100 5.48229000 3.09740900
O -0.26029200 3.08286900 2.39843100
O -2.44591800 4.15792700 2.87335700
C -0.85156500 3.65411200 4.91832100
O 3.40516100 8.51708500 5.99975400
H 1.95084700 13.45141300 2.78017000

**[Ru–O]$^{+}$, for 1b : $^{2}$A**

Ru 2.71319600 7.88638500 4.49353200
S 3.36673400 5.73685000 7.27300300
O 4.37088700 6.88458700 7.10724700
O 3.95071300 4.70913800 8.14879300
O 2.00111300 6.19357900 7.57117500
N 4.56974400 8.22743700 3.54554800
N 2.45768300 9.75662400 3.86130200
N 2.02488500 7.24867800 2.50189600
N 2.89423300 5.73255400 4.58956100
N 0.70818300 8.20624300 5.04539400
C 5.59003700 7.36840800 3.43361900
H 5.44541200 6.38545900 3.86244000
C 6.77774600 7.72420500 2.80078500
H 7.58204800 7.00283800 2.72939500
C 6.89847100 9.00717000 2.27592900
H 7.80971300 9.31567100 1.77691100
C 5.83408900 9.90189100 2.39497400

H 5.91516000 10.90278300 1.99165000
C 4.67040500 9.49392600 3.03622500
C 3.47846200 10.35283100 3.20549900
C 3.32923800 11.64986200 2.74078100
H 4.13408500 12.13793500 2.21272600
C 2.10152200 12.30864200 2.96844200
O 1.83561400 13.54718400 2.58237500
C 1.05061800 11.63756200 3.63440800
H 0.11058300 12.15363400 3.77487500
C 1.25353000 10.34558200 4.07532100
C 0.27221200 9.47166700 4.74946900
C -1.02104800 9.86185700 5.06658100
H -1.36327900 10.86222400 4.83492000
C -1.87371400 8.94746800 5.69120500
H -2.88629700 9.24062000 5.94368600
C -1.41416000 7.66926600 5.97826200
H -2.04660700 6.92900000 6.44946300
C -0.10629600 7.32811700 5.63595700
H 0.31042600 6.35791200 5.85193800
C 1.51585400 7.99112700 1.52184300
H 1.32283000 9.03317300 1.73704400
C 1.24244800 7.46696100 0.25070800
H 0.82344600 8.11494300 -0.50894600
C 1.53209500 6.14244400 -0.00950100
H 1.35294100 5.71560600 -0.99044600
C 2.05988300 5.32990700 1.01612200
C 2.37904900 3.94563300 0.83095200
H 2.23506200 3.51153500 -0.15217300
C 2.83248500 3.18940700 1.86689000
H 3.06292400 2.13882600 1.73141200
C 2.99616200 3.75574300 3.17214900
C 3.42320400 3.00518800 4.28396300
H 3.64190700 1.94918900 4.16832600
C 3.54984500 3.61642500 5.51513500
H 3.85851200 3.07268800 6.39706400
C 3.27428400 4.98488400 5.62738500
C 2.72941700 5.13238300 3.37287100
C 2.26659200 5.92807000 2.27859100
S -1.10998700 4.48736800 3.19756100
F -1.16478900 2.56345100 5.02170800
F -1.59554500 4.54301500 5.81137600
F 0.44559300 4.00142200 5.29346700
O -0.58395700 5.87350600 3.26021700
O -0.30303000 3.55131700 2.38546600
O -2.57558400 4.38409300 3.02187700
C -0.84730900 3.86027000 4.92299200
O 3.31389600 8.33976200 6.07533000
C 2.84754500 14.32493700 1.88535900
H 3.11903400 13.79676500 0.96608400
H 3.72857100 14.40434600 2.52990800
C 2.24178700 15.68032900 1.59431400
H 2.97612600 16.29601900 1.06831000
H 1.35533800 15.58120200 0.96281200
H 1.96317800 16.18757500 2.52137900

**$[Ru–O]^+$, for 1b : $^4A$**

Ru 2.80568600 7.95063700 4.40055100
S 3.53647000 5.72086800 7.31833900
O 4.78954400 6.50427800 7.21404700
O 3.64595700 4.53443100 8.20248400
O 2.28290600 6.49099700 7.51140600
N 4.67100000 8.36860100 3.48102800
N 2.51426500 9.82904900 3.79269600
N 2.13923900 7.21038500 2.43261100
N 2.96489800 5.74028700 4.59759700
N 0.78998900 8.22495800 4.94819000
C 5.71893600 7.54496400 3.36367600
H 5.59468500 6.54856100 3.76887600
C 6.90699100 7.94896400 2.75946900
H 7.73335700 7.25320500 2.68337700
C 6.99981400 9.24787100 2.26894600
H 7.90995100 9.59510100 1.79355200
C 5.90807700 10.10738800 2.39376000
H 5.96651900 11.12072900 2.01834700
C 4.74628500 9.64852200 3.00606100
C 3.52766800 10.47264500 3.17928400
C 3.35658000 11.78344100 2.75164500
H 4.15884700 12.30680100 2.25428900
C 2.11367300 12.40586500 2.97995100
O 1.82382000 13.65584400 2.62885400
C 1.07190200 11.69147300 3.60943900
H 0.12042500 12.18356100 3.75942900
C 1.29915100 10.38769300 4.00931100
C 0.32416200 9.47558100 4.64802900
C -0.99231600 9.82437700 4.92707200
H -1.35976400 10.81345600 4.68553900
C -1.83486800 8.88283000 5.52021400
H -2.86450300 9.14161100 5.73943900
C -1.34163300 7.61874800 5.82426800
H -1.96414600 6.86095600 6.28123600
C -0.01336600 7.32482900 5.52460700
H 0.43349500 6.37049000 5.75854200
C 1.69522000 7.95094200 1.40654600
H 1.64107700 9.01932700 1.56803500
C 1.31693600 7.40035800 0.17767000
H 0.96450500 8.05098800 -0.61237800
C 1.40487200 6.03322800 0.00659000
H 1.12241500 5.56375600 -0.92909800
C 1.86349200 5.23207000 1.07762500
C 1.91952100 3.82528100 0.99773800
H 1.61669700 3.32775700 0.08427100
C 2.34935000 3.07072000 2.09158100

H 2.38896500 1.99057500 2.01784700
C 2.75323300 3.68946400 3.30138000
C 3.20495300 2.93627500 4.40230700
H 3.30031300 1.85936300 4.32024800
C 3.48339600 3.59245900 5.57988200
H 3.78961000 3.06725300 6.47338300
C 3.31920600 4.98298500 5.65880900
C 2.66689400 5.09849900 3.43694000
C 2.21919700 5.87599000 2.29568800
S -0.99919500 4.14761200 3.16086600
F -1.18898900 2.39206100 5.12655500
F -1.64849600 4.44183900 5.69633300
F 0.41620000 3.84322000 5.37196400
O -0.33562900 5.47118900 3.10181100
O -0.26503400 3.06246200 2.43552900
O -2.44844100 4.14716700 2.90170800
C -0.84939900 3.66829800 4.94823300
O 3.41216000 8.52307100 5.97729900
C 2.83204000 14.47135500 1.97902200
H 3.13188400 13.98212800 1.04647600
H 3.70225600 14.54547000 2.63944300
C 2.20958800 15.82682600 1.72231300
H 2.94155900 16.47304200 1.23065800
H 1.33549000 15.73490300 1.07255500
H 1.90560300 16.29736400 2.66084600

**$[Ru–O]^+$, for 1d : $^2A$**
Ru 2.84793400 7.82535900 4.33064100
S 4.33928400 6.21946600 7.03186600
O 5.60940200 6.73540400 6.53852700
O 4.21890100 5.54254300 8.31481400
O 3.22927200 7.44428800 7.11547500
N 4.67465800 8.35955900 3.45102000
N 2.48803400 9.73573700 3.85702000
N 2.24377700 7.00692100 2.47854900
N 3.16052800 5.74952500 4.67918700
N 0.83643700 7.98964700 4.92538300
C 5.74512800 7.57175900 3.28213400
H 5.65165600 6.55131000 3.62947600
C 6.91817600 8.04496300 2.70150500
H 7.76337700 7.37831800 2.58397500
C 6.97397900 9.37257300 2.28634300
H 7.87294700 9.77100500 1.83045000
C 5.85998100 10.19358200 2.46120900
H 5.88873500 11.22804100 2.14444100
C 4.71373100 9.66858600 3.04866200
C 3.47872100 10.44312200 3.27769600
C 3.25356900 11.78054200 2.95248100
H 4.02080500 12.38503900 2.48873400
C 2.00602200 12.32878900 3.24654200
C 1.00067600 11.56941600 3.84741500
H 0.04020900 12.01349600 4.07052300
C 1.27103400 10.23624700 4.14938900
C 0.34572100 9.25930500 4.76209800
C -0.94693800 9.57331400 5.15948800
H -1.33285300 10.57566000 5.02668500
C -1.74135200 8.58288900 5.74201900
H -2.74904100 8.81910800 6.06393800
C -1.23108300 7.30051400 5.89538300
H -1.82038600 6.50226700 6.32463000
C 0.06541800 7.03676000 5.45829600
H 0.49833600 6.04888300 5.53111700
C 1.81020900 7.65543400 1.39913600
H 1.80007200 8.73705900 1.44782000
C 1.38287300 6.97862000 0.24820100
H 1.03882500 7.55279800 -0.60304200
C 1.39674200 5.59821000 0.22533600
H 1.05696000 5.05158400 -0.64639900
C 1.85645800 4.88776200 1.35495500
C 1.91294800 3.45579300 1.42372500
H 1.56938200 2.88982500 0.56566800
C 2.38235100 2.81679200 2.53273600
H 2.42286100 1.73428000 2.57436700
C 2.82798500 3.56020600 3.67434300
C 3.32090400 2.96655400 4.85408900
H 3.37571900 1.88633800 4.93071900
C 3.73785900 3.76265800 5.90753800
H 4.12716400 3.34072900 6.82477800
C 3.64504300 5.15069000 5.76133600
C 2.75974500 4.97452700 3.63348700
C 2.27029800 5.64208800 2.46850700
S -1.63321500 5.14529100 2.43557100
F -1.79627700 2.78300900 3.63728100
F -1.99832500 4.47771000 4.98266200
F -0.04366700 3.94555400 4.19714600
O -0.78767800 6.32016900 2.75863200
O -1.16709800 4.32965200 1.29047600
O -3.09142200 5.39446600 2.47570400
C -1.35652600 4.02260200 3.89017800
O 3.45753600 8.44561800 6.07146100
C 1.71481900 13.76093000 2.85570400
F 1.17493600 13.81345300 1.62186800
F 0.84292800 14.33229500 3.70655700
F 2.83627600 14.50411200 2.84264500

**$[Ru–O]^+$, for 1d : $^4A$**
Ru 2.80279900 7.95233600 4.39503600
S 3.54047900 5.73664000 7.30687200
O 4.78995100 6.52442800 7.19180300
O 3.65838300 4.55366100 8.19369200
O 2.28573800 6.50448500 7.50261800
N 4.66778200 8.38051200 3.48142800

N 2.50942100 9.84698300 3.82317800
N 2.14384700 7.22375600 2.42386700
N 2.96315400 5.75091400 4.58814400
N 0.79141600 8.21368400 4.95768500
C 5.71421800 7.55891400 3.34548000
H 5.59109700 6.55471300 3.73107700
C 6.90155300 7.97475400 2.74601600
H 7.72693500 7.27974200 2.65448700
C 6.99512500 9.28228800 2.28041300
H 7.90434000 9.63759500 1.80946800
C 5.90441700 10.14077200 2.42460400
H 5.96247900 11.16106200 2.06832000
C 4.74500900 9.66947400 3.03144300
C 3.53155900 10.49208700 3.22413900
C 3.35229300 11.81862100 2.83661700
H 4.14478900 12.37692400 2.35774400
C 2.11902200 12.42056100 3.08915800
C 1.07954000 11.72189000 3.70245200
H 0.13095700 12.20573700 3.88995300
C 1.30035800 10.39411300 4.06658600
C 0.32714900 9.47246000 4.68847800
C -0.98653700 9.82051200 4.97952100
H -1.35373300 10.81481200 4.76044700
C -1.82979200 8.86810700 5.55581300
H -2.85772100 9.12495500 5.78443800
C -1.33848400 7.59735400 5.83030400
H -1.96092300 6.83145200 6.27355900
C -0.01147000 7.30499000 5.51840100
H 0.43183800 6.34399900 5.72969400
C 1.71204900 7.96661000 1.39393200
H 1.66472000 9.03585200 1.55132800
C 1.33777500 7.41779800 0.16339800
H 0.99530400 8.07045000 -0.62931200
C 1.41600200 6.04964800 -0.00585800
H 1.13469700 5.58158700 -0.94256500
C 1.86395700 5.24649400 1.06778700
C 1.91115200 3.83876700 0.98962500
H 1.60781700 3.34218900 0.07585400
C 2.33351500 3.08225700 2.08547300
H 2.36709000 2.00192500 2.01242600
C 2.74083700 3.70000000 3.29503100
C 3.18886300 2.94638200 4.39712000
H 3.27854100 1.86886600 4.31696300
C 3.47189500 3.60310800 5.57333200
H 3.77683600 3.07792400 6.46730500
C 3.31563000 4.99459900 5.65031900
C 2.66098600 5.10921000 3.42832500
C 2.21840800 5.88853100 2.28661800
S -1.00085900 4.15222400 3.14289600
F -1.19467000 2.37435500 5.08830200
F -1.66811300 4.41587200 5.67629500
F 0.40168800 3.83105700 5.35899500
O -0.33305500 5.47406400 3.10066300
O -0.26533300 3.07238200 2.40860800
O -2.44797600 4.15612700 2.87454900
C -0.86054200 3.65361800 4.92605100
O 3.41106600 8.52194200 5.97499700
C 1.88786200 13.84429000 2.63053800
F 1.45374900 13.86248300 1.35511000
F 0.96205700 14.45976700 3.38747300
F 3.02255000 14.56507900 2.68578400
